# Supplementary material for: Long-read deep sequencing reveals high rates of multilineage transmission and rapid viral population changes in acute HIV infection
Source: Nat Commun. 2026 May 25;17:6799. doi: 10.1038/s41467-026-73496-0 (PMC13385744; doi:10.1038/s41467-026-73496-0)
Supplement: Supplementary file 1 — Supplementary Information [file 41467_2026_73496_MOESM1_ESM.pdf]

Long-Read Deep Sequencing Reveals High Rates of Multilineage Transmission and Rapid Viral Population Changes in Acute HIV Infection

James I. Mullins[1*]
Wenjie Deng[1^]
Elena E. Giorgi[2]
Craig A. Magaret[2]
Morgane Rolland[3,4]
Tanmoy Bhattacharya[5,6]
Dylan H. Westfall[1^]
Anna E.J. Yssel[8^]
Roger E. Bumgarner[1]
Ben Murrell[7]
Thumbi Ndung'u[9-12]
Merlin L. Robb[3]
Raabya Rossenkhan[2]
Paul T. Edlefsen[2]
Krista L. Dong[11]
Lennie Chen[1]
Asanda Gwashu-Nyangiwe[8]
Hong Zhao[1^]
Ruwayhida Thebus[8]
Nonkululeko Ndabambi[8]
Bruna Galvao[8]
Fredrick Sawe[13,14]
Sorachai Nitayaphan[15]
Talita York[8]
David Matten[8]
Hugh Murrell[8]
Alec P. Pankow[1^]
Michal Juraska[2]
James Ludwig[2]
John Hural[2]
Myron S. Cohen[16]
Lawrence Corey[2]
M. Juliana McElrath[2]
Peter B. Gilbert[2]
Carolyn Williamson[8,17]

Affiliations:

[1] Department of Microbiology, University of Washington, Seattle, WA, US

[2] Vaccine and Infectious Disease Division, Fred Hutchinson Cancer Center, Seattle, WA, US

[3] Henry M. Jackson Foundation for the Advancement of Military Medicine, Inc., Bethesda, MD, US

[4] U.S. Military HIV Research Program, Walter Reed Army Institute of Research, Silver Spring, MD, US

[5] Theoretical Division, Los Alamos National Laboratory, Los Alamos, NM, US

[6] Santa Fe Institute, Santa Fe, NM, US

[7] Department of Microbiology, Tumor and Cell Biology, Karolinska Institutet, Stockholm, SE

[8] Institute of Infectious Diseases and Molecular Medicine, Division of Medical Virology, University of Cape Town, Cape Town, ZA

[9] African Health Research Institute, University of KwaZulu Natal, Durban, ZA

[10] HIV Pathogenesis Programme, The Doris Duke Medical Research Institute, University of KwaZulu-Natal, Durban, ZA

[11] Ragon Institute of Mass General Brigham, Massachusetts Institute of Technology and Harvard University, Cambridge, MA, US

[12] Division of Infection and Immunity, University College London, London, UK

[13] Kenya Medical Research Institute, Walter Reed Army Institute of Research-Africa, Nairobi, KE

[14] Henry M. Jackson Foundation Medical Research International, Nairobi, KE

[15] Armed Forces Research Institute of Medical Sciences, Bangkok, TH

[16] University of North Carolina, Chapel Hill, NC, US

[17] National Health Laboratory Services, ZA

^ Present addresses:

WD, DHW: Vaccine and Infectious Disease Division, Fred Hutchinson Cancer Center, Seattle, WA, USA
AEJY: Sand Technologies, Cape Town, ZA
APP: Department of Microbiology, Icahn School of Medicine at Mount Sinai, New York, NY, US
HZ: Department of Obstetrics and Gynecology, University of Washington, Seattle, WA, US

* Corresponding author:

Department of Microbiology, University of Washington School of Medicine, Seattle, WA, US. email: jmullins@uw.edu

Supplementary Discussion

1. Interestingly, in the second time window evaluated (See Methods), the frequencies of hypermutated sequences became higher in *gag* compared to *env* for Subtype C, whereas for the other subtypes it remained higher in *env*. Comparisons of the frequencies of hypermutated sequences in the second time window across clades were not significant in *gag* and they were significant in *env* only when comparing subtype B to subtype C (Lower in Subtype C; Supplementary Table 4C).

2. There was a significant correlation between the proportion of hypermutated sequences and viral load in *gag* (p=0.03) but not in *env* (p=0.9) in the first time window, and no significant associations were found in the second time window.

3. This method derives from the understanding that prior to the onset of detectable immune-driven selection in the viral proteome (~5-7 weeks post-acquisition; Goonetilleke N, et al. J. Exp. Med. 2009;206(6):1253–72; Liu, Y. et al. J. Virol. 2006;80(19):9519–29; Liu Y, et al. Virology 2006;347(1):140–6), an infection initiated by a single variant is expected to grow exponentially, randomly accumulating mutations, with mutational distances fitting a Poisson distribution. Failure to fit a Poisson indicates evidence of selection or acquisition of multiple lineages. Subsequent partitioning into groupings that each fit a Poisson resulted in the definition of distinct lineages from within these multilineage infections.

4. In 17 instances, only one sequence was reported to represent a minor lineage in GP and/or REN, as shown in Supplementary Table 6. However, in 15 of these instances, recombinants were also detected formed between the major and minor lineage, further confirming the existence of the minor lineage. In one remaining instance, 6 additional sequences were found to represent the minor lineage prior to the conservative removal of 20% of the sequences to avoid detection of products produced by cDNA primer carryover (see Methods). In the final instance, the minor lineage was distinguished by the presence of 7 clustered, unique mutations. In our experience, there is no precedent for such an outlier being derived from a technical artifact.

Supplementary Table 1. HIV subtypes and discordance between GP and REN subtypes		
**Cohort**	**REN Subtypes**	**GP-REN Subtype discordance[2]**
**RV217 (Thailand)**	15x CRF01AE, 1x B	1x B-AE
**RV217 (Kenya)**	3x A1, 3x A1C, 5x A1D, 1x C, 1x D	1x D-A1D, 1x A1-A1C, 2x A1-A1D, 1x A1C-A1D, 1x A1C-D
**FRESH**	13x C	
**HVTN 703[1]**	31x C, 1x G	1x A1G-G
**HVTN 704[1]**	37x B, 4x F1, 1x F2, 5x BF1, 1x A1B	1x BF1A1-BF1, 1x B-BF1, 2x BF1-F1
[1]From the trial control arms (placebo recipients) only. [2]Numbers and identities of discordant subtype determinations are listed for GP-REN.		

Supplementary Table 2. Putative inactivating mutations

Gag

Cohort	Proportion intact	Putative Inactivating mutations (Proportions)[1]			
		Premature stop	Large deletion[2]	Missing 5' end[3]	No ATG[4]
FRESH	0.905	1	0	0	0
RV217 Kenya	0.950	0.99	0.003	0.0001	0.005
RV217 Thailand	0.964	1	0	0	0
HVTN 703 (C)	0.879	0.997	0.001	0	0.002
HVTN 704 (C)	0.977	0.987	0	0	0.013
Overall	*0.943*				

Env

Cohort	Proportion intact	Putative Inactivating mutations (Proportions)[1]			
		Premature stop	Large deletion[2]	Missing 5' end[3]	No ATG[4]
FRESH	0.821	0.986	0.003	0.01	0.0002
RV217 Kenya	0.847	0.989	0.003	0.004	0.003
RV217 Thailand	0.843	0.972	0.017	0.004	0.007
HVTN 703 (C)	0.789	0.968	0.008	0.019	0.004
HVTN 704 (C)	0.903	0.982	0.007	0.0006	0.004
Overall	*0.856*				

[1] These proportions refer to the composition of the sequences that do not appear to be intact, e.g., the 0.905 proportion of sequences from the FRESH cohort. [2] A large deletion was defined as being >20% of the median length of the genes in a given individual. [3] A missing 5’ end was defined as a deletion encompassing at least a portion of the ATG. [4] An alternative start codon, i.e., not an ATG, may be present.

Supplementary Table 3. Data used to assess intact genes[1]

Trial	Gene	Time Pt.	N	Window	COB/EDDI (Days)					
					Min.	1st Q	Median	Mean	3rd Q	Max.
FRESH	Gag	1	13	NA	2	5	9	11.85	19	24
FRESH	Gag	2	13	1	12	16	26	27.00	33	59
FRESH	Gag	3	10	2	19	34.25	55.5	47.10	57.75	61
FRESH	Env	1	13	NA	2	5	9	10.77	19	24
FRESH	Env	2	13	1	12	16	23	24.23	30	44
FRESH	Env	3	10	2	19	34.25	51.5	46.20	57.5	61
RV217	Gag	1	29	NA	2	4	5	5.34	7	14
RV217	Gag	2	29	NA	5	10	15	16.52	18	51
RV217	Gag	3	29	1	11	15	24	29.21	45	61
RV217	Gag	4	16	2	15	19	27	30.25	39.25	61
RV217	Gag	5	7	NA	26	30	34	34.86	39	46
RV217	Env	1	29	NA	2	4	5	5.72	7	14
RV217	Env	2	29	1	5	11	15	16.90	18	51
RV217	Env	3	29	2	11	17	24	29.41	45	61
RV217	Env	4	16	NA	15	23	31	32.19	39.25	61
RV217	Env	5	5	NA	26	29	35	35.80	43	46
V703	Gag	1	32	1	7	18	31	29.72	35.25	89
V703	Gag	2	28	2	26	32.75	46	49.18	57	109
V703	Env	1	32	1	7	18	31	28.81	35.25	89
V703	Env	2	28	2	26	32.75	46	47.04	55.5	108
V704	Gag	1	47	1	8	18	31	32.62	37.5	135
V704	Gag	2	40	2	13	33.5	38.5	46.35	47	187
V704	Gag	3	13	NA	20	46	56	58.54	60	151
V704	Env	1	47	1	8	18	28	32.02	37	135
V704	Env	2	41	2	13	34	38	46.10	46	187
V704	Env	3	13	NA	20	46	56	58.54	60	151

[1] Samples in the blue font were included in analysis using the 1st time window, and red font if included in analysis using the 2nd time window. Samples in black font were not used in these analyses. NA = Not applicable, i.e., outside of windows 1 or 2.

Supplementary Table 4

A. Proportion of genes that were intact (Generalized Linear Model; GLM)

Comparison	Estimate	Std.Error	t.Value	p.Value
gag vs. env	0.9405822	0.5129923	5.38391	1.20E-07
B vs. C	0.8396171	0.6310992	2.62301	9.30E-03
Other* vs. B	0.1350254	0.6555201	-2.8332	5.30E-03
Other* vs C	0.4497069	0.6310992	-0.3198	0.75

B. Proportion of genes that were intact (Wilcoxon tests)

		Time Window 1#			Time Window 2		
Comparison	Subset	Medians	P-Value	FDR Q	Medians	P-Value	FDR Q
gag vs. env^	Clade B	98% vs. 93%	2.90E-11	1.74E-10	98% vs. 91%	4.70E-10	1.41E-09
gag vs. env^	Clade C	92% vs 82%	1.14E-13	2.05E-12	88% vs. 79%	5.80E-11	2.61E-10
gag vs. env^	Other*	97% vs. 83%	2.90E-11	1.74E-10	98% vs. 90%	1.90E-06	3.11E-06
C vs. B	gag	92% vs. 98%	8.64E-07	1.56E-06	88% vs. 98%	2.74E-10	9.86E-10
B vs. Other*	gag	98% vs. 97%	0.006	7.20E-03	98% vs. 98%	0.546	0.546
C vs. Other*	gag	92% vs. 97%	0.0003	3.86E-04	88% vs. 98%	1.51E-08	3.02E-08
C vs. B	env	82% vs. 93%	7.11E-09	1.60E-08	79% vs. 91%	3.50E-09	9.00E-09
B vs. Other*	env	93% vs. 83%	2.19E-05	3.29E-05	91% vs. 90%	0.163	0.173
C vs. Other*	env	82% vs. 83%	0.141	0.159	79% vs. 90%	5.08E-05	7.03E-05

C. Proportion of sequences that were hypermutated (Wilcoxon tests)

		Time Window 1			Time Window 2		
Comparison	Subset	Medians	P-Value	FDR Q	Medians	P-Value	FDR Q
gag vs. env	Clade B	0% vs. 0.78%	0.665	0.856	0.34% VS. 1.6%	0.223	0.574
gag vs. env	Clade C	0.22% vs. 0.17%	0.620	0.856	0.21% VS. 0%	0.075	0.345
gag vs. env	Other*	0.65% vs. 0.64%	0.124	0.372	0% VS. 0%	0.295	0.589
C vs. B	gag	0.22% vs. 0%	0.839	0.899	0.21% VS. 0.34%	0.894	0.899
B vs. Other*	gag	0% vs. 0.65%	0.096	0.345	0.34% VS. 0%	0.866	0.899
C vs. Other*	gag	0.22% vs. 0.65%	0.078	0.345	0.21% VS. 0%	0.899	0.899
C vs. B	env	0.17% vs. 0.78%	0.288	0.589	0% VS. 1.6%	0.001	0.012
B vs. Other*	env	0.78% vs. 0.64%	0.619	0.856	0% VS. 1.6%	0.025	0.223
C vs. Other*	env	0.17% vs. 0.64%	0.492	0.804	0% VS. 0%	0.369	0.665

* "Other" refers to a combined dataset including all intersubtype recombinants and pure subtype sequences aside from subtype B and C. * Two time windows were examined, see Online Methods. ^ Normalized by gene length when comparing different gene regions. Red font indicates significant comparisons by both p-value <0.05 and FDR Q<0.2.

Differences between genes and time points in the frequency of intact genes were assessed using both a parametric method, which allowed us to combine all variable into a single model, and a non-parametric method, by running a 2-wayWilcoxon test comparing each variable within each subgroup (gene/time point/subtype) and then applying an FDR threshold of 0.2 to correct for multiple testing (Supplemental Table 4). For the parametric method, we ran a random effects generalized linear model (GLM) with the logit transformed frequency as dependent variable, gene (either *gag* or *env*) and subtype (categorized as C, B or "other" for all other subtypes and including all intersubtype recombinants) as independent variables, and participant ID as a random effect. To make days since COB comparable across cohorts, we chose times since COB/EDDI to be as uniform as possible across participants, with each study participant represented only once for each model run. As such, we identified two time points ("windows") with mean times since COB/EDDI of 30 and 45 days, respectively, selected as follows: 1) Sequence datasets from approximately 30 days post COB/EDDI, including all first time point samples from V703 and V704, the second time point for FRESH, and the third time point for RV217, as for both the FRESH and RV217 the first time point was on average sampled around two weeks earlier than the first time point in the AMP cohorts (Supplementary Table 3). 2) Sequence datasets from approximately 45 days post COB/EDDI, including the second time point for V703 and V704, the third for FRESH and the fourth for RV217. Associations between viral loads and fraction of intact sequences and hypermutated sequences were assessed using the Kendall correlation coefficient and test. These analyses were computed in R (version 4.2.1) using packages lme4, LaplacesDemon, and pbkrtest. Confidence intervals were calculated using the binomial (Wilson score interval) method. The power to detect minor variants by frequency and number of sampled sequences was calculated using the binomial function (pbinom) in R Studio.

Supplementary Table 5. Codon usage related to stop codon formation

A. Stop Codon	Codons that could change to a stop with a single point mutation (Amino acid)	Relative usage to encode the stated amino acid	
		Subtype B	Subtype C
TAA	TTA (L)	24	18
	AAA (K)	65	62
	GAA (E)	73	72
TAG	TTG (L)	20	21
	CAG (Q)	49	46
	AAG (K)	35	38
	GAG (E)	27	28
TGA	TGT (C)	72	67
	TGC (C)	28	33
	CGA (R)	1	4
	AGA (R)	62	62
	Proportion of total	23.50%	22.70%

**B.** TGG (W) proportion of all codons		3.37%	3.22%

Supplementary Table 6. Comparison of observed versus expected mutations distinguishing lineages

	GP										REN									
	Detection of 2nd lineage – Days post	Number of sequences		Detected at	Sequences	Mismatch	Average number of distinguishing mutations	Observed distinguishing point mutations	Fold difference (observed/	Observed number of distinguishing	Detection of 2nd lineage – Days post	Number of sequences		Detected at	Sequences	Mismatch	Average number of distinguishing mutations	Observed distinguishing point mutations	Fold difference (observed/	Observed number of distinguishing
PtID	COB	MxL1	MxL2	later TP	compared[1]	rate (%)	expected[2]	(99% CI)	expected)	indels	COB	MxL1	MxL2	later TP	compared[1]	rate (%)	expected[1]	(99% CI)	expected)	indels
**A. MLA detected in GP and REN from the FRESH and RV217 cohorts**																				
**FRESH**																				
79	19	208	8	yes	003_1_80, 003_212_1	0.974	1.8	26 (15, 42)	14.1x	1	19	22	3	yes	003_1_7, 003_17_1	7.379	2.2	223 (186, 264)	100.9x	8
186	5	145	2	yes	001_1_62, 001_6_1	2.656	0.9	72 (52, 97)	84.7x	0	5	330	1	yes	001_1_160, 001_164_1	8.41	1.0	253 (214, 297)	248x	2
267	9	617	39	yes	002_1_182, 002_4_6	1.275	1.1	34 (21, 52)	30x	3	9	335	11	yes	002_1_124, 002_4_6	2.454	1.4	74 (54, 99)	54.4x	4
271	44	217	19	yes	004_1_114, 007_1_8	1.437	3.6	39 (25, 58)	10.8x	0	44	38	7	yes	004_1_4, 004_35_1	1.715	4.3	53 (36, 75)	12.2x	4
271[1]	44	67	19	yes	007_2_6, 007_1_8	1.437	3.1	39 (25, 58)	10.8x	0										
318	16	130	1 + 11 recs	yes	001_1_77, 003_75_1	0.482	1.6	13 (6, 26)	8x	0	3	21	2	yes	001_1_11, 001_5_1	0.832	0.9	25 (14, 93)	29.4x	2
318	16	314	1 + 11 recs	yes	003_1_134, 003_75_1	0.482	1.6	13 (6, 26)	8x	0										
**RV217**																				
20337	4	112	20	yes	01_1_85, 01_5_3	0.836	0.8	21 (11, 36)	27x	0	4	63	15	yes			0.9	69 (50, 93)	73.8x	5
20502	4	59	35	recs only	01_1_26, 01_2_20	1.043	0.8	26 (15, 42)	33.4x	0	4	19	9	recs only	01_1_6, 01_2_2	2.233	0.9	72 (52, 97)	77x	2
40061	5	26	1	yes	02_1_12, 02_5_1	1.503	0.9	37 (23, 56)	43.5x	0	5	332	1	yes	02_1_26, 02_8_1	2.552	1.0	77 (56, 103)	75.5X	2
40100	5	164	6	yes	01_1_106, 01_2_4	1.51	0.9	38 (24, 57)	44.7x	0	5	386	11	yes	01_1_177, 01_2_7	1.745	1.0	56 (39, 78)	54.9x	3
40123	7	24	6	yes	02_1_17, 02_2_5	1.035	1.0	26 (15, 42)	26.2x	1	17	221	98	yes	05_1_89, 05_2_43	0.689	2.0	21 (11, 36)	10.3x	3
40231	3	236	2	yes	01_1_183, 01_7_2	0.398	0.7	10 (4, 21)	14.1x	0	10	281	4	yes	03_1_116, 03_4_2	0.528	1.4	17 (8, 31)	11.8x	1
											10	67	4	yes	01_1_39, 03_4_2	0.528	1.4	17 (8, 31)	11.8x	1
40250	9	125	2	yes	03_1_51, 03_25_1	0.318	1.1	8 (3, 19)	7.1x	1	9	77	1	yes	03_1_30, 03_10_1	0.656	1.4	20 (10, 35)	14.7x	0
40265[3]	6	204	4	yes	02_1_104, 02_4_3	0.684	0.9	17 (8, 31)	18.5x	1	6	93	2	yes	02_1_27, 02_33_1	0.668	1.1	21 (11, 36)	19x	0
40363	4	7	2	yes	01_1_5, 01_3_4	0.279	0.8	8 (3, 19)	10.3x	0	4	10	8	yes	01_1_7, 01_3_2	1.078	0.9	33 (20, 51)	35.3x	4
40436	19	34	1	yes	01_1_25, 06_107_1	0.8014	1.8	20 (10, 35)	10.9x	0	19	161	2	yes	06_1_51, 06_9_1	1.2314	2.2	39 (25, 58)	17.6x	2
40436	19	192	1	yes	06_01_81, 06_107_1	0.7994	1.8	20 (10, 35)	10.9x	0	19	38	2	yes	01_1_18, 06_9_1	1.2994	2.2	40 (25, 58)	17.6x	2

Supplementary Table 6 (continued). Comparison of observed versus expected mutations distinguishing lineages

PtID	GP: Detection of 2nd lineage Days post COB	GP: Number of sequences MxL1	GP: Number of sequences MxL2	GP: Detected at later TP	GP: Sequences compared[1]	GP: Mismatch rate (%)	GP: Average number of distinguishing mutations expected[2]	GP: Observed distinguishing point mutations (99% CI)	GP: Fold difference (observed/ expected)	GP: Observed number of distinguishing indels	REN: Detection of 2nd lineage Days post COB	REN: Number of sequences MxL1	REN: Number of sequences MxL2	REN: Detected at later TP	REN: Sequences compared[1]	REN: Mismatch rate (%)	REN: Average number of distinguishing mutations expected[1]	REN: Observed distinguishing point mutations (99% CI)	REN: Fold difference (observed/ expected)	REN: Observed number of distinguishing indels
**B. Only one sequence initially detected in the MxL2 lineage in both GP and REN**																				
**HVTN 703**																				
13	18	206	1	yes	090_1_85, 090_7_1	1.362	1.8	37 (23, 56)	20.9x	0	18	110	1	yes	090_1_38, 090_73_1	1.538	2.1	47 (31, 68)	22.1x	1
1313	33	38	1	recs only	040_1_17, 040_3_1	0.294	2.83	8 (2.6, 19)	2.8x	0	33	144	1	rec only	040_1_61, 040_77_1	1.845	3.4	56 (39, 78)	16.5x	0
**HVTN 704**																				
3008	23	106	1	yes	040_1_65, 040_40_1	1.432	2.1	36 (22, 55)	16.9x	0	23	127	1	yes	040_1_78, 050_4_1	5.792	2.6	174 (142, 211)	68.2x	0
											23	137	1	yes	050_1_65, 050_4_1	5.792	2.6	174 (142, 211)	68.2x	0
**C. Fewer than 15 changes distinguishing lineages in GP and/or REN**																				
**HVTN703**																				
566	18	70	9	yes	160_1_31, 160_2_2	0.373	1.8	10 (94, 21)	5.6x	0	18	246	29	yes	160_1_100, 160_2_15	0.296	2.1	9 (3, 20)	4.2x	1
**D. MLA detected in only GP or REN**																				
**HVTN 703**																				
1764_GP	44	123	1 + 4 recs	recs only	250_1_48, 250_79_1	0.331	3.6	9 (3.1, 20)[5]	2.5x	0										
1889_GP	32	29	1	no	100_1_18, 100_13_1	1.48	2.8	40 (26, 59)	14.5x	0										
**HVTN 704**																				
279_GP	8	32	1	no	080_1_29, 080_3_1	0.609	1.1	15 (6.9, 28)	14.1x	1										
726_GP	31	226	12	yes	080_1_131, 080_2_7	0.198	2.7	5 (1, 14)[6]	1.9x	0										
1109_REN	34	291	1	yes	140_1_124, 140_140_1	1.796	3.5	55 (38, 77)	15.8x	3										

Notes:

1 Prototype sequences from each lineage were selected based on being closest to the phylogenetic root, and typically, the most often detected at the first time point the 2nd lineage was detected. Sequence code: Visit_CollapsedSequenceID_SequenceFrequency. Yellow highlighted cells indicate samples in which the expected number of mutations is within the 99% CI of the observed mutations. Data in red font correspond to comparisons of the MxL2 lineage to the prototype MxL1 lineage from the same time point.

2 Only point mutations are counted. GP equation: (Days COB+7)*(3.4 x 10e-5 mutations/site*cycle)*(cycle/1.2 days)*(2500 sites) = x mutations expected. REN equation: (Days COB+7)*(3.4 x 10e-5 mutations/site*cycle)*(cycle/1.2 days)*(3000 sites) = x mutations expected. Derivation assumptions: Days of infection prior to detection of RNA = 7 (from Rolland et al. 2020), Replication cycle time = 1.2 days (minimum estimate from Perelson et al. 1996), Mutations/site each cycle = 3.4 x 10e-5 (from Mansky & Temin 1995), 2500 sites in GP, 3000 sites in REN.

3 Only 4 sequences were obtained from visit 01, so this comparison was carried out using sequences from visit 02

4 Mismatch rate differences due to amplicon length differences

5 Observed number of mutations below 97% CI of expectation

6 Observed number of mutations only below 70% CI of expectation and was eliminated as an MLA

Supplementary Table 7. Selection within Gag and Env at different stages of Acute and Early infection[1]

Protein	Stage	Number of Sites	α>β (negative)[2]	α<β (positive)[2]	Ratio (Neg/Pos.)
Gag	Overall	238,779	2026 (0.85%)	290 (0.12%)	7.1
	i	70,338	342 (0.49%)	52 (0.07%)	7.0
	ii	92,550	867 (0.94%)	103 (0.11%)	8.5
	iii	54,296	579 (1.07%)	76 (0.14%)	7.6
	iv	21,595	238 (1.10%)	59 (0.27%)	
Env	Overall	481,100	1249 (0.26%)	801 (0.17%)	1.5
	i	158,480	276 (0.17%)	137 (0.09%)	1.9
	ii	186,780	497 (0.27%)	288 (0.15%)	1.8
	iii	101,880	346 (0.34%)	286 (0.28%)	1.2
	iv	33,960	130 (0.38%)	90 (0.27%)	

[1] Only lineages with at least 10 members were used in these calculations. [2] Sites with probabilities $\geq$0.9 (percent of total).

**Supplementary Table 8. Acute and Early Infection Staging**	
Infection Stage definitions	Stage
Ascending viremia; 1-18 days from the COB or EDDI[1]; Fiebig I-III[2]	i
Rapidly descending viremia; 19-40 days from the COB or EDDI; Fiebig IV-V	ii
Slowly descending viremia; 41-61 days from the COB or EDDI; Fiebig V	iii
Entering steady state viremia; > 61 days from the COB or EDDI; Fiebig V	iv

[1] COB = Center of bounds between the last negative and first positive HIV RNA test, used to date virus acquisitions for the RV217 and FRESH cohorts. EDDI = Estimated date of detectable infection assigned using COB and immune response data (Rossenkhan R, et al, mBio. 2025:e0188125). [2] Fiebig stages of infection (Fiebig EW, et al. AIDS. 2003;17(13):1871–9).

**Supplementary Table 9. Median gp120 Env and variable region lengths by subtype**						
Dataset:<br>Subtype:	LANL<br>B	RV217/<br>HVTN 704<br>B	LANL<br>C	RV217/FRESH<br>/HVTN 703<br>C	LANL<br>CRF01AE	RV217<br>CRF01AE
V1	31	30	28	27	31	31
V2	42	42	44	43	43	45
V4	32	33	29	28	28	29
V5	13	14	13	13	12	12
V regions total	118	119	114	111	114	117
gp120	512	514	505	506	507	511

Supplementary Fig. 1

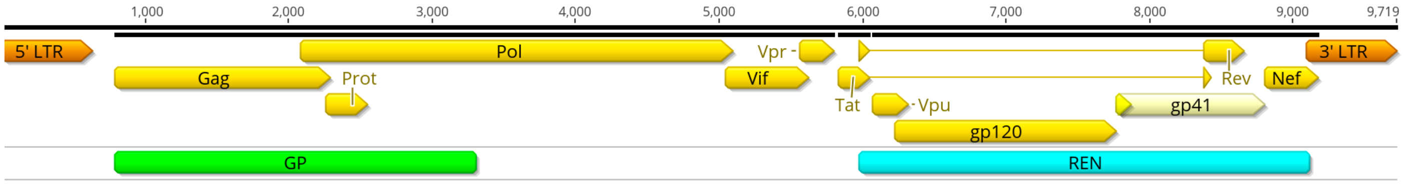

S1 Fig. HIV-1 genome representation corresponding to the reference sequence HXB2 (GenBank accession number K03455.1) with amplicons used in this study shown below the open reading frame map. The GP amplicon extends from HXB2 position 790 to 3297 and the REN amplicon from HXB2 position 5970 to 9012. Image generated in Geneious Prime version 2025.2.1 (Dotmatics).

Supplementary Fig. 2

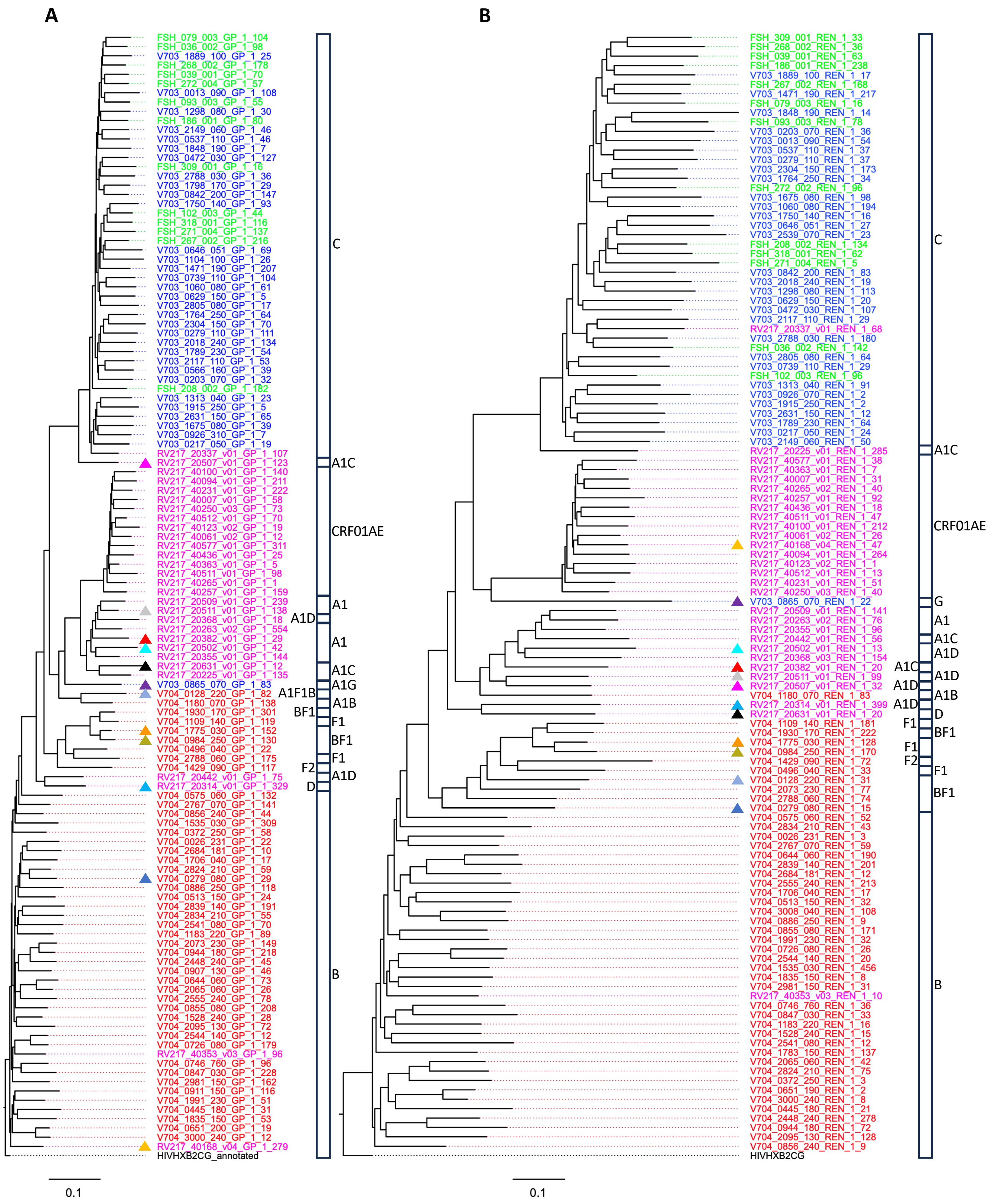

Supplementary Figure 2. Phylogenetic analysis of representative HIV viral genome sequences used in this study. The most common sequence detected in the GP (**A**) and REN (**B**) region from each participant were aligned and maximum likelihood trees generated. Sequence labels were colored by cohort (FRESH, green; HVTN 703, blue; RV217, magenta; HVTN 704, red), with the Subtype B HXB2 reference sequence in black. The identity of subtypes and intersubtype recombinant designations are indicated adjacent to the sectors within the vertical bars. Viruses with discordant subtypes in GP versus REN are indicated with colored triangles to the left of the sequence labels. The scales at the bottom of each tree indicate 10% sequence divergence.

Supplementary Fig. 3

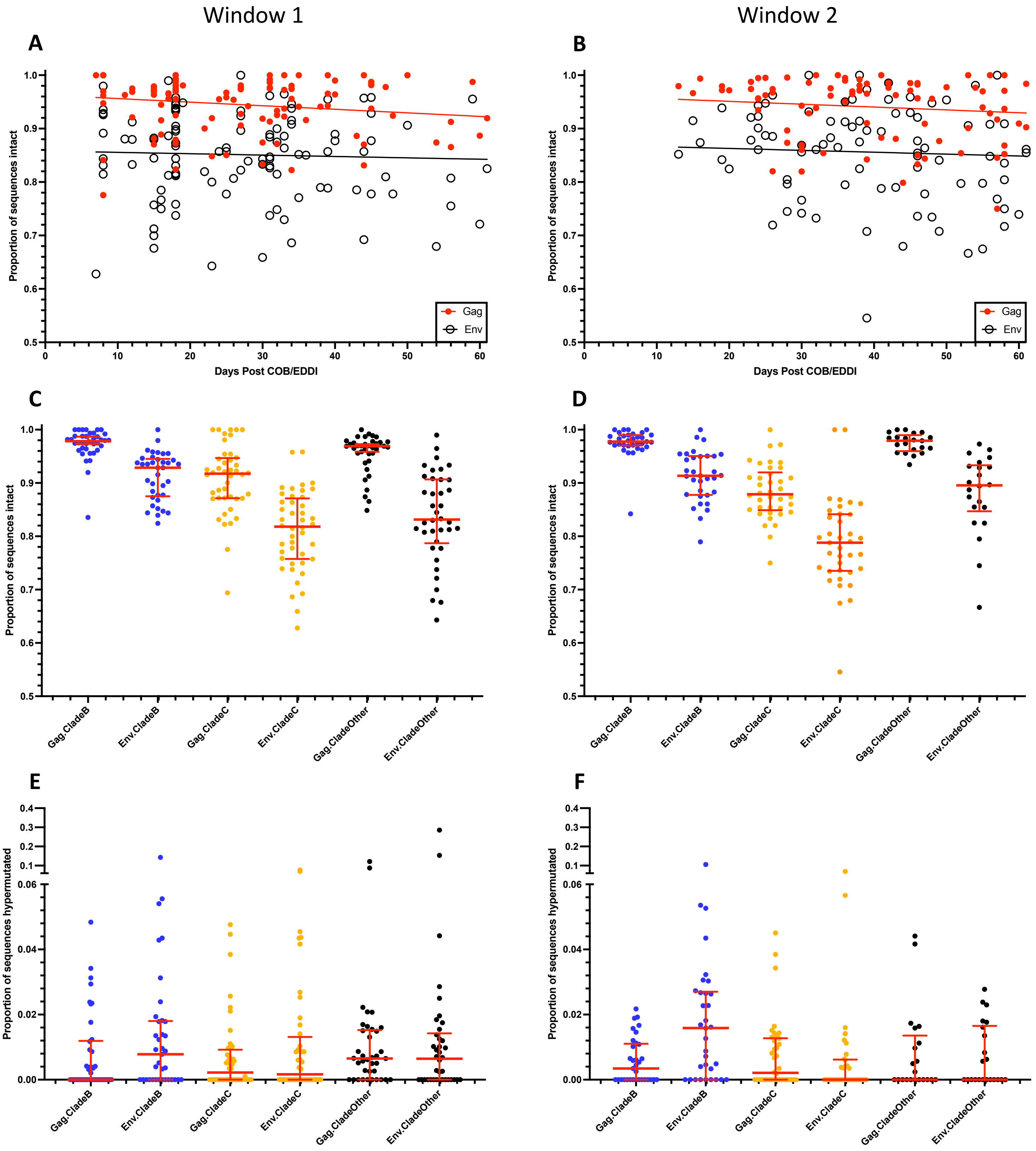

Supplementary Fig 3. Intact and hypermutated *gag* and *env* genes as a function of time and viral subtype. The proportion of intact *gag* and *env* genes (i.e., lacking putative inactivating mutations, S1 Table) as a function of days post COB (RV217 and FRESH cohorts) or EDDI (HVTN 703 and HVTN 704 cohorts) are shown for the first (**A**) and second (**B**) time windows. Red symbols refer to *gag* genes and open black symbols refer to *env* genes. The proportion of intact genes as a function of viral subtype are shown in panels (**C**) and (**D**) in windows 1 and 2, respectively. Panels (**E**) and (**F**) show proportion of *gag* and *env* genes that were hypermutated (25) in windows 1 and 2, respectively.

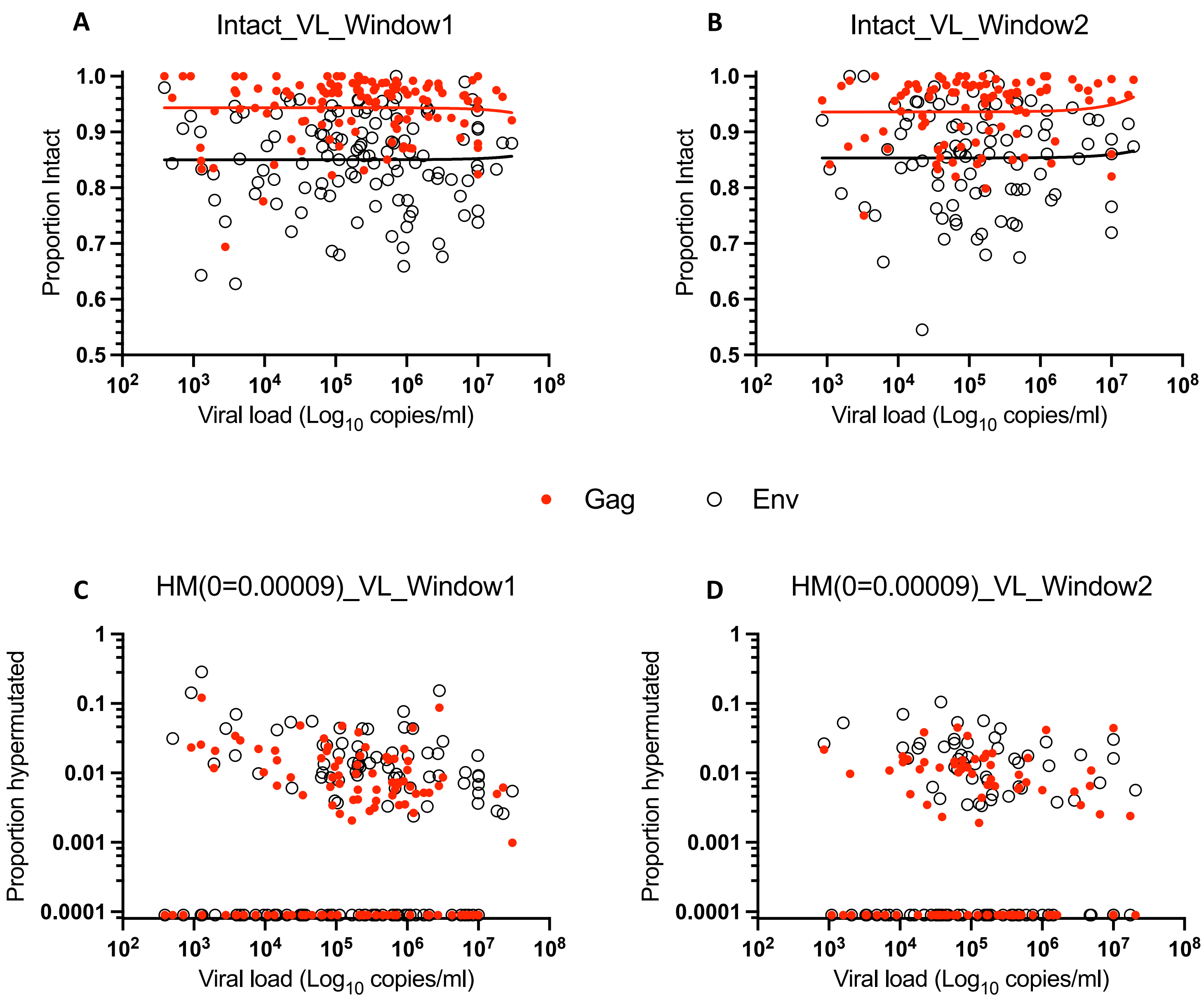

Supplementary Fig. 4. Viral load correlated with intact genes and hypermutation. Proportion of intact genes as a function of viral load in the first (**A**) and second (**B**) time windows. Proportion of sequences that were hypermutated as a function of plasma viral load for time windows 1 (**C**) and 2 (**D**). For illustration on a log plot, those datasets with no hypermutated sequences were set to a value of 0.00009. Gag gene values are shown with red dots and Env genes with open black circles.

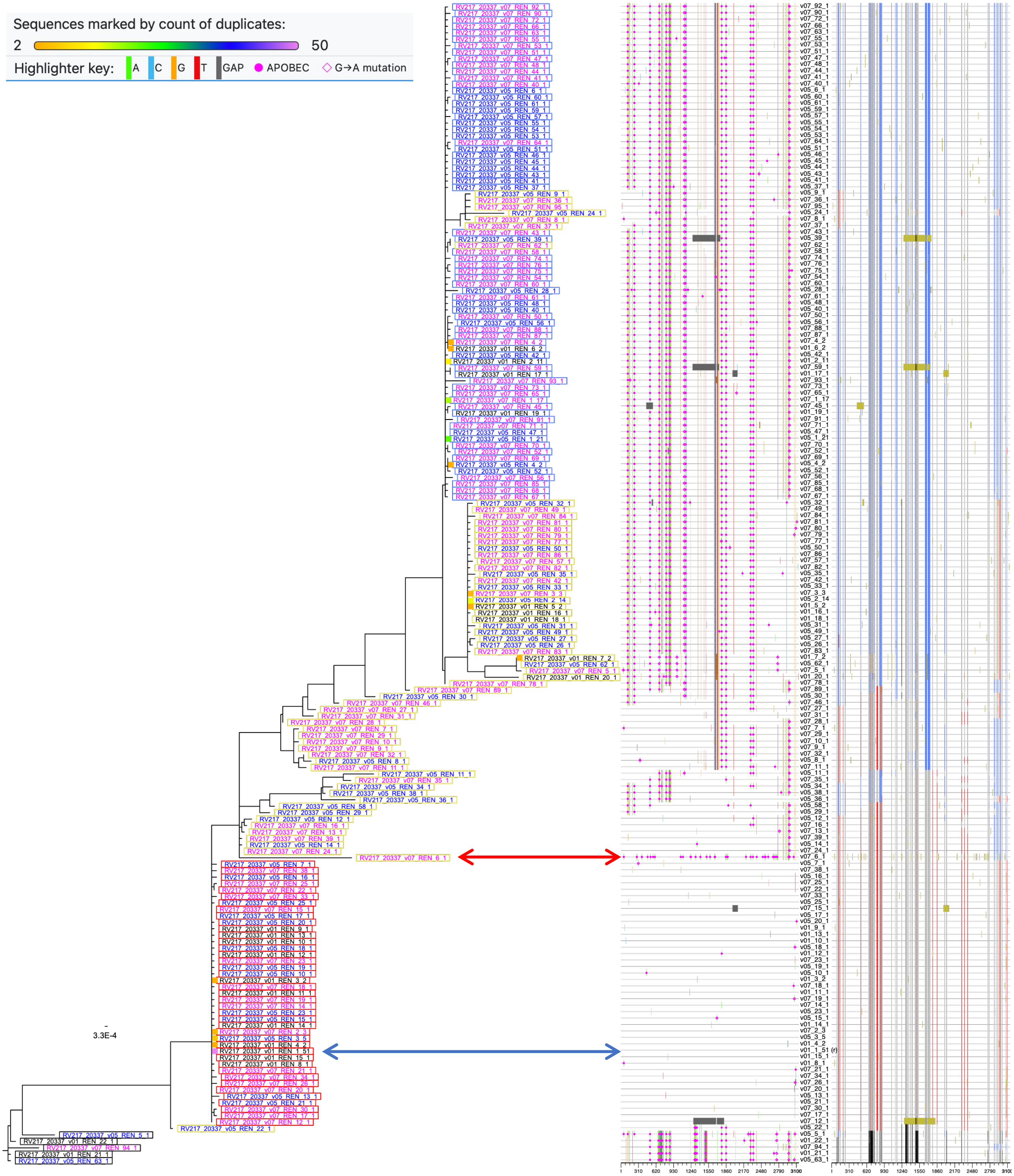

Supplementary Fig. 5. Phylogeny-based lineage definition method. Phylobook (Furlong, J.C., et al. *Biotechniques* **76**, 263–274 (2024) output from one RV217 participant. **Left panel:** Identical sequences were collapsed into one, with the number of collapsed sequences roughly indicated by a colored box to the left of the taxon name (see color key at top) and the precise number shown at the end of the taxon name. Taxon names were color coded based on visit (1st, black; 2nd, blue; 3rd, magenta). Taxon names were enclosed with colored boxes corresponding to different lineages: most common at the 1st time point, red; 2nd most common, blue; 3rd, black; recombinants, yellow). **Middle panel:** Highlighter plot showing differences relative to the most common sequence at the 1st timepoint (blue arrow). Dark gray areas show the position of deletions. The red arrow indicates a hypermutated sequence. **Right panel:** Match plot showing identities to each of the 3 identified lineages (gray if found in multiple lineages).

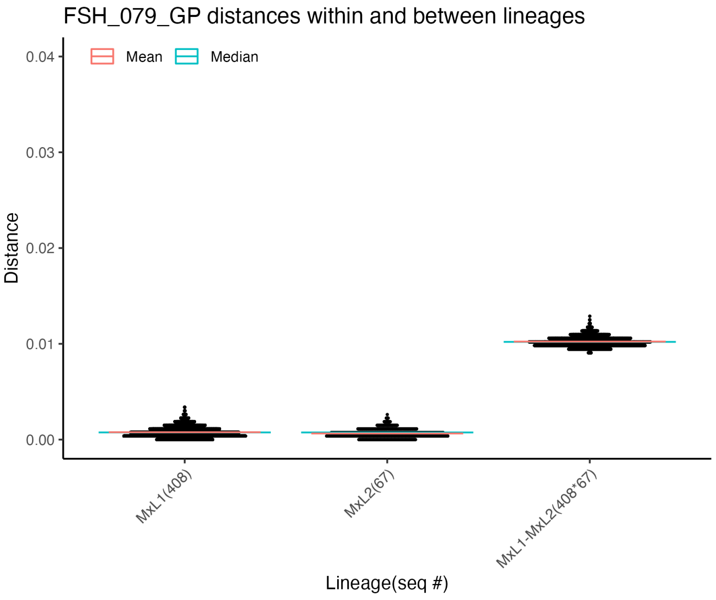

Supplementary Figure 6. Maximum likelihood pairwise differences within participants infected with multiple lineages. Pairwise differences were determined for all time points combined. Genetic distance distributions are shown for individual lineages as well as for inter-lineage comparisons. The numbers in parentheses below each distribution indicate the number of sequences within that lineage. In some cases (data points circled in red), deletions within variable regions resulted in lower pairwise distances. MxL(n) refers to the lineages observed within multilineage infections, numbered in order of abundance at the first time point sampled. “x” refers to the stage of infection, not specified here since all timepoints were pooled for these analyses.

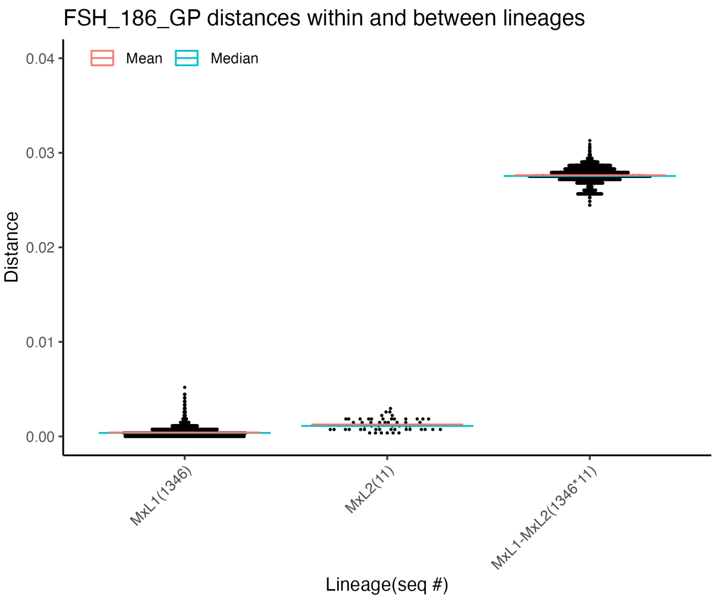

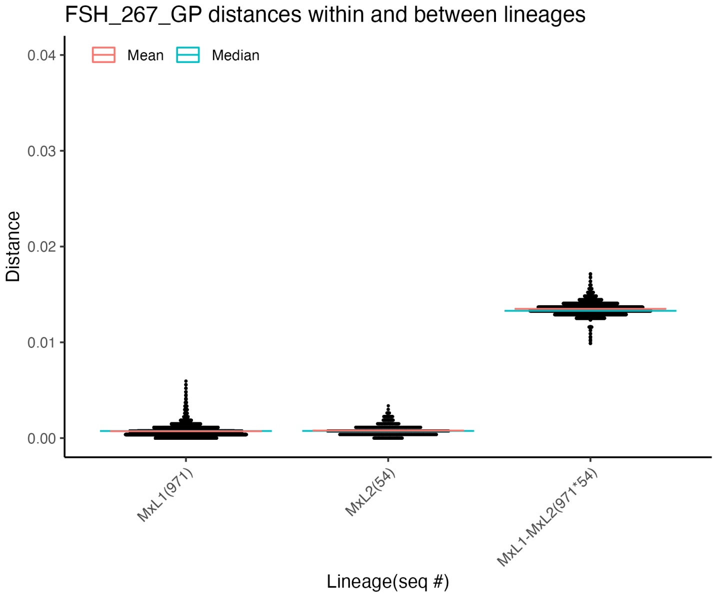

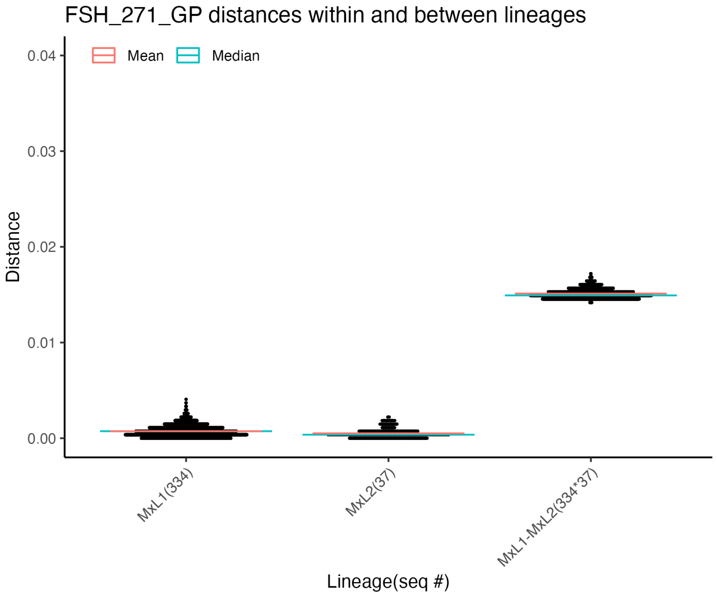

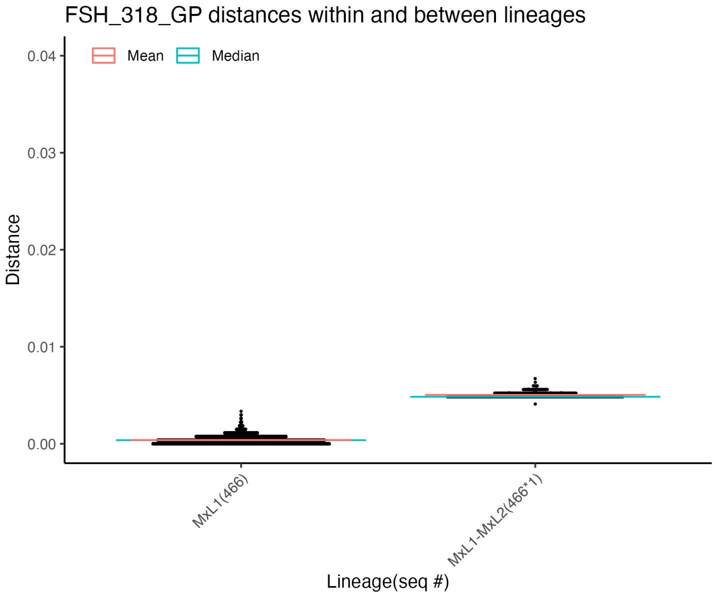

S3.2 Fig.

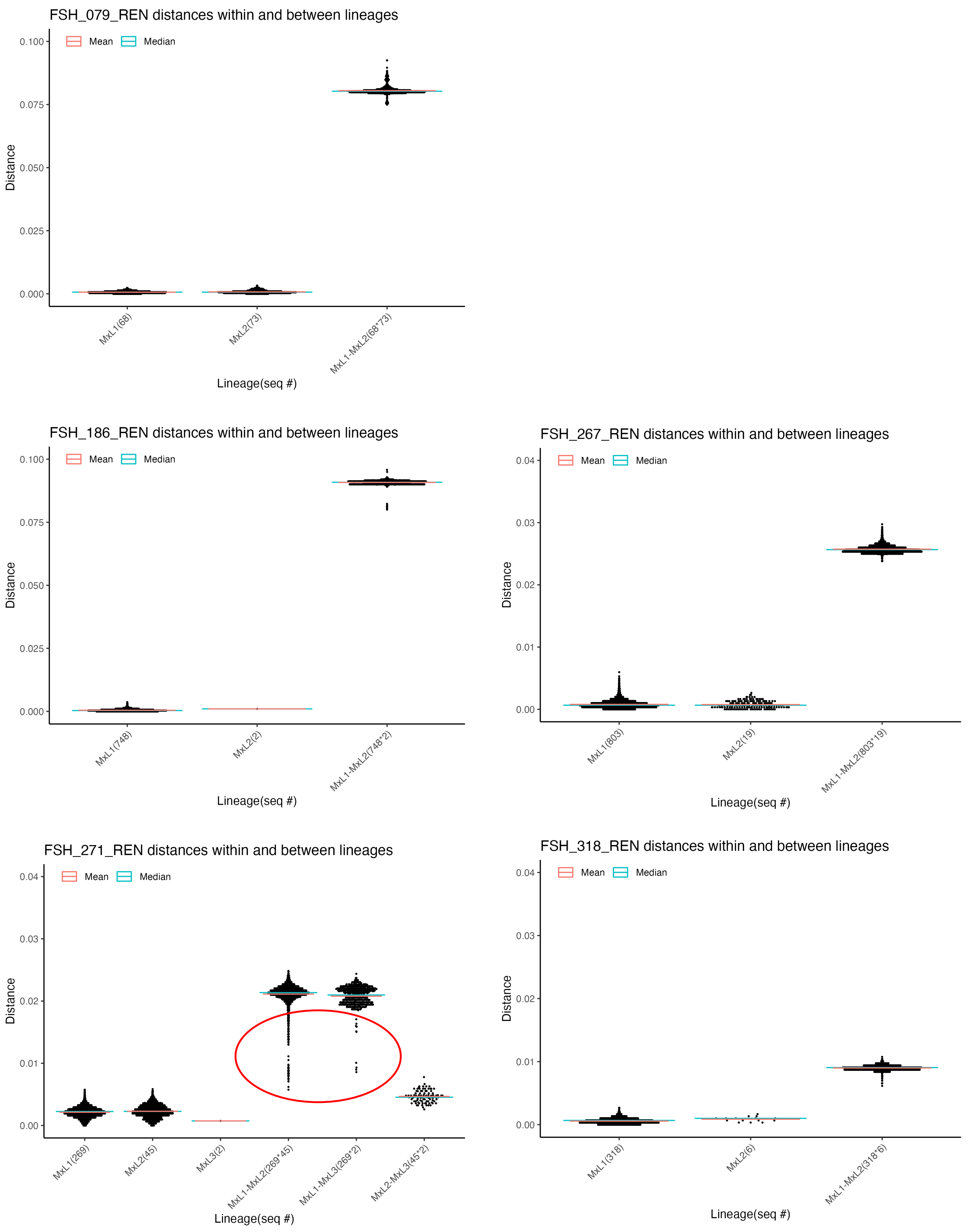
FSH_079_REN distances within and between lineages
Mean
Median
Distance
0.100
0.075
0.050
0.025
0.000
MxL1(68)
MxL2(73)
MxL1-MxL2(68*73)
Lineage(seq #)
FSH_186_REN distances within and between lineages
Mean
Median
Distance
0.100
0.075
0.050
0.025
0.000
MxL1(748)
MxL2(2)
MxL1-MxL2(748*2)
Lineage(seq #)
FSH_267_REN distances within and between lineages
Mean
Median
Distance
0.04
0.03
0.02
0.01
0.00
MxL1(803)
MxL2(19)
MxL1-MxL2(803*19)
Lineage(seq #)
FSH_271_REN distances within and between lineages
Mean
Median
Distance
0.04
0.03
0.02
0.01
0.00
MxL1(269)
MxL2(45)
MxL3(2)
MxL1-MxL2(269*45)
MxL1-MxL3(269*2)
MxL2-MxL3(45*2)
Lineage(seq #)
FSH_318_REN distances within and between lineages
Mean
Median
Distance
0.04
0.03
0.02
0.01
0.00
MxL1(318)
MxL2(6)
MxL1-MxL2(318*6)
Lineage(seq #)

S6.3 Fig.

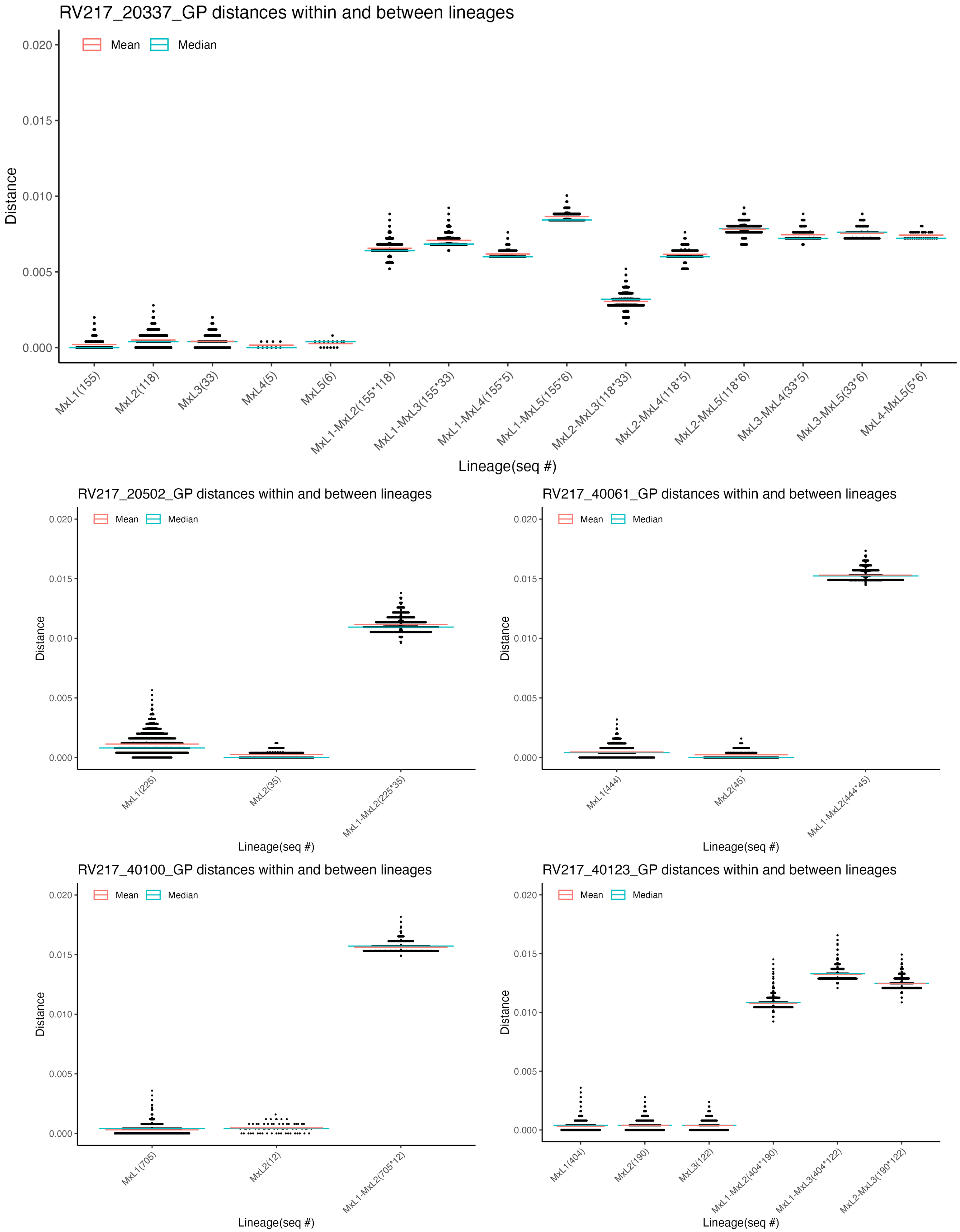
RV217_20337_GP distances within and between lineages
Mean
Median
Distance
0.020
0.015
0.010
0.005
0.000
MxL1(155)
MxL2(118)
MxL3(33)
MxL4(5)
MxL5(6)
MxL1-MxL2(155*118)
MxL1-MxL3(155*33)
MxL1-MxL4(155*5)
MxL1-MxL5(155*6)
MxL2-MxL3(118*33)
MxL2-MxL4(118*5)
MxL2-MxL5(118*6)
MxL3-MxL4(33*5)
MxL3-MxL5(33*6)
MxL4-MxL5(5*6)
Lineage(seq #)
RV217_20502_GP distances within and between lineages
Mean
Median
Distance
MxL1(225)
MxL2(35)
MxL1-MxL2(225*35)
Lineage(seq #)
RV217_40061_GP distances within and between lineages
Mean
Median
Distance
MxL1(444)
MxL2(45)
MxL1-MxL2(444*45)
Lineage(seq #)
RV217_40100_GP distances within and between lineages
Mean
Median
Distance
MxL1(705)
MxL2(12)
MxL1-MxL2(705*12)
Lineage(seq #)
RV217_40123_GP distances within and between lineages
Mean
Median
Distance
MxL1(404)
MxL2(190)
MxL3(122)
MxL1-MxL2(404*190)
MxL1-MxL3(404*122)
MxL2-MxL3(190*122)
Lineage(seq #)

S6.4 Fig.

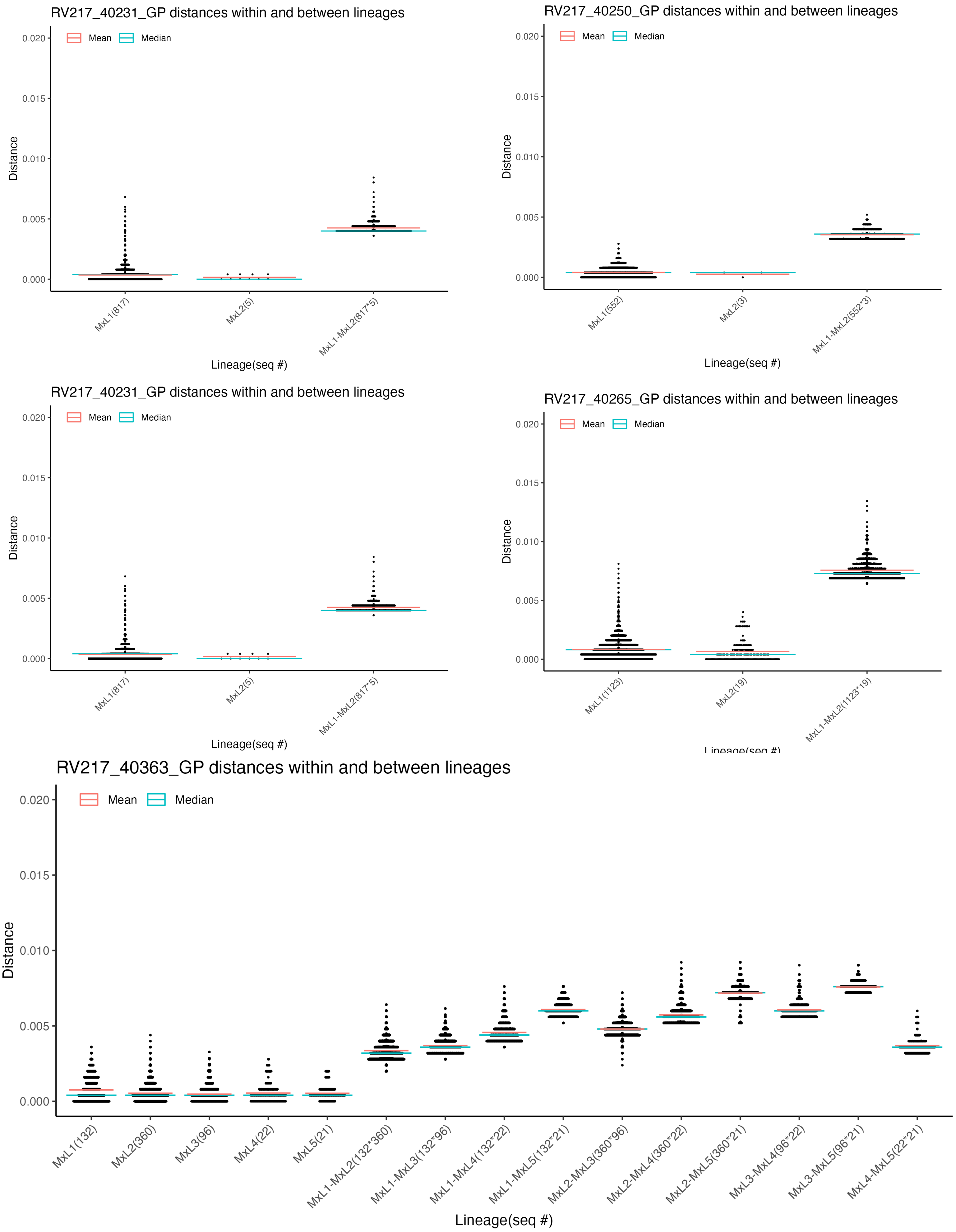
RV217_40231_GP distances within and between lineages
Mean
Median
Distance
0.020
0.015
0.010
0.005
0.000
MxL1(817)
MxL2(5)
MxL1-MxL2(817*5)
Lineage(seq #)
RV217_40250_GP distances within and between lineages
Mean
Median
Distance
0.020
0.015
0.010
0.005
0.000
MxL1(552)
MxL2(3)
MxL1-MxL2(552*3)
Lineage(seq #)
RV217_40231_GP distances within and between lineages
Mean
Median
Distance
0.020
0.015
0.010
0.005
0.000
MxL1(817)
MxL2(5)
MxL1-MxL2(817*5)
Lineage(seq #)
RV217_40265_GP distances within and between lineages
Mean
Median
Distance
0.020
0.015
0.010
0.005
0.000
MxL1(1123)
MxL2(19)
MxL1-MxL2(1123*19)
RV217_40363_GP distances within and between lineages
Mean
Median
Distance
0.020
0.015
0.010
0.005
0.000
MxL1(132)
MxL2(360)
MxL3(96)
MxL4(22)
MxL5(21)
MxL1-MxL2(132*360)
MxL1-MxL3(132*96)
MxL1-MxL4(132*22)
MxL1-MxL5(132*21)
MxL2-MxL3(360*96)
MxL2-MxL4(360*22)
MxL2-MxL5(360*21)
MxL3-MxL4(96*22)
MxL3-MxL5(96*21)
MxL4-MxL5(22*21)
Lineage(seq #)

S6.5 Fig.

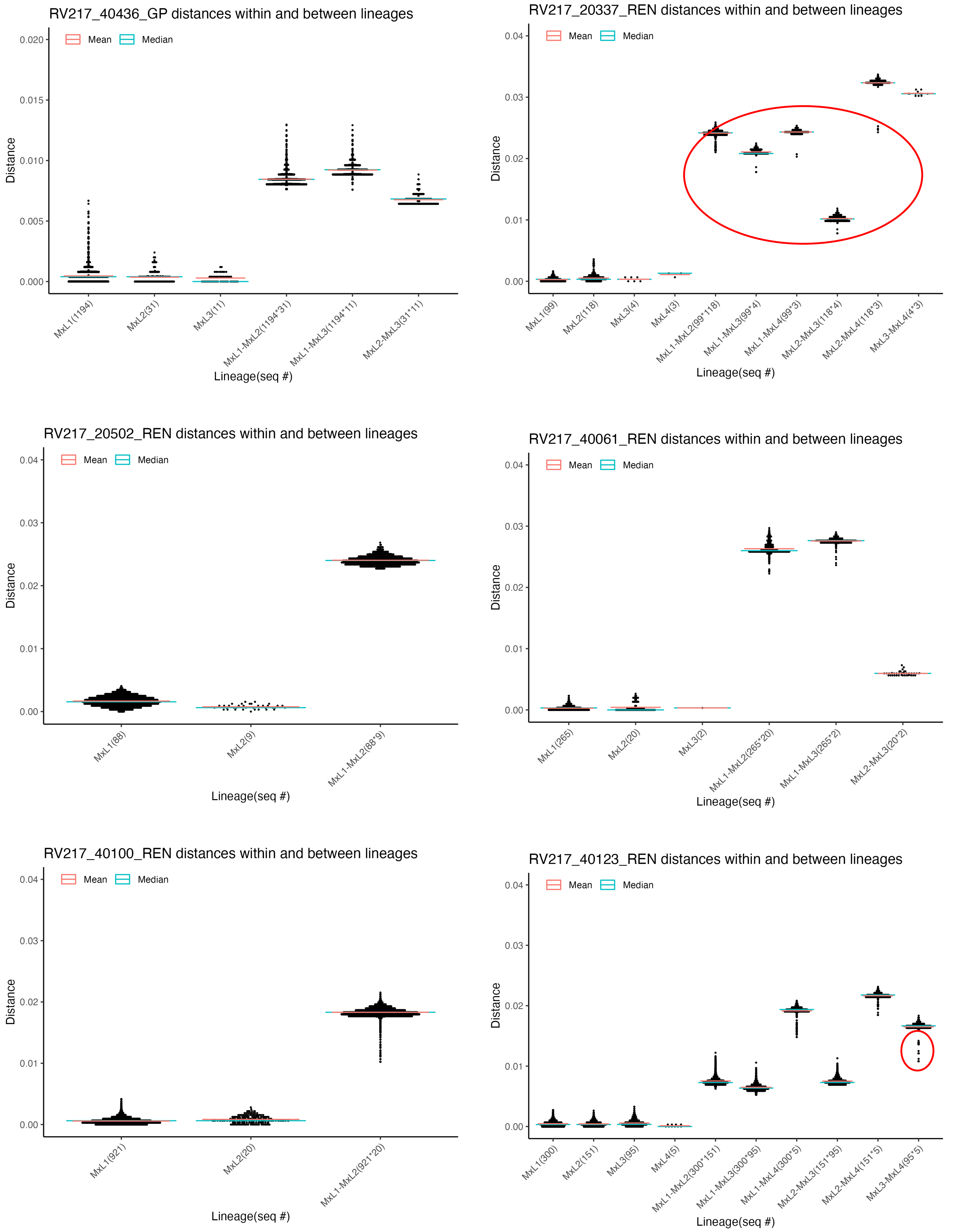
RV217_40436_GP distances within and between lineages
Mean
Median
Distance
0.020
0.015
0.010
0.005
0.000
MxL1(1194)
MxL2(31)
MxL3(11)
MxL1-MxL2(1194*31)
MxL1-MxL3(1194*11)
MxL2-MxL3(31*11)
Lineage(seq #)
RV217_20337_REN distances within and between lineages
Mean
Median
Distance
0.04
0.03
0.02
0.01
0.00
MxL1(99)
MxL2(118)
MxL3(4)
MxL4(3)
MxL1-MxL2(99*118)
MxL1-MxL3(99*4)
MxL1-MxL4(99*3)
MxL2-MxL3(118*4)
MxL2-MxL4(118*3)
MxL3-MxL4(4*3)
Lineage(seq #)
RV217_20502_REN distances within and between lineages
Mean
Median
Distance
0.04
0.03
0.02
0.01
0.00
MxL1(88)
MxL2(9)
MxL1-MxL2(88*9)
Lineage(seq #)
RV217_40061_REN distances within and between lineages
Mean
Median
Distance
0.04
0.03
0.02
0.01
0.00
MxL1(265)
MxL2(20)
MxL3(2)
MxL1-MxL2(265*20)
MxL1-MxL3(265*2)
MxL2-MxL3(20*2)
Lineage(seq #)
RV217_40100_REN distances within and between lineages
Mean
Median
Distance
0.04
0.03
0.02
0.01
0.00
MxL1(921)
MxL2(20)
MxL1-MxL2(921*20)
Lineage(seq #)
RV217_40123_REN distances within and between lineages
Mean
Median
Distance
0.04
0.03
0.02
0.01
0.00
MxL1(300)
MxL2(151)
MxL3(95)
MxL4(5)
MxL1-MxL2(300*151)
MxL1-MxL3(300*95)
MxL1-MxL4(300*5)
MxL2-MxL3(151*95)
MxL2-MxL4(151*5)
MxL3-MxL4(95*5)
Lineage(seq #)

S3.6 Fig.

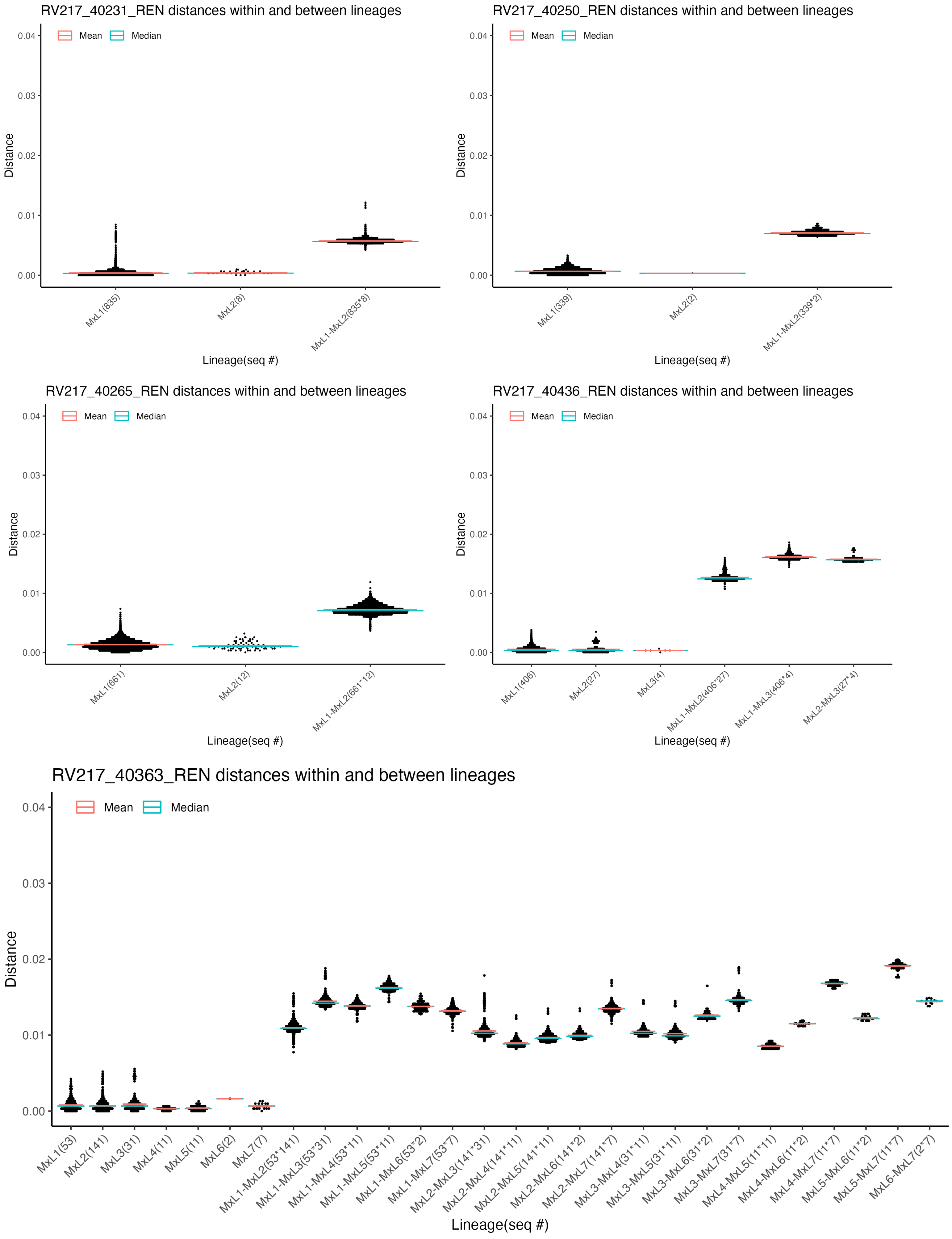
RV217_40231_REN distances within and between lineages
Mean
Median
Distance
0.04
0.03
0.02
0.01
0.00
MxL1(835)
MxL2(8)
MxL1-MxL2(835*8)
Lineage(seq #)
RV217_40250_REN distances within and between lineages
Mean
Median
Distance
MxL1(339)
MxL2(2)
MxL1-MxL2(339*2)
Lineage(seq #)
RV217_40265_REN distances within and between lineages
Mean
Median
Distance
MxL1(661)
MxL2(12)
MxL1-MxL2(661*12)
Lineage(seq #)
RV217_40436_REN distances within and between lineages
Mean
Median
Distance
MxL1(406)
MxL2(27)
MxL3(4)
MxL1-MxL2(406*27)
MxL1-MxL3(406*4)
MxL2-MxL3(27*4)
Lineage(seq #)
RV217_40363_REN distances within and between lineages
Mean
Median
Distance
MxL1(53)
MxL2(141)
MxL3(31)
MxL4(11)
MxL5(11)
MxL6(2)
MxL7(7)
MxL1-MxL2(53*141)
MxL1-MxL3(53*31)
MxL1-MxL4(53*11)
MxL1-MxL5(53*11)
MxL1-MxL6(53*2)
MxL1-MxL7(53*7)
MxL2-MxL3(141*31)
MxL2-MxL4(141*11)
MxL2-MxL5(141*11)
MxL2-MxL6(141*2)
MxL2-MxL7(141*7)
MxL3-MxL4(31*11)
MxL3-MxL5(31*11)
MxL3-MxL6(31*2)
MxL3-MxL7(31*7)
MxL4-MxL5(11*11)
MxL4-MxL6(11*2)
MxL4-MxL7(11*7)
MxL5-MxL6(11*2)
MxL5-MxL7(11*7)
MxL6-MxL7(2*7)
Lineage(seq #)

S3.7 Fig.

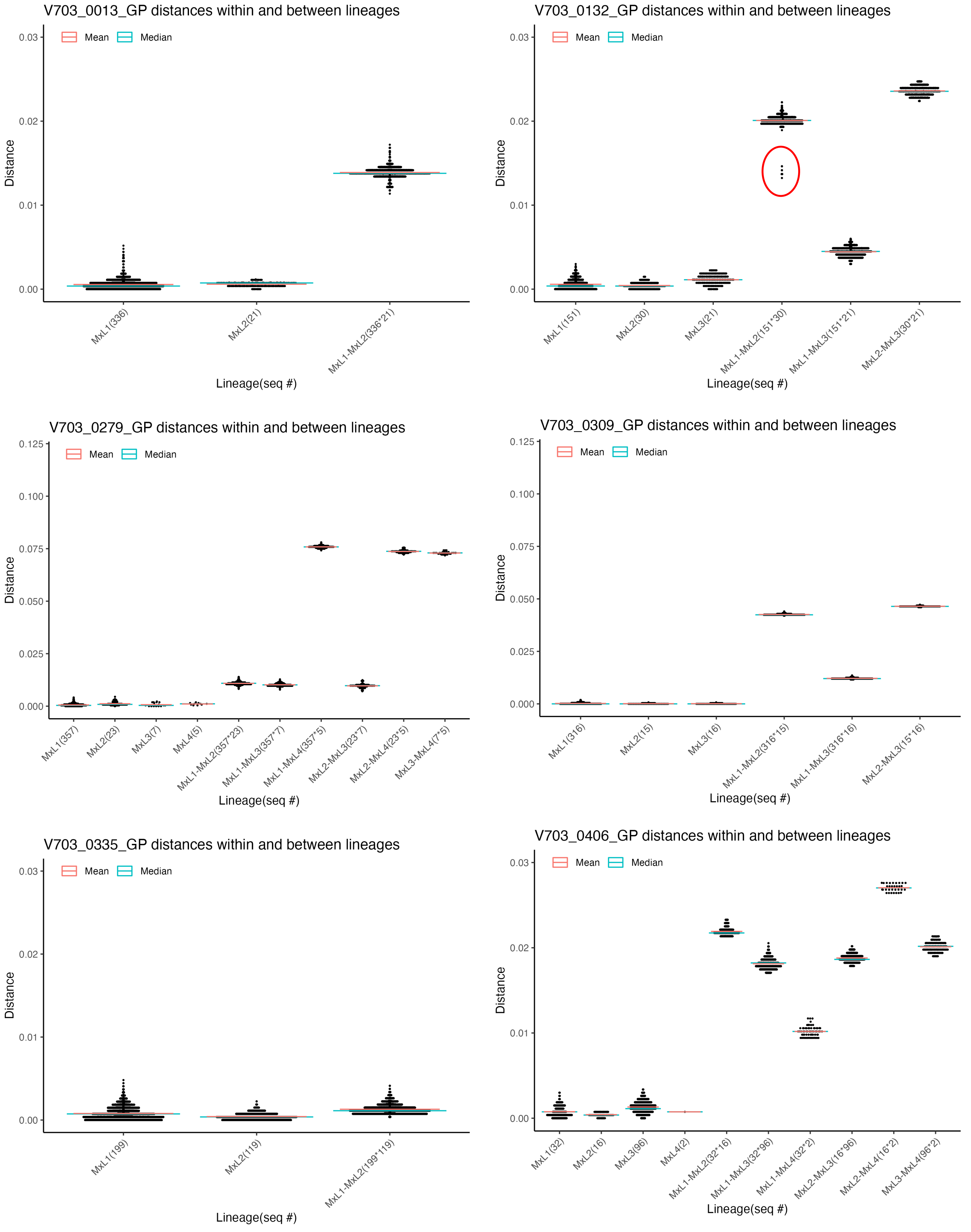
V703_0013_GP distances within and between lineages
Mean
Median
Distance
0.03
0.02
0.01
0.00
MxL1(336)
MxL2(21)
MxL1-MxL2(336*21)
Lineage(seq #)
V703_0132_GP distances within and between lineages
Mean
Median
Distance
0.03
0.02
0.01
0.00
MxL1(151)
MxL2(30)
MxL3(21)
MxL1-MxL2(151*30)
MxL1-MxL3(151*21)
MxL2-MxL3(30*21)
Lineage(seq #)
V703_0279_GP distances within and between lineages
Mean
Median
Distance
0.125
0.100
0.075
0.050
0.025
0.000
MxL1(357)
MxL2(23)
MxL3(7)
MxL4(5)
MxL1-MxL2(357*23)
MxL1-MxL3(357*7)
MxL1-MxL4(357*5)
MxL2-MxL3(23*7)
MxL2-MxL4(23*5)
MxL3-MxL4(7*5)
Lineage(seq #)
V703_0309_GP distances within and between lineages
Mean
Median
Distance
0.125
0.100
0.075
0.050
0.025
0.000
MxL1(316)
MxL2(15)
MxL3(16)
MxL1-MxL2(316*15)
MxL1-MxL3(316*16)
MxL2-MxL3(15*16)
Lineage(seq #)
V703_0335_GP distances within and between lineages
Mean
Median
Distance
0.03
0.02
0.01
0.00
MxL1(199)
MxL2(119)
MxL1-MxL2(199*119)
Lineage(seq #)
V703_0406_GP distances within and between lineages
Mean
Median
Distance
0.03
0.02
0.01
0.00
MxL1(32)
MxL2(16)
MxL3(96)
MxL4(2)
MxL1-MxL2(32*16)
MxL1-MxL3(32*96)
MxL1-MxL4(32*2)
MxL2-MxL3(16*96)
MxL2-MxL4(16*2)
MxL3-MxL4(96*2)
Lineage(seq #)

S3.8 Fig.

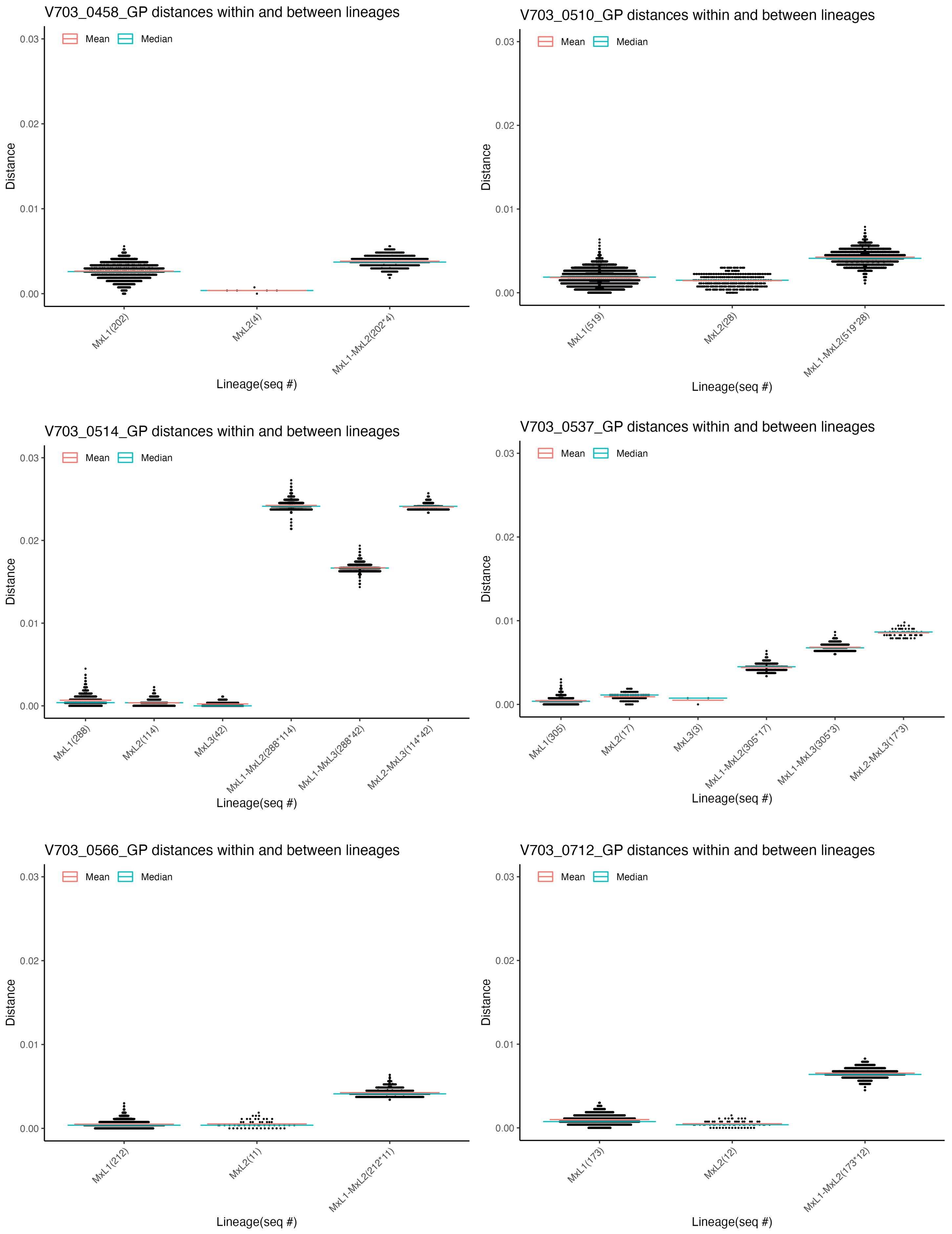
V703_0458_GP distances within and between lineages
Mean
Median
Distance
0.03
0.02
0.01
0.00
MxL1(202)
MxL2(4)
MxL1-MxL2(202*4)
Lineage(seq #)
V703_0510_GP distances within and between lineages
Mean
Median
Distance
0.03
0.02
0.01
0.00
MxL1(519)
MxL2(28)
MxL1-MxL2(519*28)
Lineage(seq #)
V703_0514_GP distances within and between lineages
Mean
Median
Distance
0.03
0.02
0.01
0.00
MxL1(288)
MxL2(114)
MxL3(42)
MxL1-MxL2(288*114)
MxL1-MxL3(288*42)
MxL2-MxL3(114*42)
Lineage(seq #)
V703_0537_GP distances within and between lineages
Mean
Median
Distance
0.03
0.02
0.01
0.00
MxL1(305)
MxL2(17)
MxL3(3)
MxL1-MxL2(305*17)
MxL1-MxL3(305*3)
MxL2-MxL3(17*3)
Lineage(seq #)
V703_0566_GP distances within and between lineages
Mean
Median
Distance
0.03
0.02
0.01
0.00
MxL1(212)
MxL2(11)
MxL1-MxL2(212*11)
Lineage(seq #)
V703_0712_GP distances within and between lineages
Mean
Median
Distance
0.03
0.02
0.01
0.00
MxL1(173)
MxL2(12)
MxL1-MxL2(173*12)
Lineage(seq #)

S3.9 Fig.

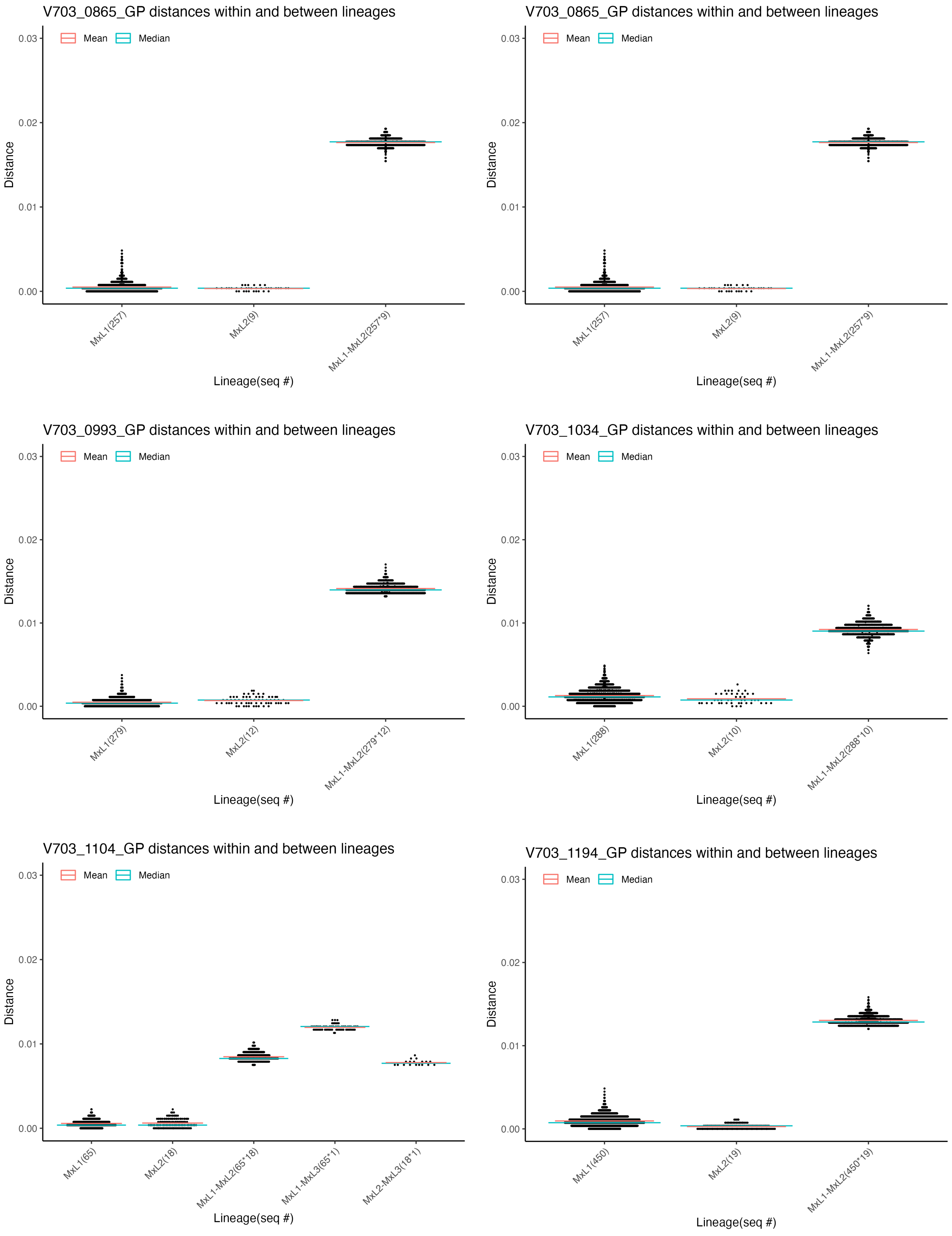
V703_0865_GP distances within and between lineages
Mean
Median
Distance
0.00
0.01
0.02
0.03
MxL1(257)
MxL2(9)
MxL1-MxL2(257*9)
Lineage(seq #)
V703_0865_GP distances within and between lineages
Mean
Median
Distance
MxL1(257)
MxL2(9)
MxL1-MxL2(257*9)
Lineage(seq #)
V703_0993_GP distances within and between lineages
Mean
Median
Distance
MxL1(279)
MxL2(12)
MxL1-MxL2(279*12)
Lineage(seq #)
V703_1034_GP distances within and between lineages
Mean
Median
Distance
MxL1(288)
MxL2(10)
MxL1-MxL2(288*10)
Lineage(seq #)
V703_1104_GP distances within and between lineages
Mean
Median
Distance
MxL1(65)
MxL2(18)
MxL1-MxL2(65*18)
MxL1-MxL3(65*1)
MxL2-MxL3(18*1)
Lineage(seq #)
V703_1194_GP distances within and between lineages
Mean
Median
Distance
MxL1(450)
MxL2(19)
MxL1-MxL2(450*19)
Lineage(seq #)

S3.10 Fig.

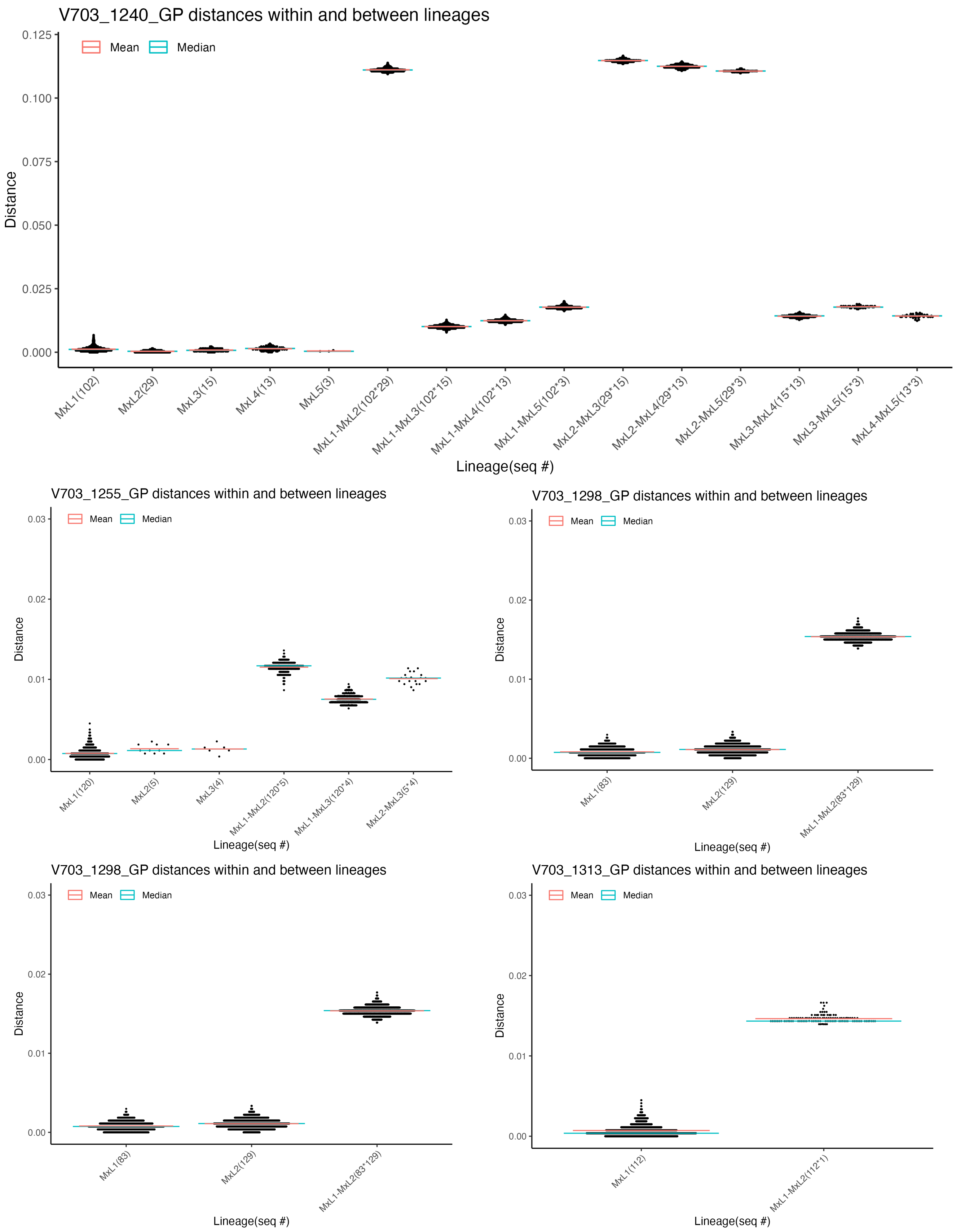

V703_1240_GP distances within and between lineages
Mean
Median
Distance
0.125
0.100
0.075
0.050
0.025
0.000
MxL1(102)
MxL2(29)
MxL3(15)
MxL4(13)
MxL5(3)
MxL1-MxL2(102*29)
MxL1-MxL3(102*15)
MxL1-MxL4(102*13)
MxL1-MxL5(102*3)
MxL2-MxL3(29*15)
MxL2-MxL4(29*13)
MxL2-MxL5(29*3)
MxL3-MxL4(15*13)
MxL3-MxL5(15*3)
MxL4-MxL5(13*3)
Lineage(seq #)
V703_1255_GP distances within and between lineages
Mean
Median
Distance
0.03
0.02
0.01
0.00
MxL1(120)
MxL2(5)
MxL3(4)
MxL1-MxL2(120*5)
MxL1-MxL3(120*4)
MxL2-MxL3(5*4)
Lineage(seq #)
V703_1298_GP distances within and between lineages
Mean
Median
Distance
0.03
0.02
0.01
0.00
MxL1(83)
MxL2(129)
MxL1-MxL2(83*129)
Lineage(seq #)
V703_1298_GP distances within and between lineages
Mean
Median
Distance
0.03
0.02
0.01
0.00
MxL1(83)
MxL2(129)
MxL1-MxL2(83*129)
Lineage(seq #)
V703_1313_GP distances within and between lineages
Mean
Median
Distance
0.03
0.02
0.01
0.00
MxL1(112)
MxL1-MxL2(112*1)
Lineage(seq #)

S3.11 Fig.

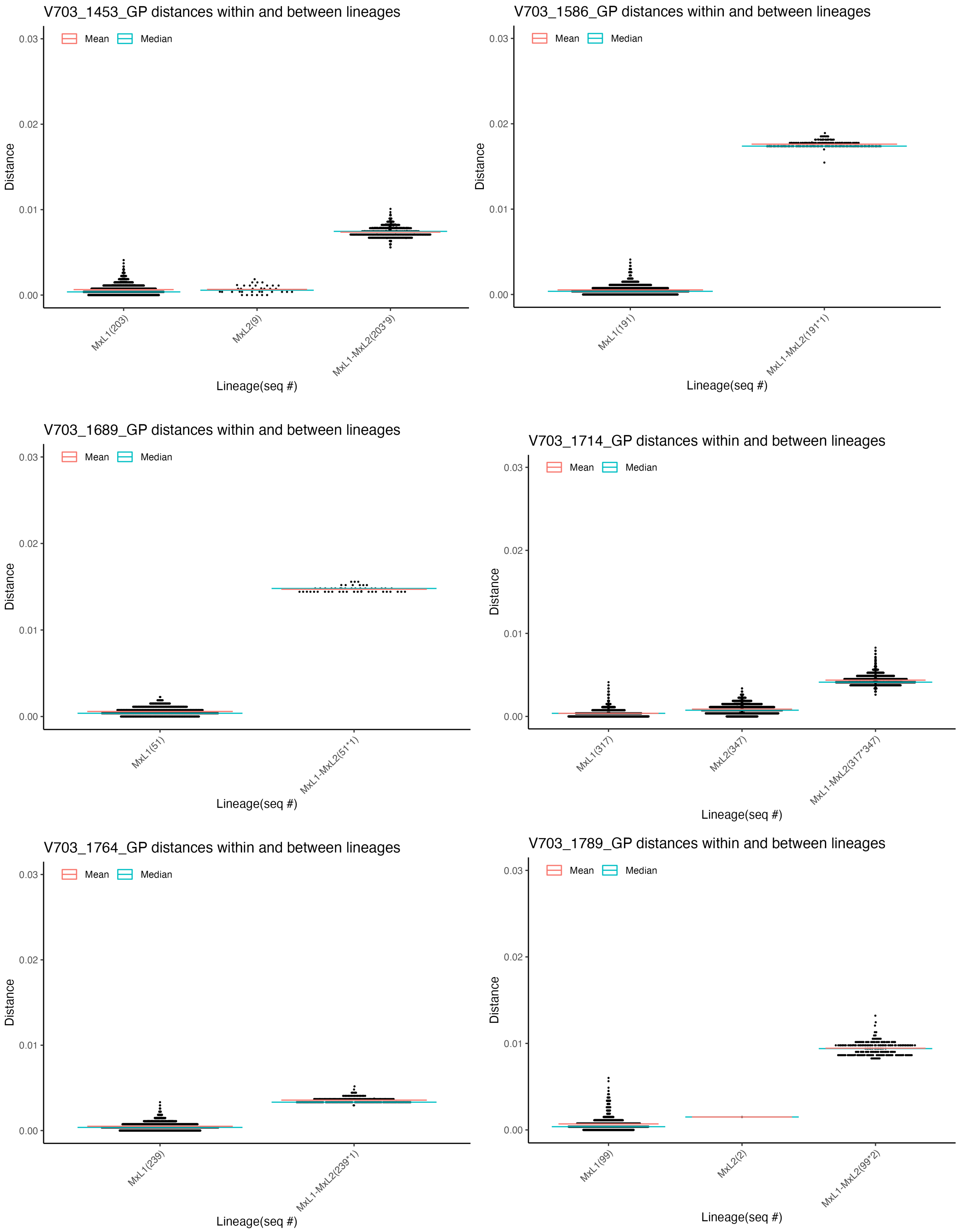
V703_1453_GP distances within and between lineages
Mean
Median
Distance
0.03
0.02
0.01
0.00
MxL1(203)
MxL2(9)
MxL1-MxL2(203*9)
Lineage(seq #)
V703_1586_GP distances within and between lineages
Mean
Median
Distance
0.03
0.02
0.01
0.00
MxL1(191)
MxL1-MxL2(191*1)
Lineage(seq #)
V703_1689_GP distances within and between lineages
Mean
Median
Distance
0.03
0.02
0.01
0.00
MxL1(51)
MxL1-MxL2(51*1)
Lineage(seq #)
V703_1714_GP distances within and between lineages
Mean
Median
Distance
0.03
0.02
0.01
0.00
MxL1(317)
MxL2(347)
MxL1-MxL2(317*347)
Lineage(seq #)
V703_1764_GP distances within and between lineages
Mean
Median
Distance
0.03
0.02
0.01
0.00
MxL1(239)
MxL1-MxL2(239*1)
Lineage(seq #)
V703_1789_GP distances within and between lineages
Mean
Median
Distance
0.03
0.02
0.01
0.00
MxL1(99)
MxL2(2)
MxL1-MxL2(99*2)
Lineage(seq #)

S3.12 Fig.

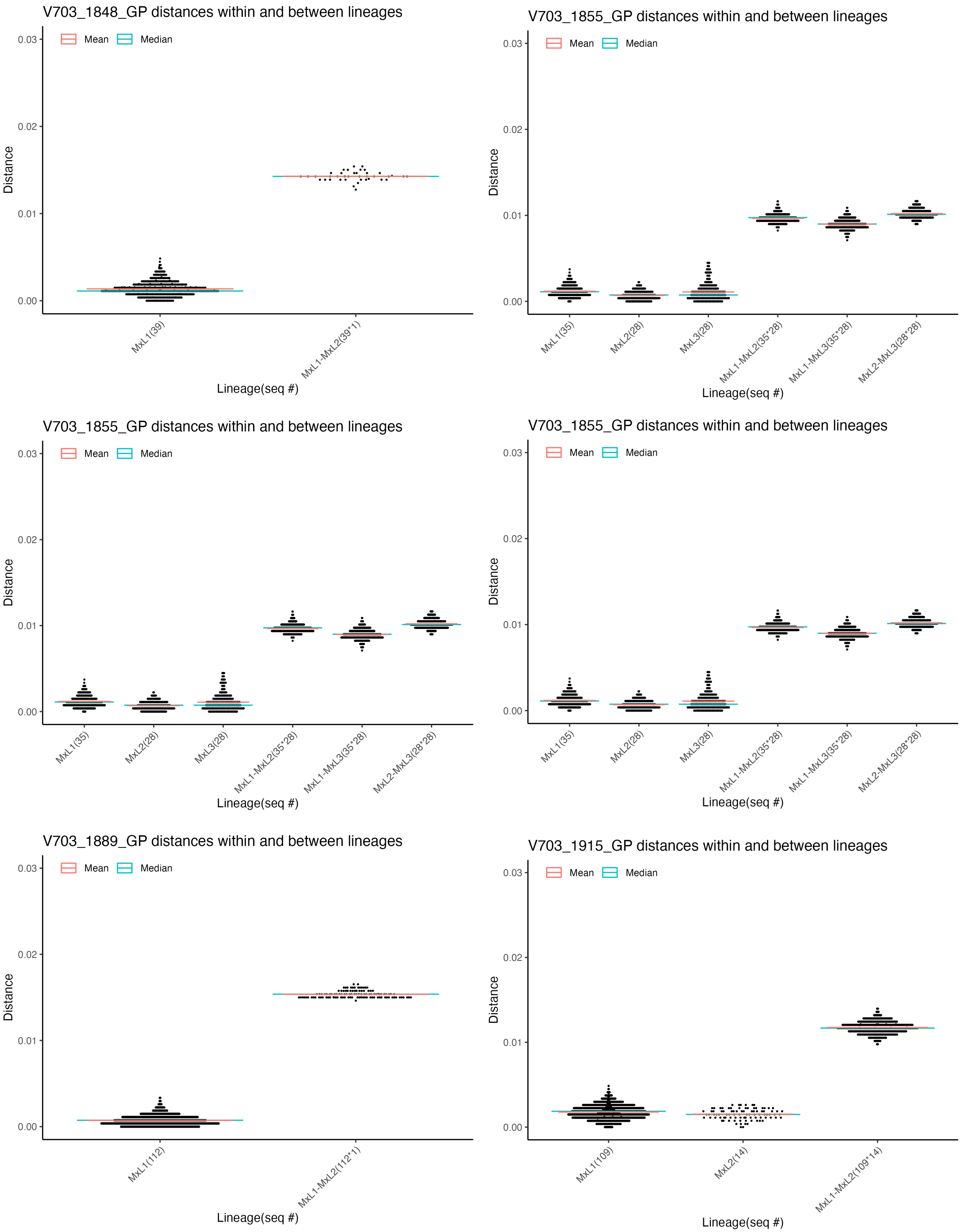
V703_1848_GP distances within and between lineages
Mean
Median
Distance
0.00
0.01
0.02
0.03
MxL1(39)
MxL1-MxL2(39*1)
Lineage(seq #)
V703_1855_GP distances within and between lineages
MxL1(35)
MxL2(28)
MxL3(28)
MxL1-MxL2(35*28)
MxL1-MxL3(35*28)
MxL2-MxL3(28*28)
V703_1889_GP distances within and between lineages
MxL1(112)
MxL1-MxL2(112*1)
V703_1915_GP distances within and between lineages
MxL1(109)
MxL2(14)
MxL1-MxL2(109*14)

S3.13 Fig.

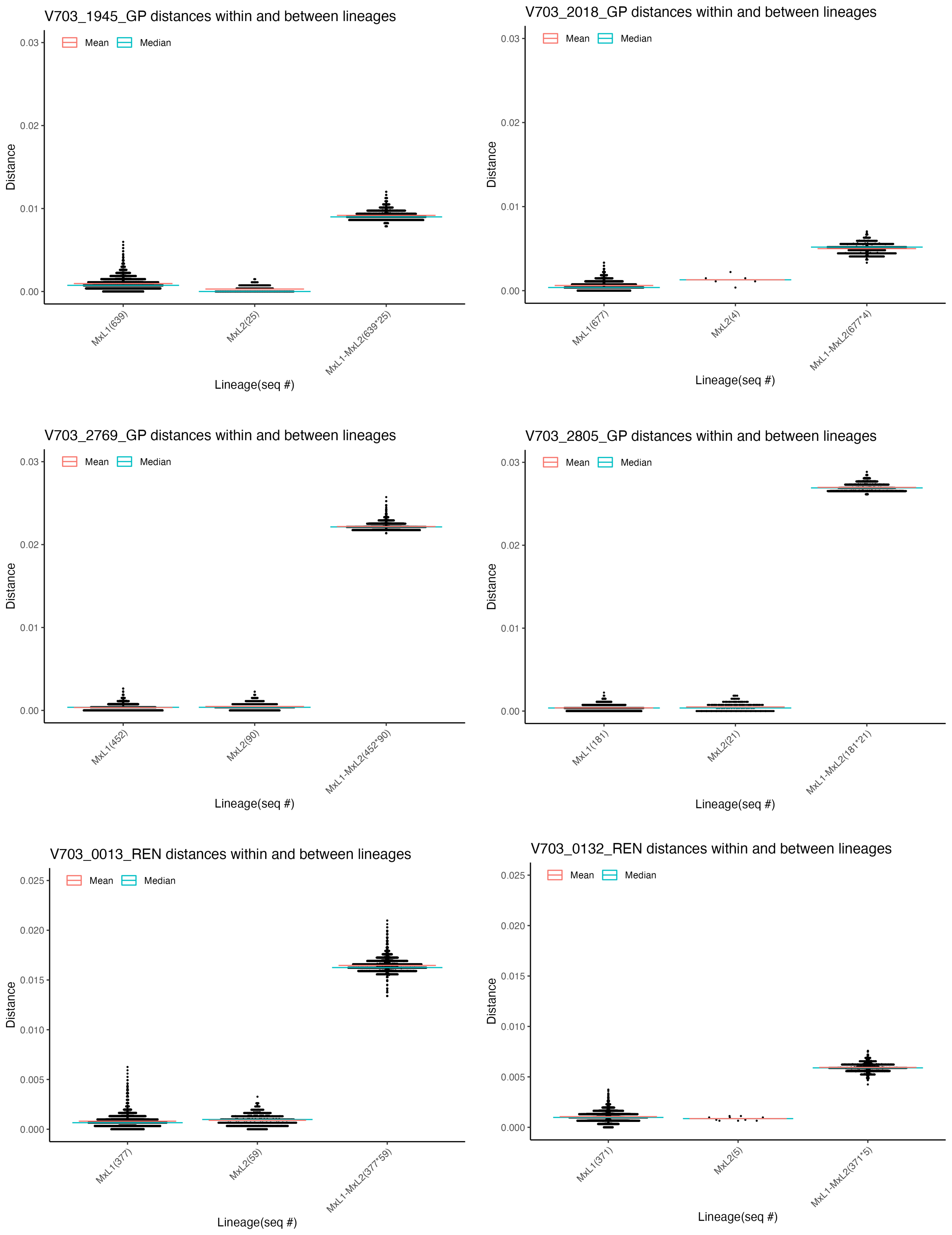

V703_1945_GP distances within and between lineages
Mean
Median
Distance
0.03
0.02
0.01
0.00
MxL1(639)
MxL2(25)
MxL1-MxL2(639*25)
Lineage(seq #)
V703_2018_GP distances within and between lineages
MxL1(677)
MxL2(4)
MxL1-MxL2(677*4)
V703_2769_GP distances within and between lineages
MxL1(452)
MxL2(90)
MxL1-MxL2(452*90)
V703_2805_GP distances within and between lineages
MxL1(181)
MxL2(21)
MxL1-MxL2(181*21)
V703_0013_REN distances within and between lineages
0.025
0.020
0.015
0.010
0.005
0.000
MxL1(377)
MxL2(59)
MxL1-MxL2(377*59)
V703_0132_REN distances within and between lineages
MxL1(371)
MxL2(5)
MxL1-MxL2(371*5)

S3.14 Fig.

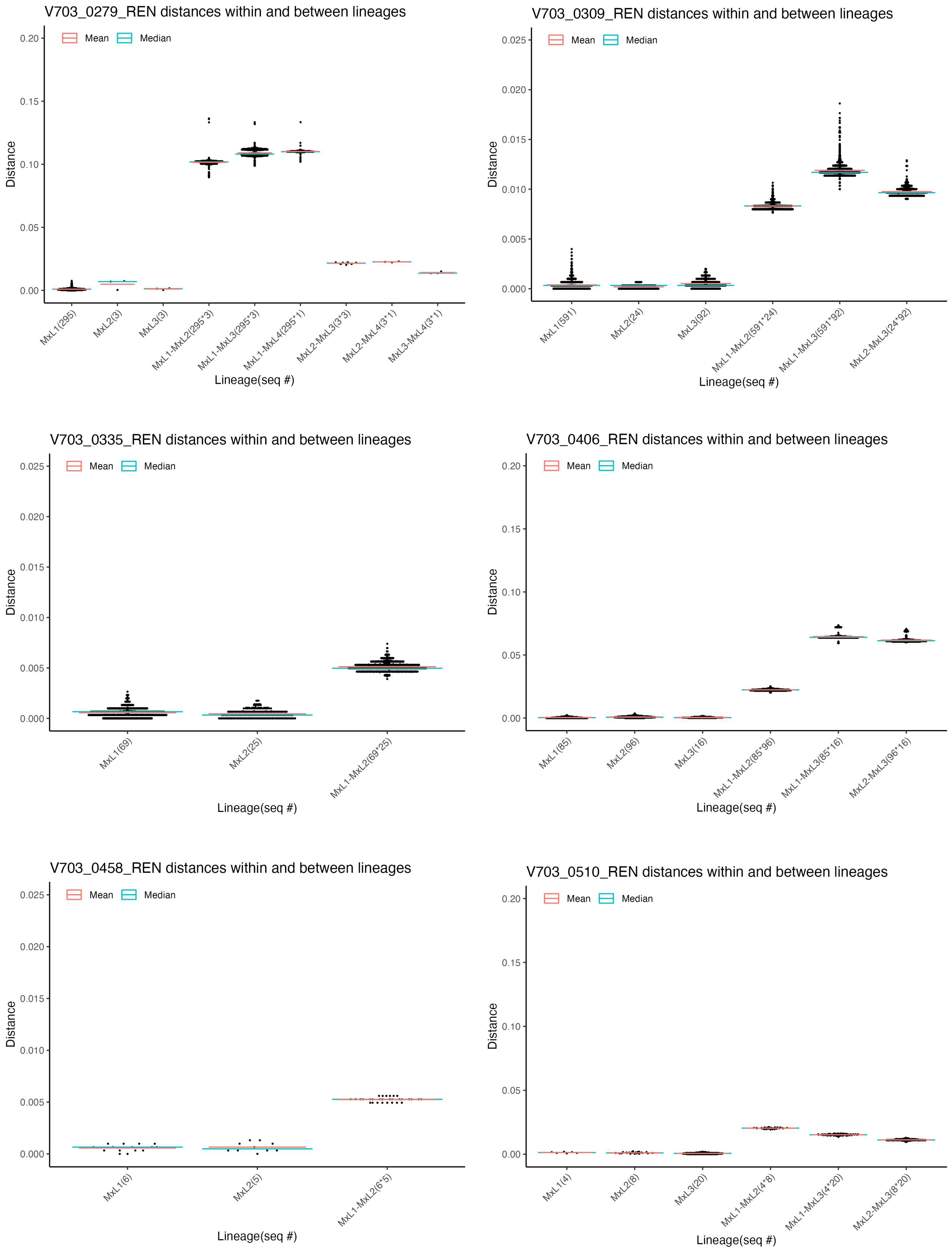
V703_0279_REN distances within and between lineages
Mean
Median
Distance
Lineage(seq #)
MxL1(295)
MxL2(3)
MxL3(3)
MxL1-MxL2(295*3)
MxL1-MxL3(295*3)
MxL1-MxL4(295*1)
MxL2-MxL3(3*3)
MxL2-MxL4(3*1)
MxL3-MxL4(3*1)
V703_0309_REN distances within and between lineages
MxL1(591)
MxL2(24)
MxL3(92)
MxL1-MxL2(591*24)
MxL1-MxL3(591*92)
MxL2-MxL3(24*92)
V703_0335_REN distances within and between lineages
MxL1(69)
MxL2(25)
MxL1-MxL2(69*25)
V703_0406_REN distances within and between lineages
MxL1(85)
MxL2(96)
MxL3(16)
MxL1-MxL2(85*96)
MxL1-MxL3(85*16)
MxL2-MxL3(96*16)
V703_0458_REN distances within and between lineages
MxL1(6)
MxL2(5)
MxL1-MxL2(6*5)
V703_0510_REN distances within and between lineages
MxL1(4)
MxL2(8)
MxL3(20)
MxL1-MxL2(4*8)
MxL1-MxL3(4*20)
MxL2-MxL3(8*20)

S3.15 Fig.

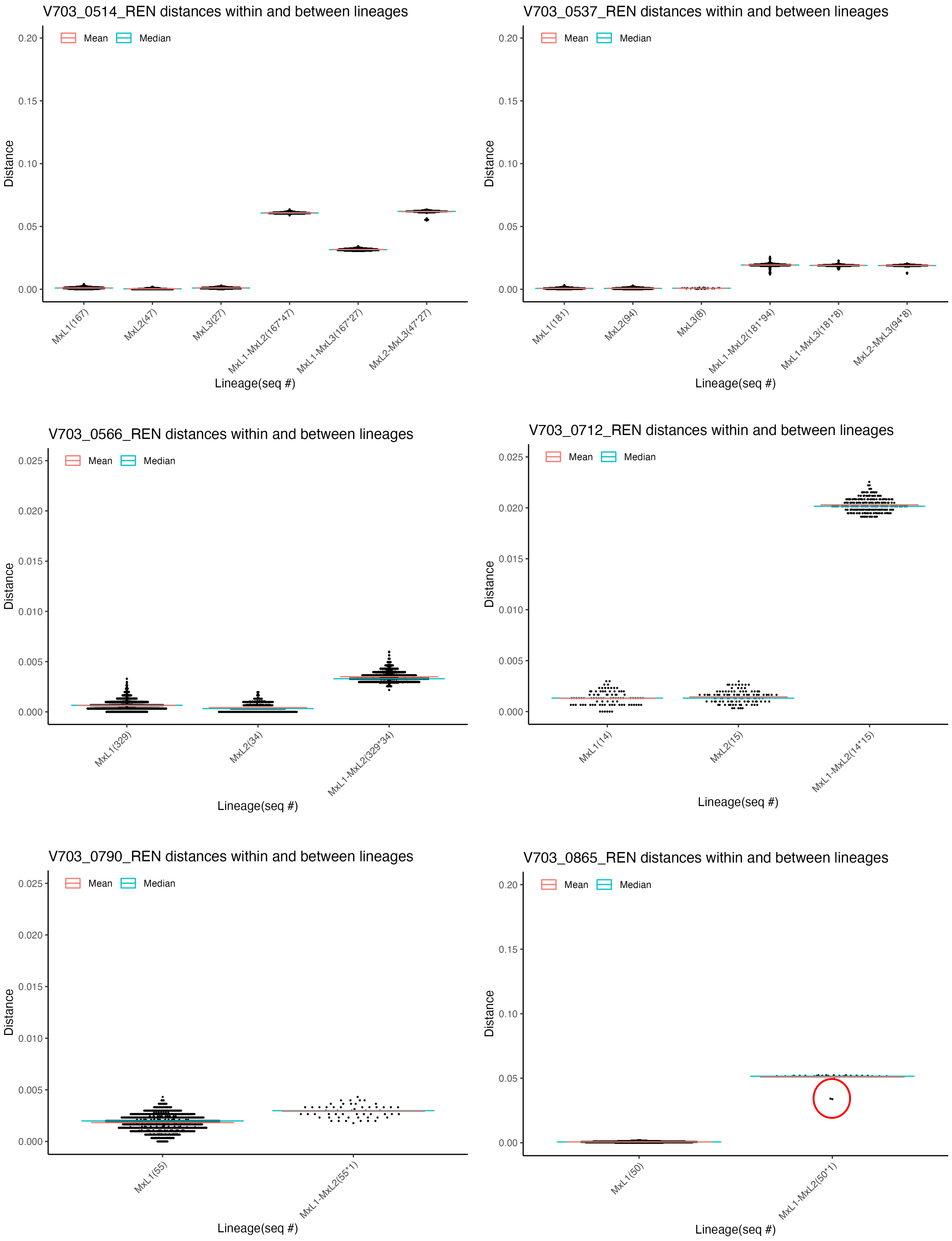

V703_0514_REN distances within and between lineages
Mean
Median
Distance
0.20
0.15
0.10
0.05
0.00
MxL1(167)
MxL2(47)
MxL3(27)
MxL1-MxL2(167*47)
MxL1-MxL3(167*27)
MxL2-MxL3(47*27)
Lineage(seq #)
V703_0537_REN distances within and between lineages
Mean
Median
Distance
0.20
0.15
0.10
0.05
0.00
MxL1(181)
MxL2(94)
MxL3(8)
MxL1-MxL2(181*94)
MxL1-MxL3(181*8)
MxL2-MxL3(94*8)
Lineage(seq #)
V703_0566_REN distances within and between lineages
Mean
Median
Distance
0.025
0.020
0.015
0.010
0.005
0.000
MxL1(329)
MxL2(34)
MxL1-MxL2(329*34)
Lineage(seq #)
V703_0712_REN distances within and between lineages
Mean
Median
Distance
0.025
0.020
0.015
0.010
0.005
0.000
MxL1(14)
MxL2(15)
MxL1-MxL2(14*15)
Lineage(seq #)
V703_0790_REN distances within and between lineages
Mean
Median
Distance
0.025
0.020
0.015
0.010
0.005
0.000
MxL1(55)
MxL1-MxL2(55*1)
Lineage(seq #)
V703_0865_REN distances within and between lineages
Mean
Median
Distance
0.20
0.15
0.10
0.05
0.00
MxL1(50)
MxL1-MxL2(50*1)
Lineage(seq #)

S3.16 Fig.

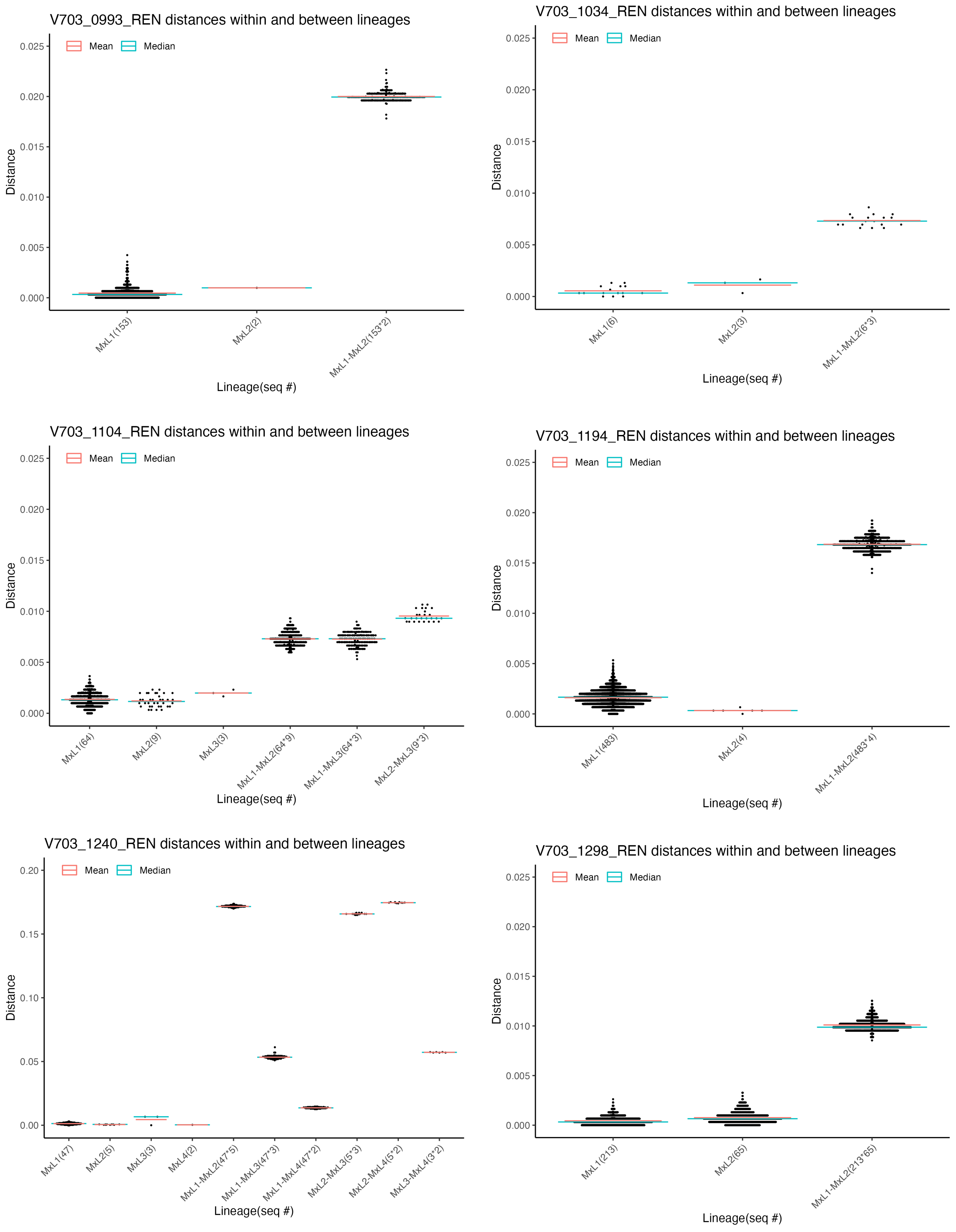
V703_0993_REN distances within and between lineages
Mean
Median
Distance
0.025
0.020
0.015
0.010
0.005
0.000
MxL1(153)
MxL2(2)
MxL1-MxL2(153*2)
Lineage(seq #)
V703_1034_REN distances within and between lineages
Mean
Median
Distance
0.025
0.020
0.015
0.010
0.005
0.000
MxL1(6)
MxL2(3)
MxL1-MxL2(6*3)
Lineage(seq #)
V703_1104_REN distances within and between lineages
Mean
Median
Distance
0.025
0.020
0.015
0.010
0.005
0.000
MxL1(64)
MxL2(9)
MxL3(3)
MxL1-MxL2(64*9)
MxL1-MxL3(64*3)
MxL2-MxL3(9*3)
Lineage(seq #)
V703_1194_REN distances within and between lineages
Mean
Median
Distance
0.025
0.020
0.015
0.010
0.005
0.000
MxL1(483)
MxL2(4)
MxL1-MxL2(483*4)
Lineage(seq #)
V703_1240_REN distances within and between lineages
Mean
Median
Distance
0.20
0.15
0.10
0.05
0.00
MxL1(47)
MxL2(5)
MxL3(3)
MxL4(2)
MxL1-MxL2(47*5)
MxL1-MxL3(47*3)
MxL1-MxL4(47*2)
MxL2-MxL3(5*3)
MxL2-MxL4(5*2)
MxL3-MxL4(3*2)
Lineage(seq #)
V703_1298_REN distances within and between lineages
Mean
Median
Distance
0.025
0.020
0.015
0.010
0.005
0.000
MxL1(213)
MxL2(65)
MxL1-MxL2(213*65)
Lineage(seq #)

S3.17 Fig.

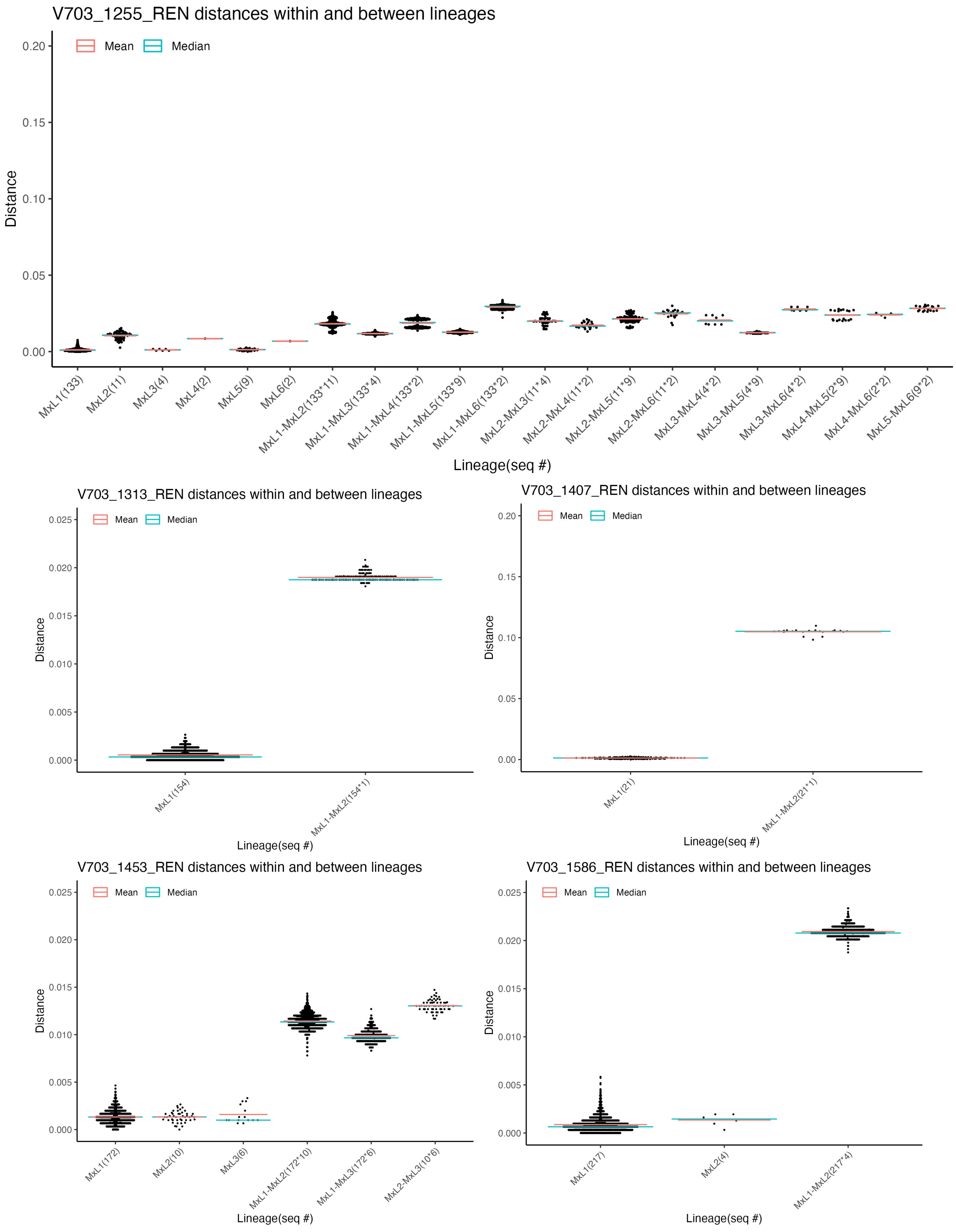

V703_1255_REN distances within and between lineages
Mean
Median
Distance
0.20
0.15
0.10
0.05
0.00
MxL1(133)
MxL2(11)
MxL3(4)
MxL4(2)
MxL5(9)
MxL6(2)
MxL1-MxL2(133*11)
MxL1-MxL3(133*4)
MxL1-MxL4(133*2)
MxL1-MxL5(133*9)
MxL1-MxL6(133*2)
MxL2-MxL3(11*4)
MxL2-MxL4(11*2)
MxL2-MxL5(11*9)
MxL2-MxL6(11*2)
MxL3-MxL4(4*2)
MxL3-MxL5(4*9)
MxL3-MxL6(4*2)
MxL4-MxL5(2*9)
MxL4-MxL6(2*2)
MxL5-MxL6(9*2)
Lineage(seq #)
V703_1313_REN distances within and between lineages
Mean
Median
Distance
0.025
0.020
0.015
0.010
0.005
0.000
MxL1(154)
MxL1-MxL2(154*1)
Lineage(seq #)
V703_1407_REN distances within and between lineages
Mean
Median
Distance
0.20
0.15
0.10
0.05
0.00
MxL1(21)
MxL1-MxL2(21*1)
Lineage(seq #)
V703_1453_REN distances within and between lineages
Mean
Median
Distance
0.025
0.020
0.015
0.010
0.005
0.000
MxL1(172)
MxL2(10)
MxL3(6)
MxL1-MxL2(172*10)
MxL1-MxL3(172*6)
MxL2-MxL3(10*6)
Lineage(seq #)
V703_1586_REN distances within and between lineages
Mean
Median
Distance
0.025
0.020
0.015
0.010
0.005
0.000
MxL1(217)
MxL2(4)
MxL1-MxL2(217*4)
Lineage(seq #)

S3.18 Fig.

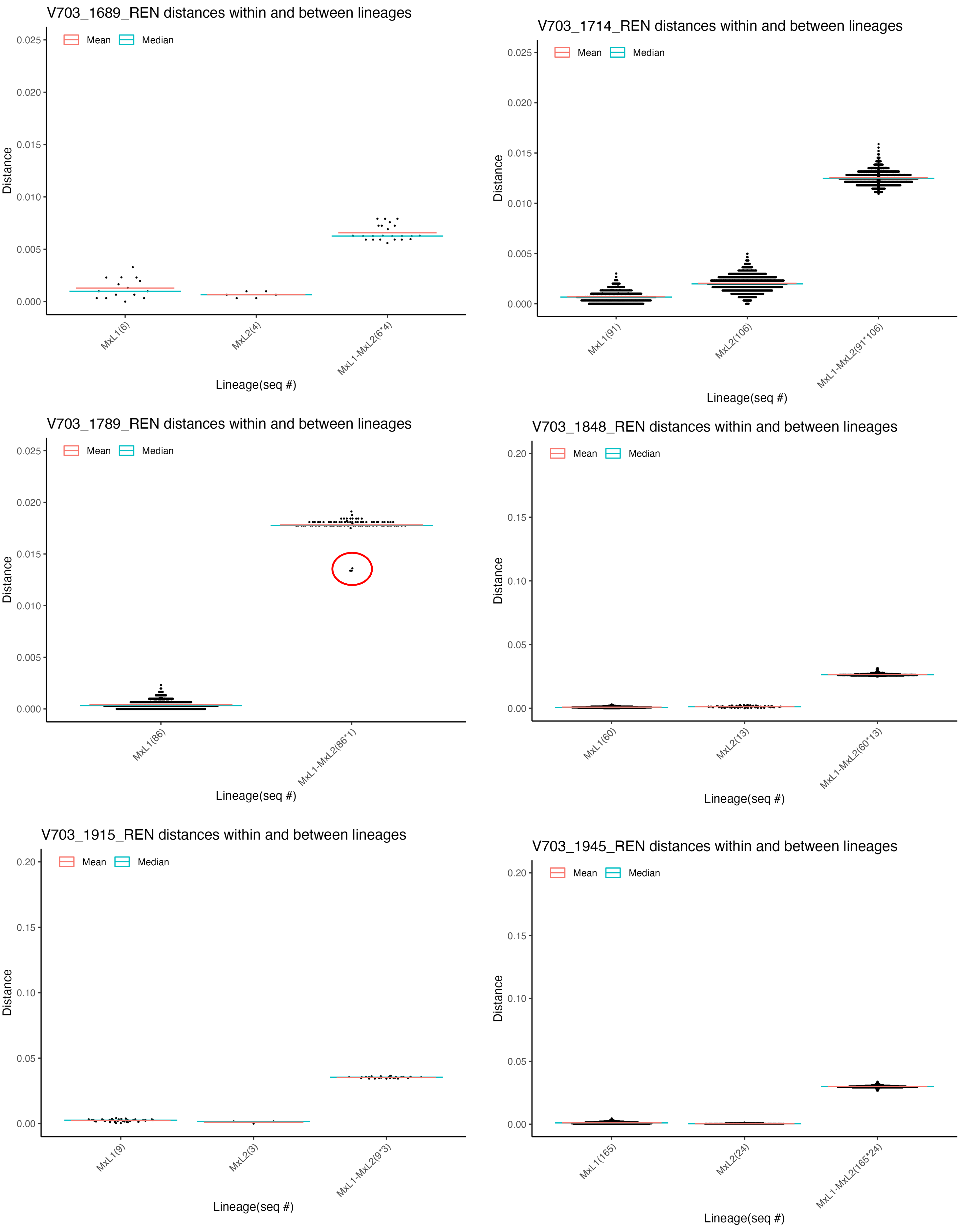
V703_1689_REN distances within and between lineages
Mean
Median
Distance
0.025
0.020
0.015
0.010
0.005
0.000
MxL1(6)
MxL2(4)
MxL1-MxL2(6*4)
Lineage(seq #)
V703_1714_REN distances within and between lineages
Mean
Median
Distance
0.025
0.020
0.015
0.010
0.005
0.000
MxL1(91)
MxL2(106)
MxL1-MxL2(91*106)
Lineage(seq #)
V703_1789_REN distances within and between lineages
Mean
Median
Distance
0.025
0.020
0.015
0.010
0.005
0.000
MxL1(86)
MxL1-MxL2(86*1)
Lineage(seq #)
V703_1848_REN distances within and between lineages
Mean
Median
Distance
0.20
0.15
0.10
0.05
0.00
MxL1(60)
MxL2(13)
MxL1-MxL2(60*13)
Lineage(seq #)
V703_1915_REN distances within and between lineages
Mean
Median
Distance
0.20
0.15
0.10
0.05
0.00
MxL1(9)
MxL2(3)
MxL1-MxL2(9*3)
Lineage(seq #)
V703_1945_REN distances within and between lineages
Mean
Median
Distance
0.20
0.15
0.10
0.05
0.00
MxL1(165)
MxL2(24)
MxL1-MxL2(165*24)
Lineage(seq #)

S3.19 Fig.

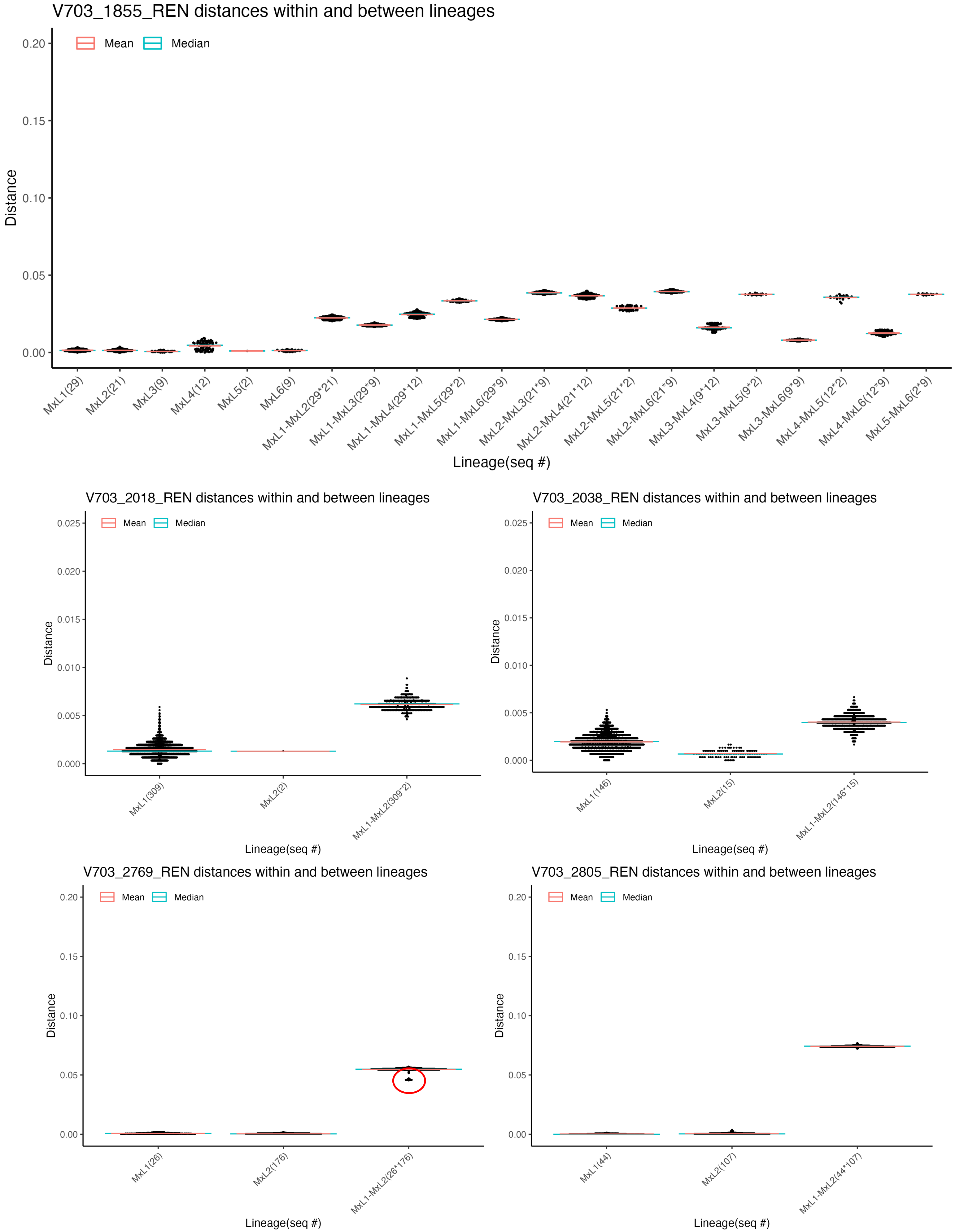
V703_1855_REN distances within and between lineages
Mean
Median
Distance
0.20
0.15
0.10
0.05
0.00
MxL1(29)
MxL2(21)
MxL3(9)
MxL4(12)
MxL5(2)
MxL6(9)
MxL1-MxL2(29*21)
MxL1-MxL3(29*9)
MxL1-MxL4(29*12)
MxL1-MxL5(29*2)
MxL1-MxL6(29*9)
MxL2-MxL3(21*9)
MxL2-MxL4(21*12)
MxL2-MxL5(21*2)
MxL2-MxL6(21*9)
MxL3-MxL4(9*12)
MxL3-MxL5(9*2)
MxL3-MxL6(9*9)
MxL4-MxL5(12*2)
MxL4-MxL6(12*9)
MxL5-MxL6(2*9)
Lineage(seq #)
V703_2018_REN distances within and between lineages
Mean
Median
Distance
0.025
0.020
0.015
0.010
0.005
0.000
MxL1(309)
MxL2(2)
MxL1-MxL2(309*2)
Lineage(seq #)
V703_2038_REN distances within and between lineages
Mean
Median
Distance
0.025
0.020
0.015
0.010
0.005
0.000
MxL1(146)
MxL2(15)
MxL1-MxL2(146*15)
Lineage(seq #)
V703_2769_REN distances within and between lineages
Mean
Median
Distance
0.20
0.15
0.10
0.05
0.00
MxL1(26)
MxL2(176)
MxL1-MxL2(26*176)
Lineage(seq #)
V703_2805_REN distances within and between lineages
Mean
Median
Distance
0.20
0.15
0.10
0.05
0.00
MxL1(44)
MxL2(107)
MxL1-MxL2(44*107)
Lineage(seq #)

S3.20 Fig.

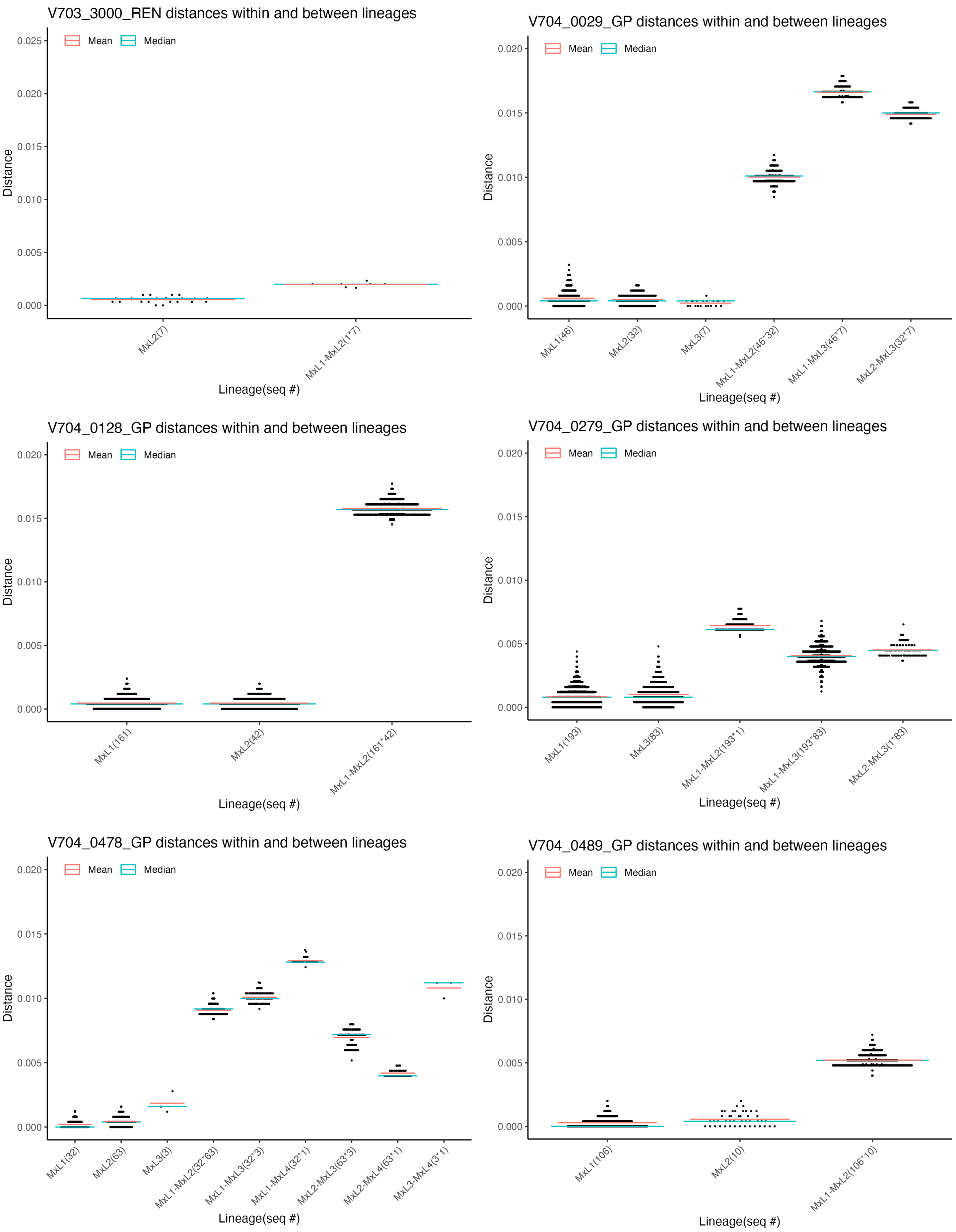

V703_3000_REN distances within and between lineages
Mean
Median
Distance
MxL2(7)
MxL1-MxL2(1*7)
Lineage(seq #)
V704_0029_GP distances within and between lineages
Mean
Median
Distance
MxL1(46)
MxL2(32)
MxL3(7)
MxL1-MxL2(46*32)
MxL1-MxL3(46*7)
MxL2-MxL3(32*7)
Lineage(seq #)
V704_0128_GP distances within and between lineages
Mean
Median
Distance
MxL1(161)
MxL2(42)
MxL1-MxL2(161*42)
Lineage(seq #)
V704_0279_GP distances within and between lineages
Mean
Median
Distance
MxL1(193)
MxL3(83)
MxL1-MxL2(193*1)
MxL1-MxL3(193*83)
MxL2-MxL3(1*83)
Lineage(seq #)
V704_0478_GP distances within and between lineages
Mean
Median
Distance
MxL1(32)
MxL2(63)
MxL3(3)
MxL1-MxL2(32*63)
MxL1-MxL3(32*3)
MxL1-MxL4(32*1)
MxL2-MxL3(63*3)
MxL2-MxL4(63*1)
MxL3-MxL4(3*1)
Lineage(seq #)
V704_0489_GP distances within and between lineages
Mean
Median
Distance
MxL1(106)
MxL2(10)
MxL1-MxL2(106*10)
Lineage(seq #)

S3.21 Fig.

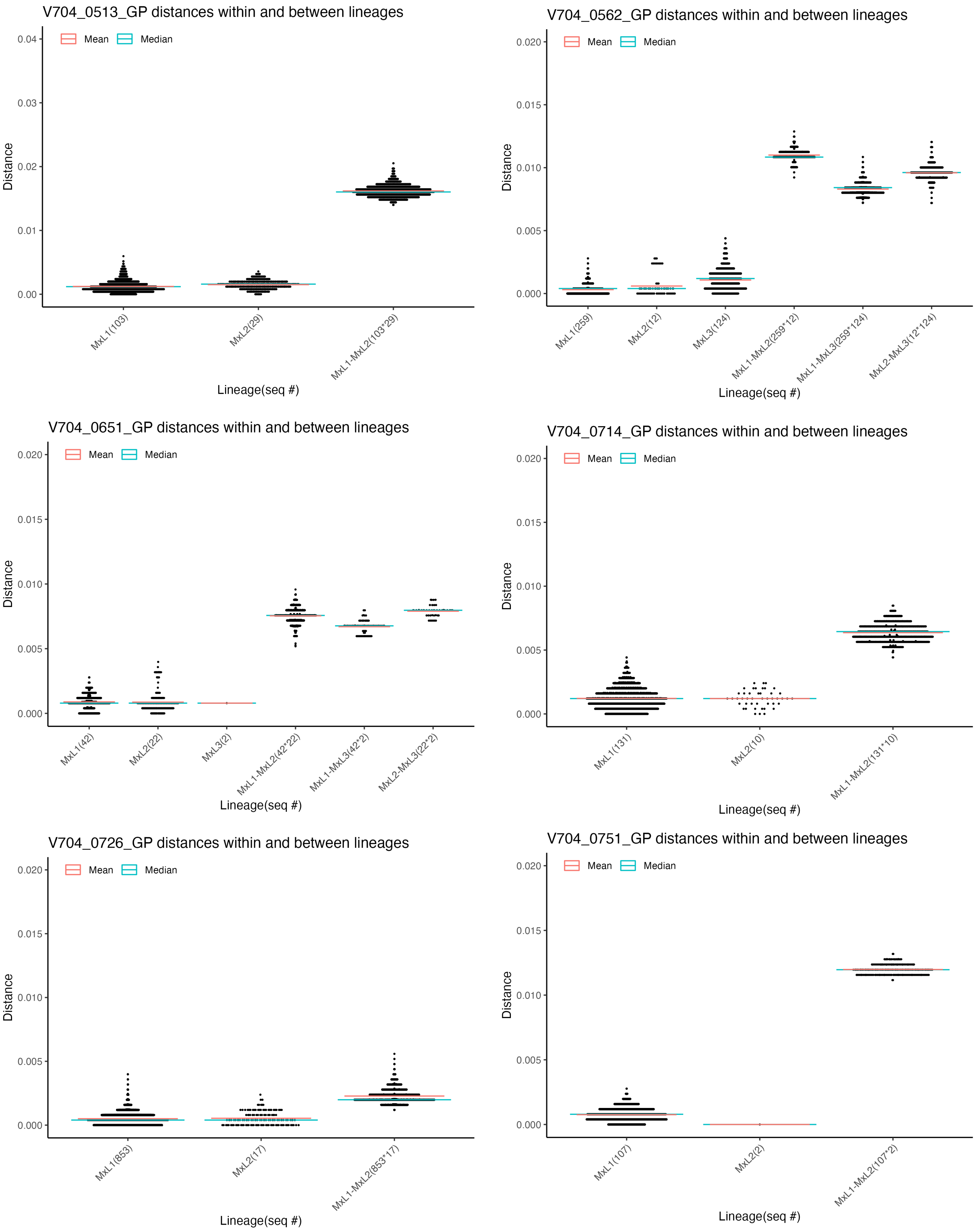
V704_0513_GP distances within and between lineages
Mean
Median
Distance
0.04
0.03
0.02
0.01
0.00
MxL1(103)
MxL2(29)
MxL1-MxL2(103*29)
Lineage(seq #)
V704_0562_GP distances within and between lineages
Mean
Median
Distance
0.020
0.015
0.010
0.005
0.000
MxL1(259)
MxL2(12)
MxL3(124)
MxL1-MxL2(259*12)
MxL1-MxL3(259*124)
MxL2-MxL3(12*124)
Lineage(seq #)
V704_0651_GP distances within and between lineages
Mean
Median
Distance
0.020
0.015
0.010
0.005
0.000
MxL1(42)
MxL2(22)
MxL3(2)
MxL1-MxL2(42*22)
MxL1-MxL3(42*2)
MxL2-MxL3(22*2)
Lineage(seq #)
V704_0714_GP distances within and between lineages
Mean
Median
Distance
0.020
0.015
0.010
0.005
0.000
MxL1(131)
MxL2(10)
MxL1-MxL2(131*10)
Lineage(seq #)
V704_0726_GP distances within and between lineages
Mean
Median
Distance
0.020
0.015
0.010
0.005
0.000
MxL1(853)
MxL2(17)
MxL1-MxL2(853*17)
Lineage(seq #)
V704_0751_GP distances within and between lineages
Mean
Median
Distance
0.020
0.015
0.010
0.005
0.000
MxL1(107)
MxL2(2)
MxL1-MxL2(107*2)
Lineage(seq #)

S3.22 Fig.

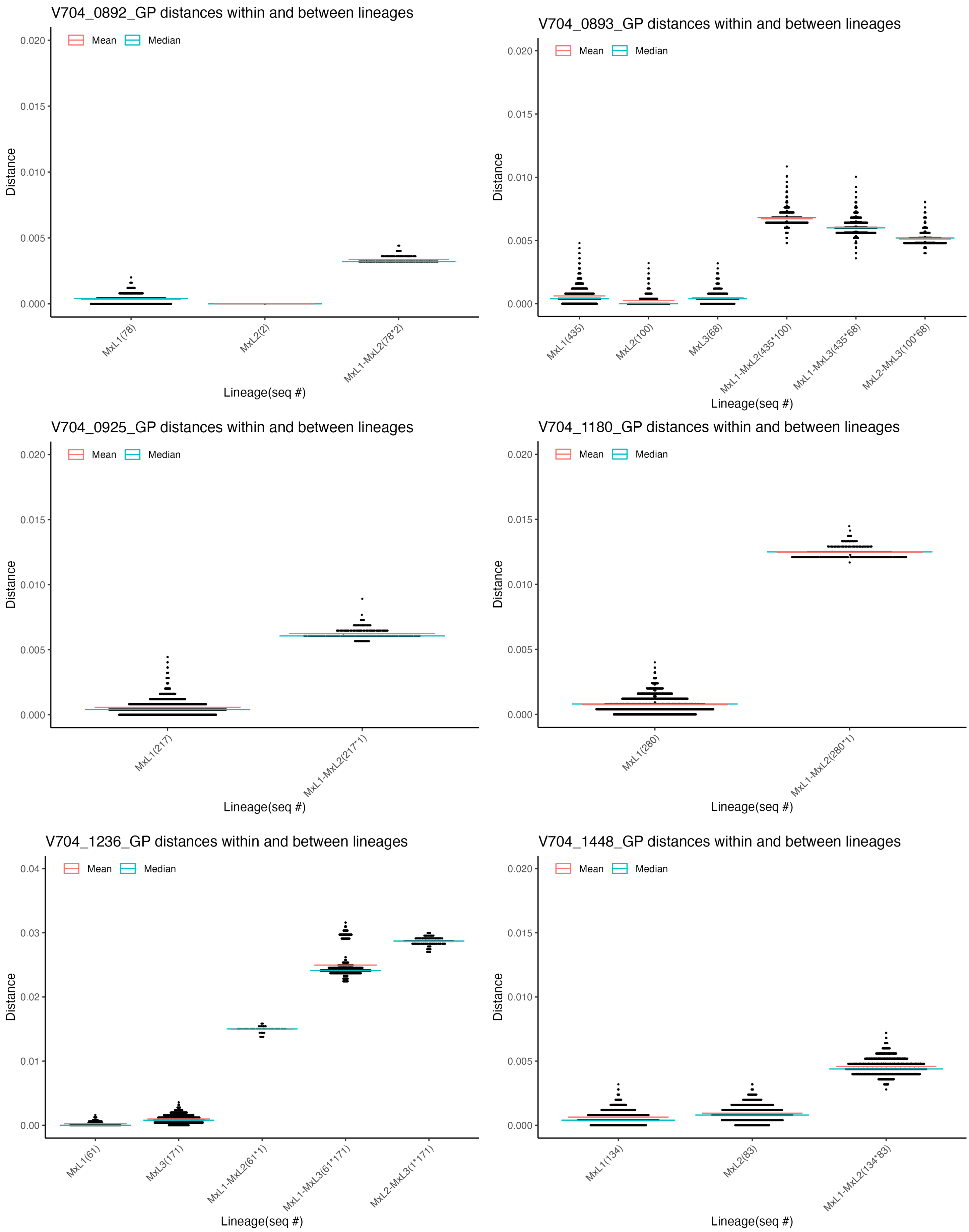
V704_0892_GP distances within and between lineages
Mean
Median
Distance
0.020
0.015
0.010
0.005
0.000
MxL1(78)
MxL2(2)
MxL1-MxL2(78*2)
Lineage(seq #)
V704_0893_GP distances within and between lineages
Mean
Median
Distance
0.020
0.015
0.010
0.005
0.000
MxL1(435)
MxL2(100)
MxL3(68)
MxL1-MxL2(435*100)
MxL1-MxL3(435*68)
MxL2-MxL3(100*68)
Lineage(seq #)
V704_0925_GP distances within and between lineages
Mean
Median
Distance
0.020
0.015
0.010
0.005
0.000
MxL1(217)
MxL1-MxL2(217*1)
Lineage(seq #)
V704_1180_GP distances within and between lineages
Mean
Median
Distance
0.020
0.015
0.010
0.005
0.000
MxL1(280)
MxL1-MxL2(280*1)
Lineage(seq #)
V704_1236_GP distances within and between lineages
Mean
Median
Distance
0.04
0.03
0.02
0.01
0.00
MxL1(61)
MxL3(171)
MxL1-MxL2(61*1)
MxL1-MxL3(61*171)
MxL2-MxL3(1*171)
Lineage(seq #)
V704_1448_GP distances within and between lineages
Mean
Median
Distance
0.020
0.015
0.010
0.005
0.000
MxL1(134)
MxL2(83)
MxL1-MxL2(134*83)
Lineage(seq #)

S3.23 Fig.

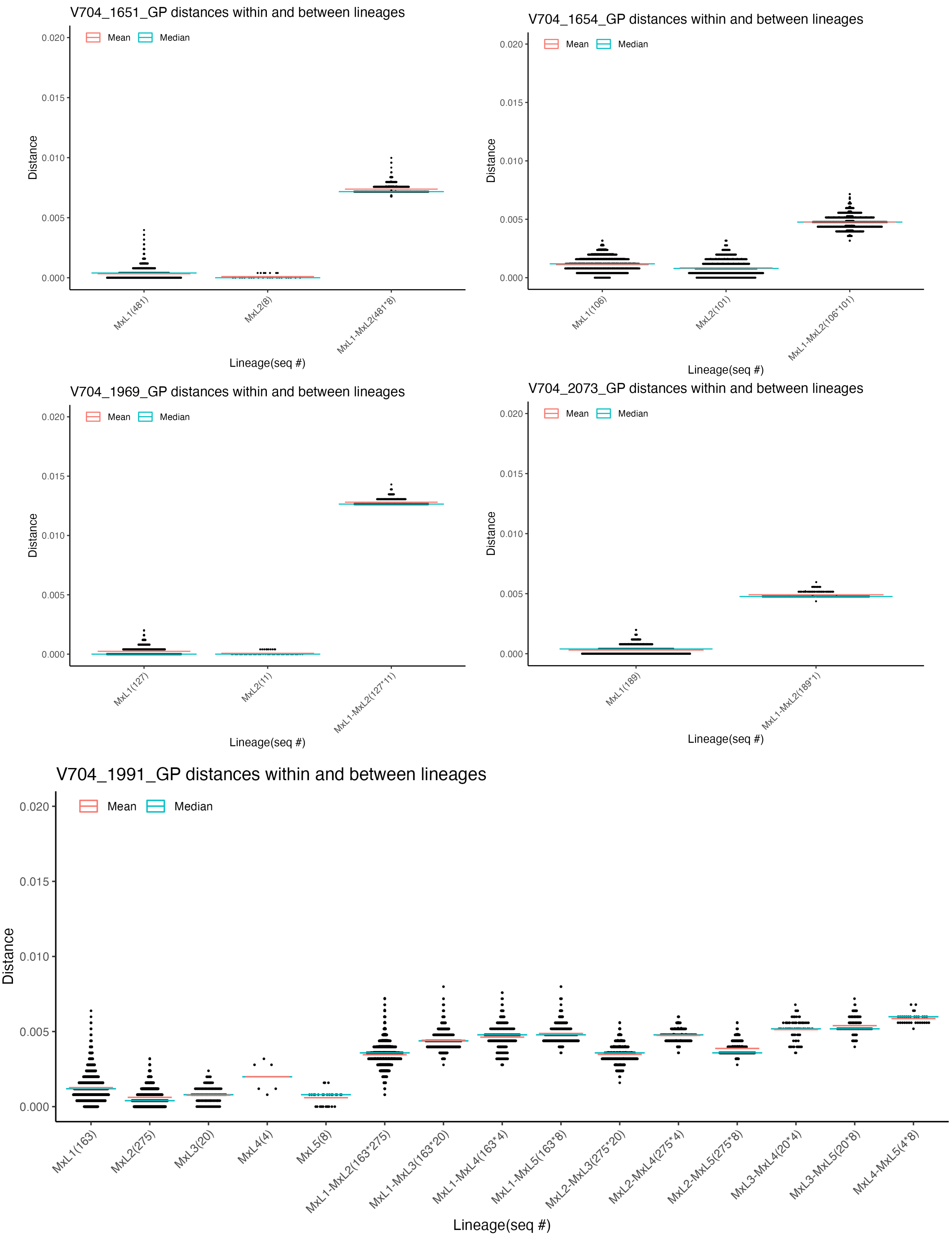

V704_1651_GP distances within and between lineages
Mean
Median
Distance
0.020
0.015
0.010
0.005
0.000
MxL1(481)
MxL2(8)
MxL1-MxL2(481*8)
Lineage(seq #)
V704_1654_GP distances within and between lineages
Mean
Median
Distance
0.020
0.015
0.010
0.005
0.000
MxL1(106)
MxL2(101)
MxL1-MxL2(106*101)
Lineage(seq #)
V704_1969_GP distances within and between lineages
Mean
Median
Distance
0.020
0.015
0.010
0.005
0.000
MxL1(127)
MxL2(11)
MxL1-MxL2(127*11)
Lineage(seq #)
V704_2073_GP distances within and between lineages
Mean
Median
Distance
0.020
0.015
0.010
0.005
0.000
MxL1(189)
MxL1-MxL2(189*1)
Lineage(seq #)
V704_1991_GP distances within and between lineages
Mean
Median
Distance
0.020
0.015
0.010
0.005
0.000
MxL1(163)
MxL2(275)
MxL3(20)
MxL4(4)
MxL5(8)
MxL1-MxL2(163*275)
MxL1-MxL3(163*20)
MxL1-MxL4(163*4)
MxL1-MxL5(163*8)
MxL2-MxL3(275*20)
MxL2-MxL4(275*4)
MxL2-MxL5(275*8)
MxL3-MxL4(20*4)
MxL3-MxL5(20*8)
MxL4-MxL5(4*8)
Lineage(seq #)

S3.24 Fig.

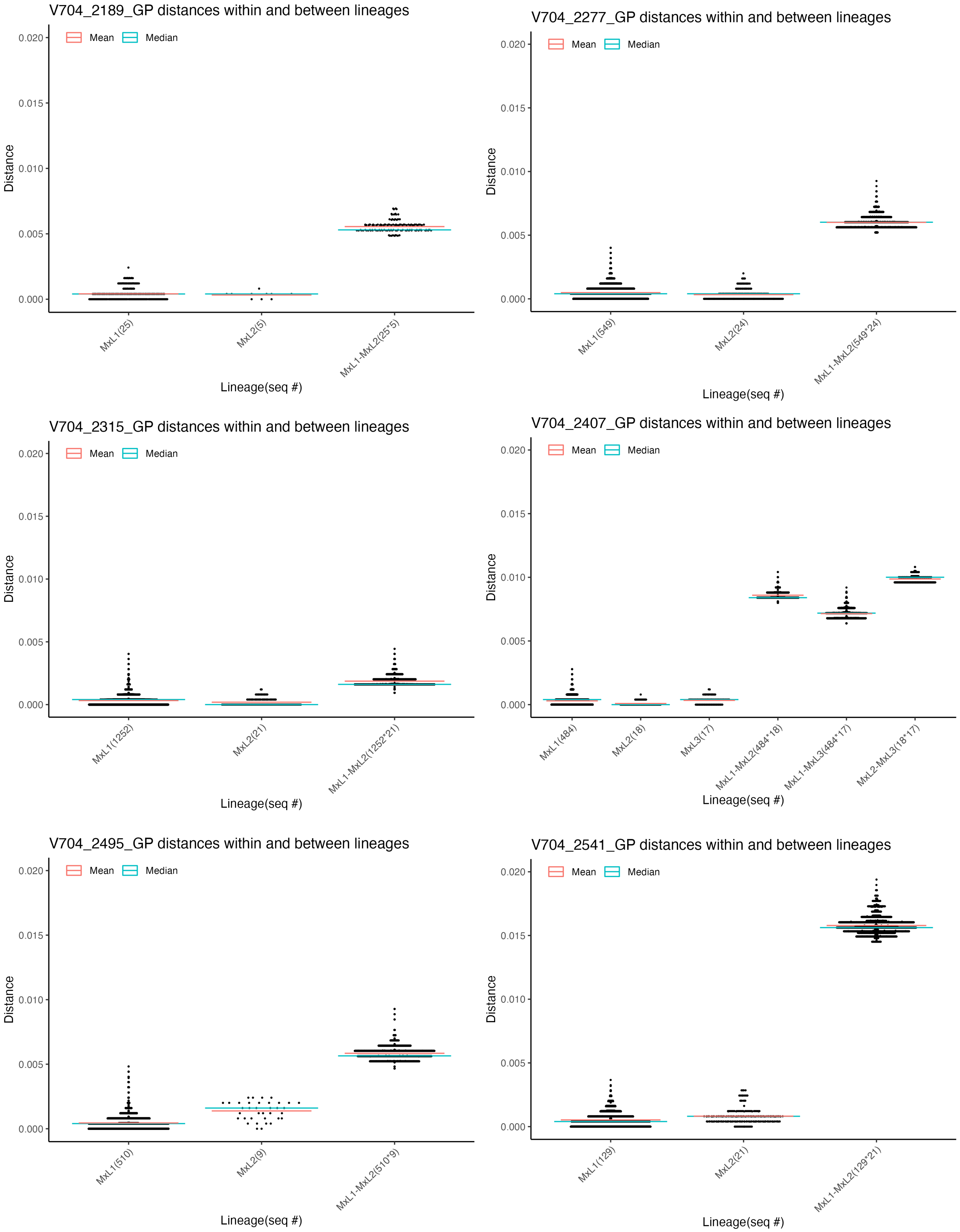
V704_2189_GP distances within and between lineages
Mean
Median
Distance
0.020
0.015
0.010
0.005
0.000
MxL1(25)
MxL2(5)
MxL1-MxL2(25*5)
Lineage(seq #)
V704_2277_GP distances within and between lineages
Mean
Median
Distance
0.020
0.015
0.010
0.005
0.000
MxL1(549)
MxL2(24)
MxL1-MxL2(549*24)
Lineage(seq #)
V704_2315_GP distances within and between lineages
Mean
Median
Distance
0.020
0.015
0.010
0.005
0.000
MxL1(1252)
MxL2(21)
MxL1-MxL2(1252*21)
Lineage(seq #)
V704_2407_GP distances within and between lineages
Mean
Median
Distance
0.020
0.015
0.010
0.005
0.000
MxL1(484)
MxL2(18)
MxL3(17)
MxL1-MxL2(484*18)
MxL1-MxL3(484*17)
MxL2-MxL3(18*17)
Lineage(seq #)
V704_2495_GP distances within and between lineages
Mean
Median
Distance
0.020
0.015
0.010
0.005
0.000
MxL1(510)
MxL2(9)
MxL1-MxL2(510*9)
Lineage(seq #)
V704_2541_GP distances within and between lineages
Mean
Median
Distance
0.020
0.015
0.010
0.005
0.000
MxL1(129)
MxL2(21)
MxL1-MxL2(129*21)
Lineage(seq #)

S3.25 Fig.

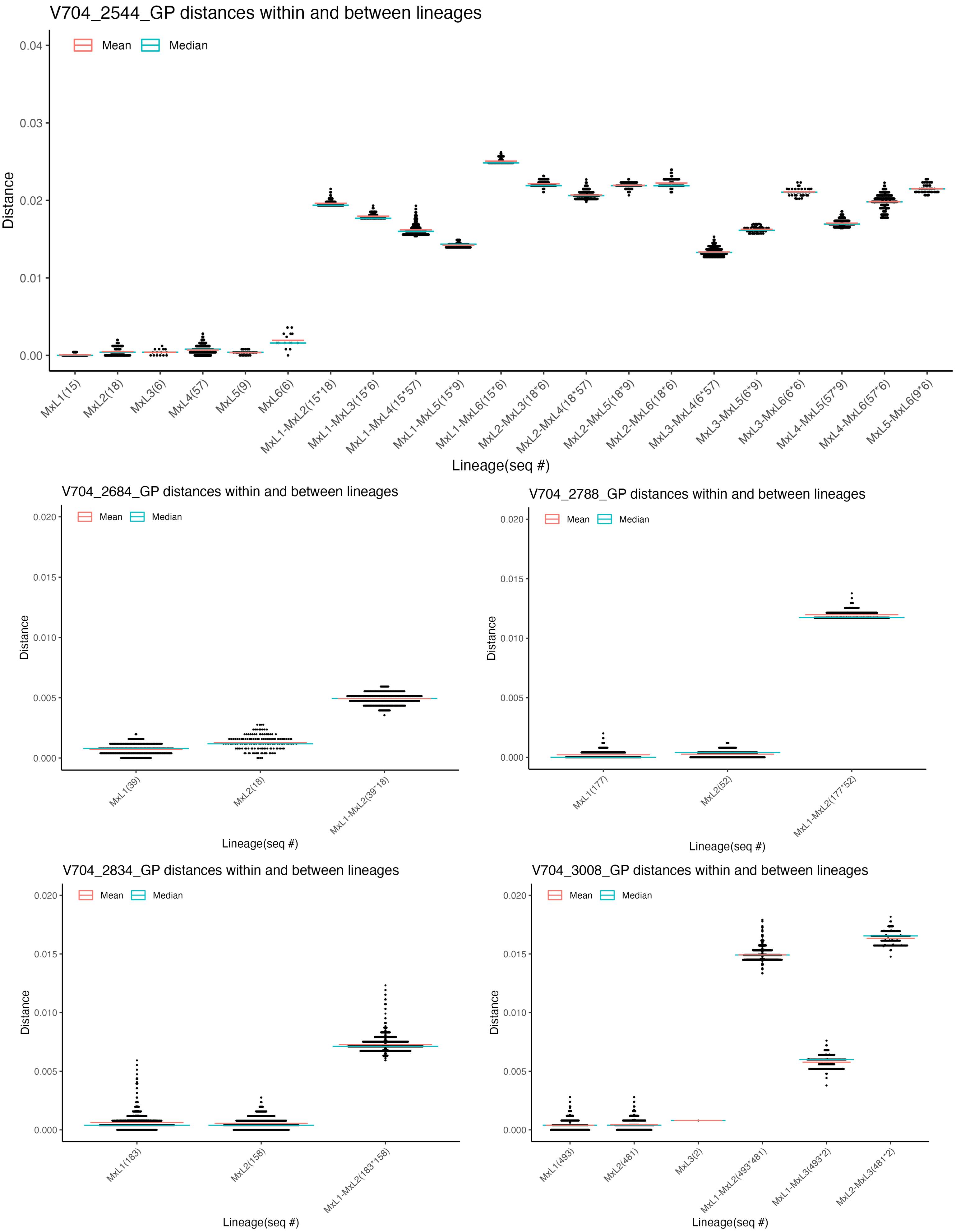
V704_2544_GP distances within and between lineages
Mean
Median
Distance
Lineage(seq #)
MxL1(15)
MxL2(18)
MxL3(6)
MxL4(57)
MxL5(9)
MxL6(6)
MxL1-MxL2(15*18)
MxL1-MxL3(15*6)
MxL1-MxL4(15*57)
MxL1-MxL5(15*9)
MxL1-MxL6(15*6)
MxL2-MxL3(18*6)
MxL2-MxL4(18*57)
MxL2-MxL5(18*9)
MxL2-MxL6(18*6)
MxL3-MxL4(6*57)
MxL3-MxL5(6*9)
MxL3-MxL6(6*6)
MxL4-MxL5(57*9)
MxL4-MxL6(57*6)
MxL5-MxL6(9*6)
V704_2684_GP distances within and between lineages
MxL1(39)
MxL2(18)
MxL1-MxL2(39*18)
V704_2788_GP distances within and between lineages
MxL1(177)
MxL2(52)
MxL1-MxL2(177*52)
V704_2834_GP distances within and between lineages
MxL1(183)
MxL2(158)
MxL1-MxL2(183*158)
V704_3008_GP distances within and between lineages
MxL1(493)
MxL2(481)
MxL3(2)
MxL1-MxL2(493*481)
MxL1-MxL3(493*2)
MxL2-MxL3(481*2)

S3.26 Fig.

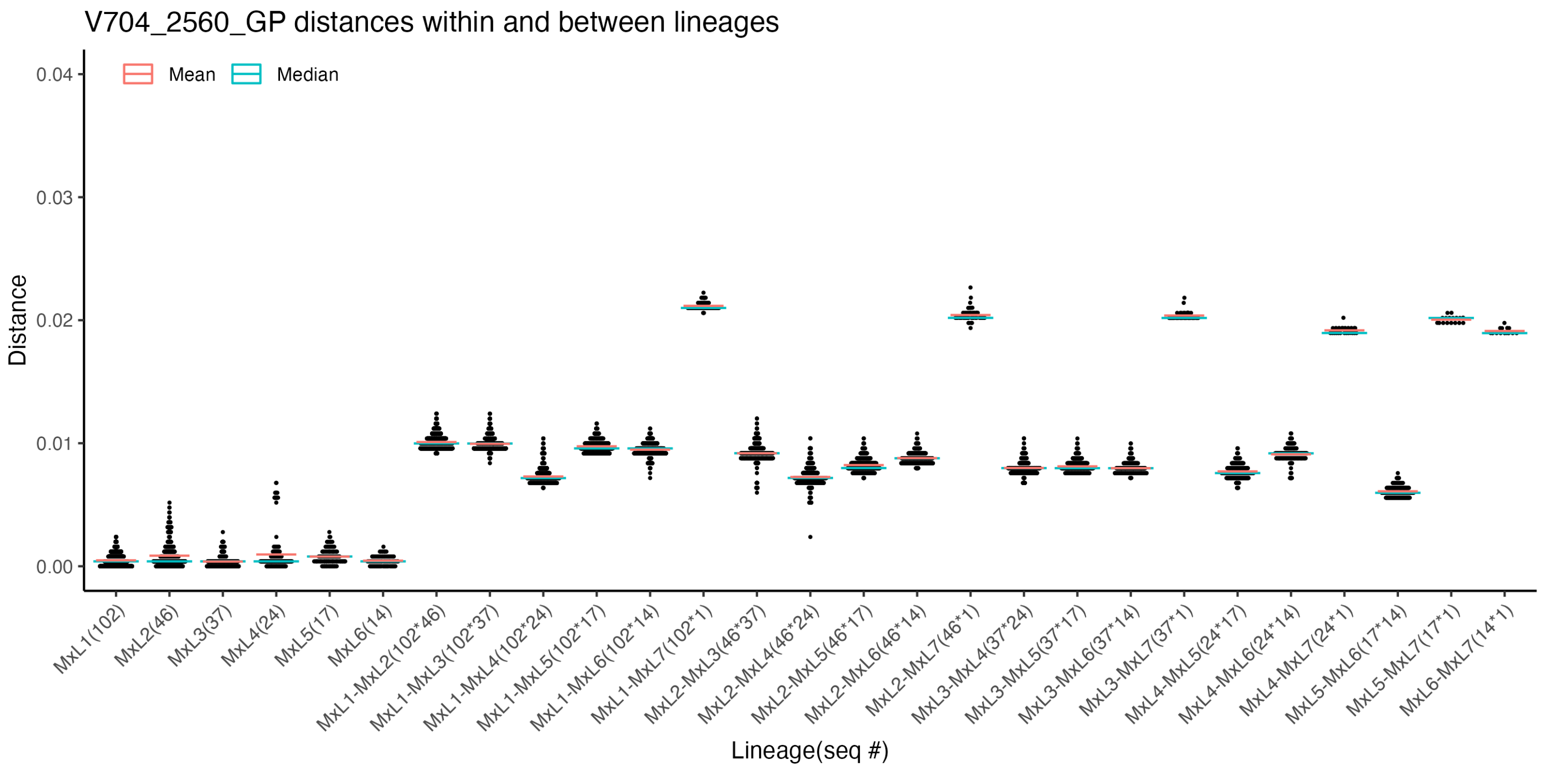
V704_2560_GP distances within and between lineages
Mean
Median
Distance
0.04
0.03
0.02
0.01
0.00
MxL1(102)
MxL2(46)
MxL3(37)
MxL4(24)
MxL5(17)
MxL6(14)
MxL1-MxL2(102*46)
MxL1-MxL3(102*37)
MxL1-MxL4(102*24)
MxL1-MxL5(102*17)
MxL1-MxL6(102*14)
MxL1-MxL7(102*1)
MxL2-MxL3(46*37)
MxL2-MxL4(46*24)
MxL2-MxL5(46*17)
MxL2-MxL6(46*14)
MxL2-MxL7(46*1)
MxL3-MxL4(37*24)
MxL3-MxL5(37*17)
MxL3-MxL6(37*14)
MxL3-MxL7(37*1)
MxL4-MxL5(24*17)
MxL4-MxL6(24*14)
MxL4-MxL7(24*1)
MxL5-MxL6(17*14)
MxL5-MxL7(17*1)
MxL6-MxL7(14*1)
Lineage(seq #)

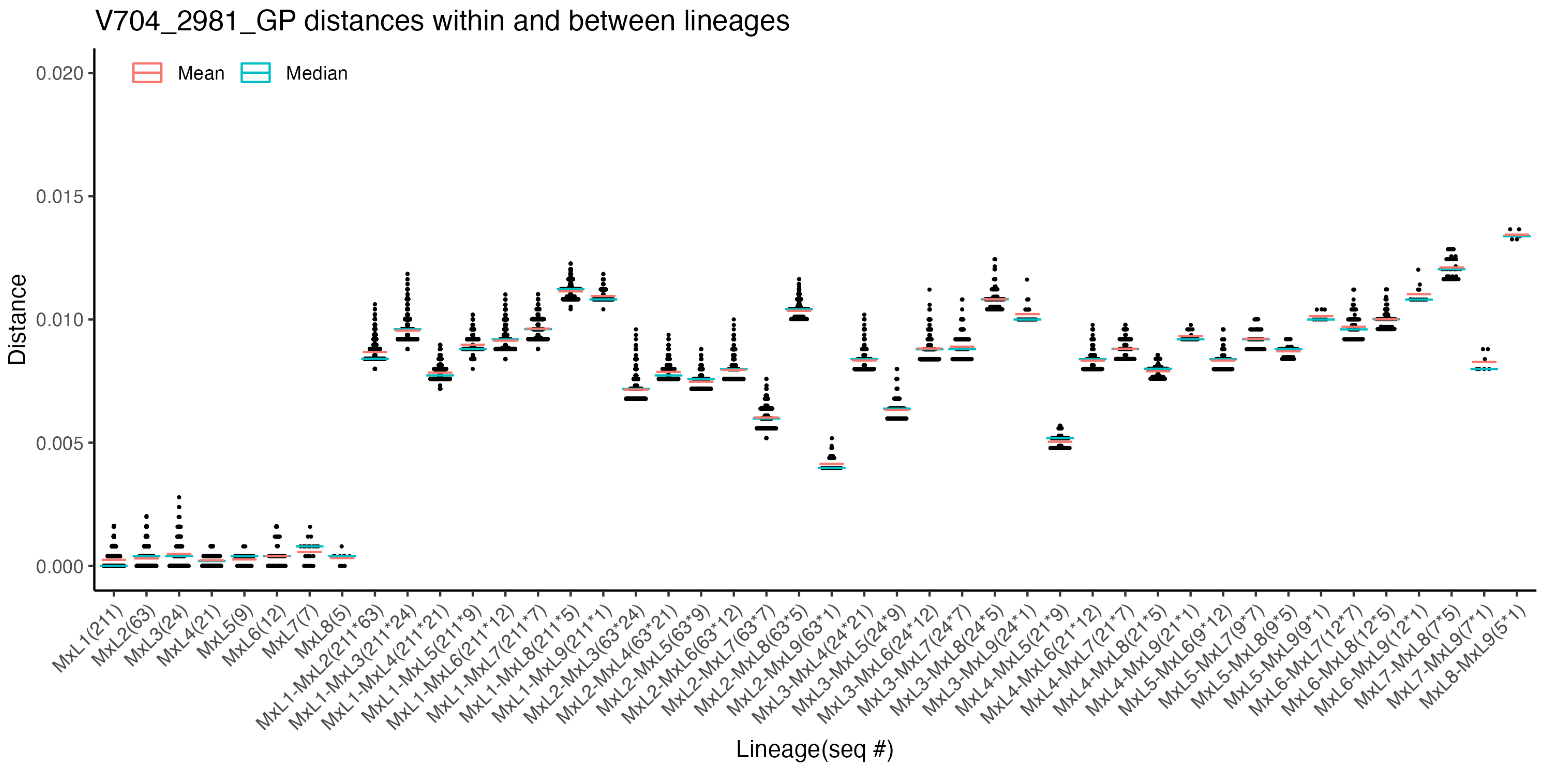
V704_2981_GP distances within and between lineages
Mean
Median
Distance
0.020
0.015
0.010
0.005
0.000
MxL1(211)
MxL2(63)
MxL3(24)
MxL4(21)
MxL5(9)
MxL6(12)
MxL7(7)
MxL8(5)
MxL1-MxL2(211*63)
MxL1-MxL3(211*24)
MxL1-MxL4(211*21)
MxL1-MxL5(211*9)
MxL1-MxL6(211*12)
MxL1-MxL7(211*7)
MxL1-MxL8(211*5)
MxL1-MxL9(211*1)
MxL2-MxL3(63*24)
MxL2-MxL4(63*21)
MxL2-MxL5(63*9)
MxL2-MxL6(63*12)
MxL2-MxL7(63*7)
MxL2-MxL8(63*5)
MxL2-MxL9(63*1)
MxL3-MxL4(24*21)
MxL3-MxL5(24*9)
MxL3-MxL6(24*12)
MxL3-MxL7(24*7)
MxL3-MxL8(24*5)
MxL3-MxL9(24*1)
MxL4-MxL5(21*9)
MxL4-MxL6(21*12)
MxL4-MxL7(21*7)
MxL4-MxL8(21*5)
MxL4-MxL9(21*1)
MxL5-MxL6(9*12)
MxL5-MxL7(9*7)
MxL5-MxL8(9*5)
MxL5-MxL9(9*1)
MxL6-MxL7(12*7)
MxL6-MxL8(12*5)
MxL6-MxL9(12*1)
MxL7-MxL8(7*5)
MxL7-MxL9(7*1)
MxL8-MxL9(5*1)
Lineage(seq #)

S3.27 Fig.

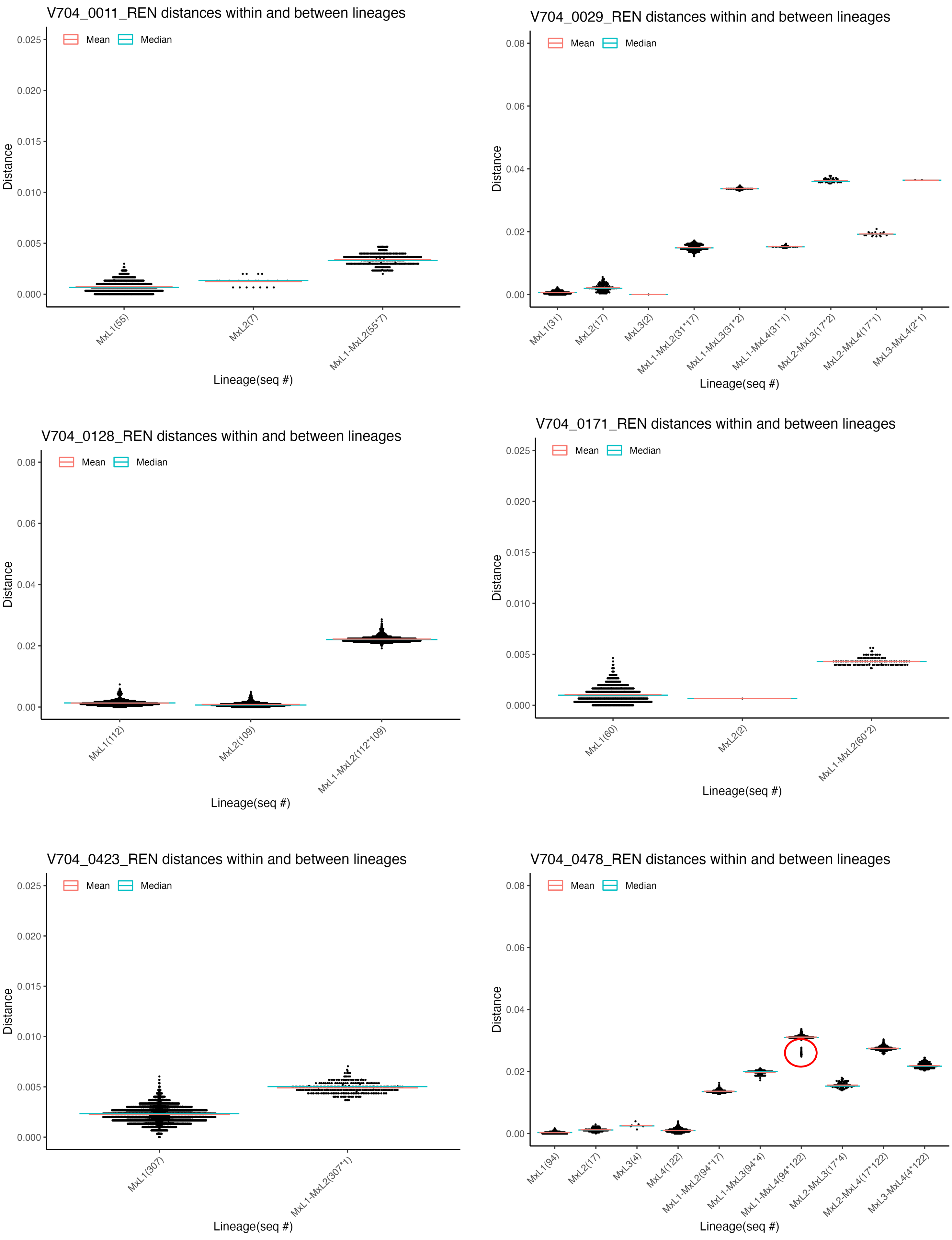
V704_0011_REN distances within and between lineages
Mean
Median
Distance
0.025
0.020
0.015
0.010
0.005
0.000
MxL1(55)
MxL2(7)
MxL1-MxL2(55*7)
Lineage(seq #)
V704_0029_REN distances within and between lineages
Mean
Median
Distance
0.08
0.06
0.04
0.02
0.00
MxL1(31)
MxL2(17)
MxL3(2)
MxL1-MxL2(31*17)
MxL1-MxL3(31*2)
MxL1-MxL4(31*1)
MxL2-MxL3(17*2)
MxL2-MxL4(17*1)
MxL3-MxL4(2*1)
Lineage(seq #)
V704_0128_REN distances within and between lineages
Mean
Median
Distance
0.08
0.06
0.04
0.02
0.00
MxL1(112)
MxL2(109)
MxL1-MxL2(112*109)
Lineage(seq #)
V704_0171_REN distances within and between lineages
Mean
Median
Distance
0.025
0.020
0.015
0.010
0.005
0.000
MxL1(60)
MxL2(2)
MxL1-MxL2(60*2)
Lineage(seq #)
V704_0423_REN distances within and between lineages
Mean
Median
Distance
0.025
0.020
0.015
0.010
0.005
0.000
MxL1(307)
MxL1-MxL2(307*1)
Lineage(seq #)
V704_0478_REN distances within and between lineages
Mean
Median
Distance
0.08
0.06
0.04
0.02
0.00
MxL1(94)
MxL2(17)
MxL3(4)
MxL4(122)
MxL1-MxL2(94*17)
MxL1-MxL3(94*4)
MxL1-MxL4(94*122)
MxL2-MxL3(17*4)
MxL2-MxL4(17*122)
MxL3-MxL4(4*122)
Lineage(seq #)

S3.28 Fig.

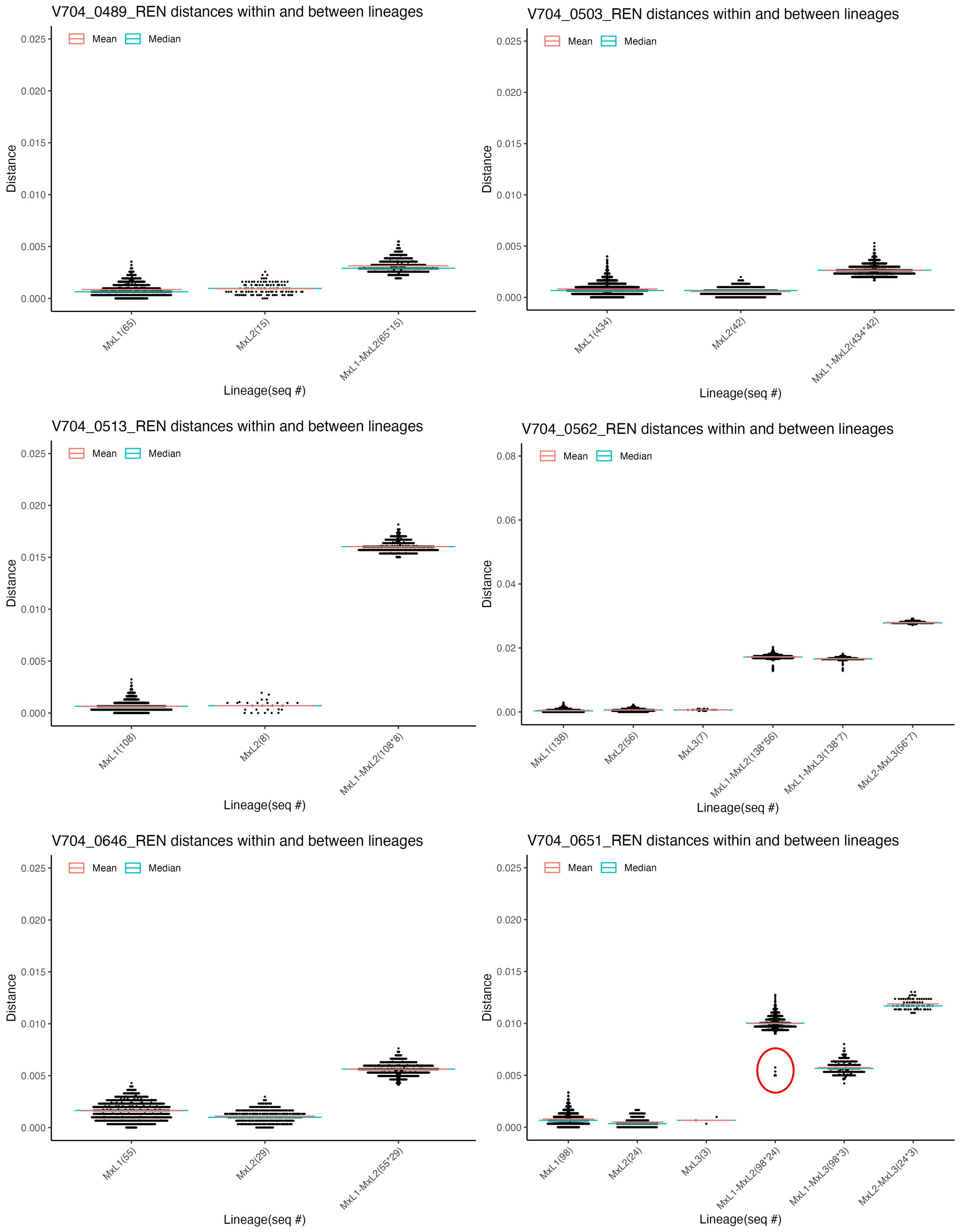
V704_0489_REN distances within and between lineages
Mean
Median
Distance
Lineage(seq #)
MxL1(65)
MxL2(15)
MxL1-MxL2(65*15)
V704_0503_REN distances within and between lineages
MxL1(434)
MxL2(42)
MxL1-MxL2(434*42)
V704_0513_REN distances within and between lineages
MxL1(108)
MxL2(8)
MxL1-MxL2(108*8)
V704_0562_REN distances within and between lineages
MxL1(138)
MxL2(56)
MxL3(7)
MxL1-MxL2(138*56)
MxL1-MxL3(138*7)
MxL2-MxL3(56*7)
V704_0646_REN distances within and between lineages
MxL1(55)
MxL2(29)
MxL1-MxL2(55*29)
V704_0651_REN distances within and between lineages
MxL1(98)
MxL2(24)
MxL3(3)
MxL1-MxL2(98*24)
MxL1-MxL3(98*3)
MxL2-MxL3(24*3)

S3.29 Fig.

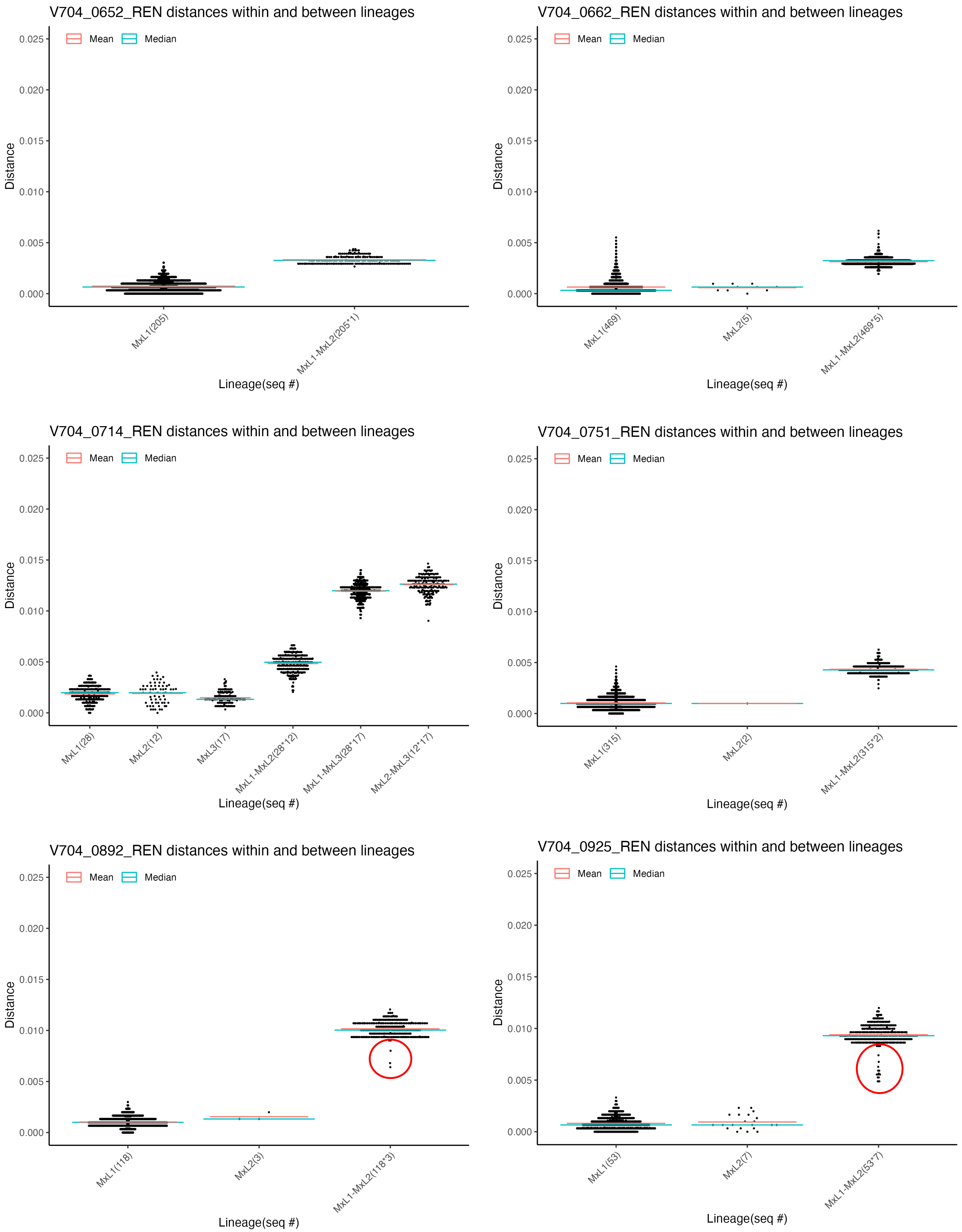

V704_0652_REN distances within and between lineages
Mean
Median
Distance
0.025
0.020
0.015
0.010
0.005
0.000
MxL1(205)
MxL1-MxL2(205*1)
Lineage(seq #)
V704_0662_REN distances within and between lineages
Mean
Median
Distance
0.025
0.020
0.015
0.010
0.005
0.000
MxL1(469)
MxL2(5)
MxL1-MxL2(469*5)
Lineage(seq #)
V704_0714_REN distances within and between lineages
Mean
Median
Distance
0.025
0.020
0.015
0.010
0.005
0.000
MxL1(28)
MxL2(12)
MxL3(17)
MxL1-MxL2(28*12)
MxL1-MxL3(28*17)
MxL2-MxL3(12*17)
Lineage(seq #)
V704_0751_REN distances within and between lineages
Mean
Median
Distance
0.025
0.020
0.015
0.010
0.005
0.000
MxL1(315)
MxL2(2)
MxL1-MxL2(315*2)
Lineage(seq #)
V704_0892_REN distances within and between lineages
Mean
Median
Distance
0.025
0.020
0.015
0.010
0.005
0.000
MxL1(118)
MxL2(3)
MxL1-MxL2(118*3)
Lineage(seq #)
V704_0925_REN distances within and between lineages
Mean
Median
Distance
0.025
0.020
0.015
0.010
0.005
0.000
MxL1(53)
MxL2(7)
MxL1-MxL2(53*7)
Lineage(seq #)

S3.30 Fig.

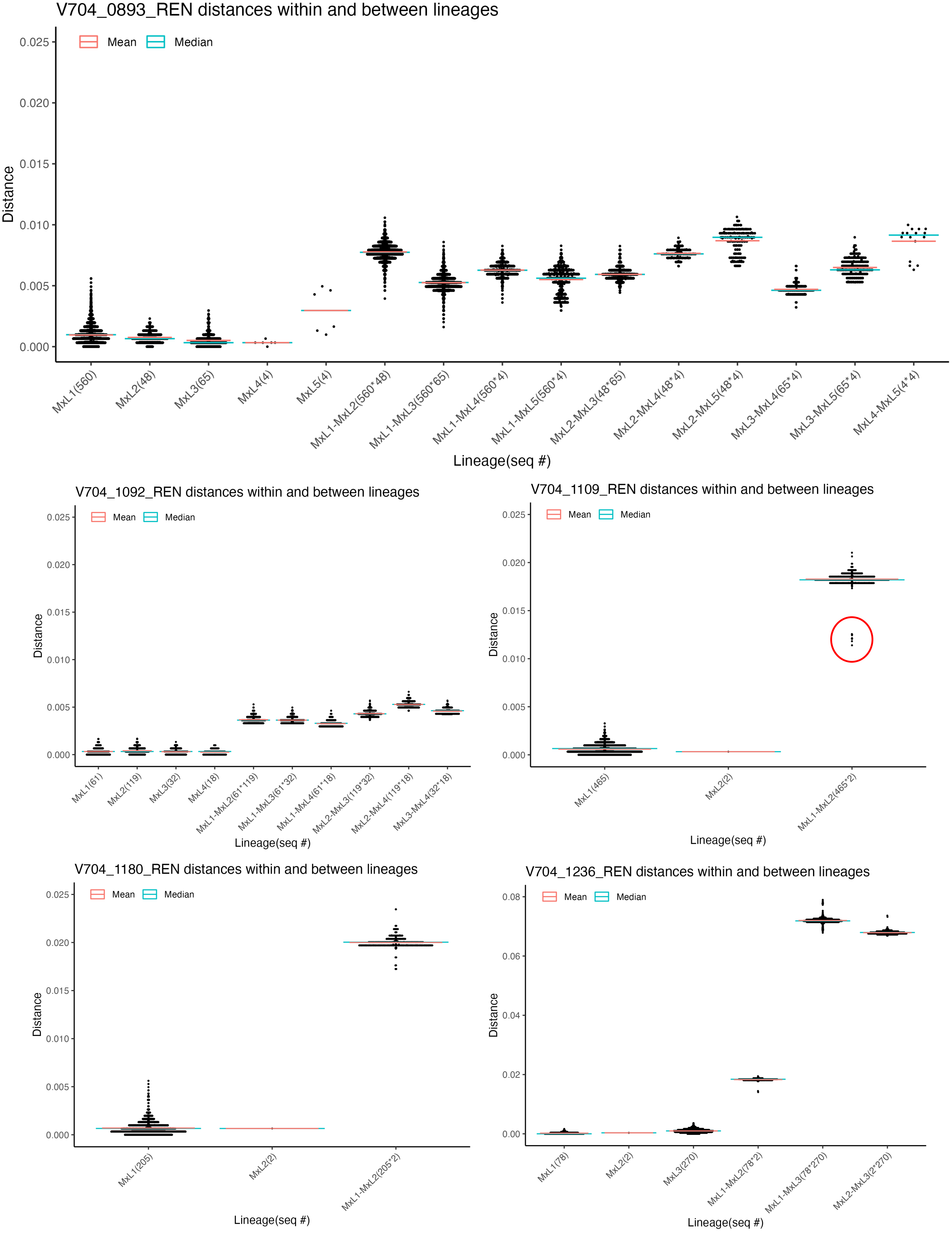
V704_0893_REN distances within and between lineages
Mean
Median
Distance
0.025
0.020
0.015
0.010
0.005
0.000
MxL1(560)
MxL2(48)
MxL3(65)
MxL4(4)
MxL5(4)
MxL1-MxL2(560*48)
MxL1-MxL3(560*65)
MxL1-MxL4(560*4)
MxL1-MxL5(560*4)
MxL2-MxL3(48*65)
MxL2-MxL4(48*4)
MxL2-MxL5(48*4)
MxL3-MxL4(65*4)
MxL3-MxL5(65*4)
MxL4-MxL5(4*4)
Lineage(seq #)
V704_1092_REN distances within and between lineages
Mean
Median
Distance
0.025
0.020
0.015
0.010
0.005
0.000
MxL1(61)
MxL2(119)
MxL3(32)
MxL4(18)
MxL1-MxL2(61*119)
MxL1-MxL3(61*32)
MxL1-MxL4(61*18)
MxL2-MxL3(119*32)
MxL2-MxL4(119*18)
MxL3-MxL4(32*18)
Lineage(seq #)
V704_1109_REN distances within and between lineages
Mean
Median
Distance
0.025
0.020
0.015
0.010
0.005
0.000
MxL1(465)
MxL2(2)
MxL1-MxL2(465*2)
Lineage(seq #)
V704_1180_REN distances within and between lineages
Mean
Median
Distance
0.025
0.020
0.015
0.010
0.005
0.000
MxL1(205)
MxL2(2)
MxL1-MxL2(205*2)
Lineage(seq #)
V704_1236_REN distances within and between lineages
Mean
Median
Distance
0.08
0.06
0.04
0.02
0.00
MxL1(78)
MxL2(2)
MxL3(270)
MxL1-MxL2(78*2)
MxL1-MxL3(78*270)
MxL2-MxL3(2*270)
Lineage(seq #)

S3.31 Fig.

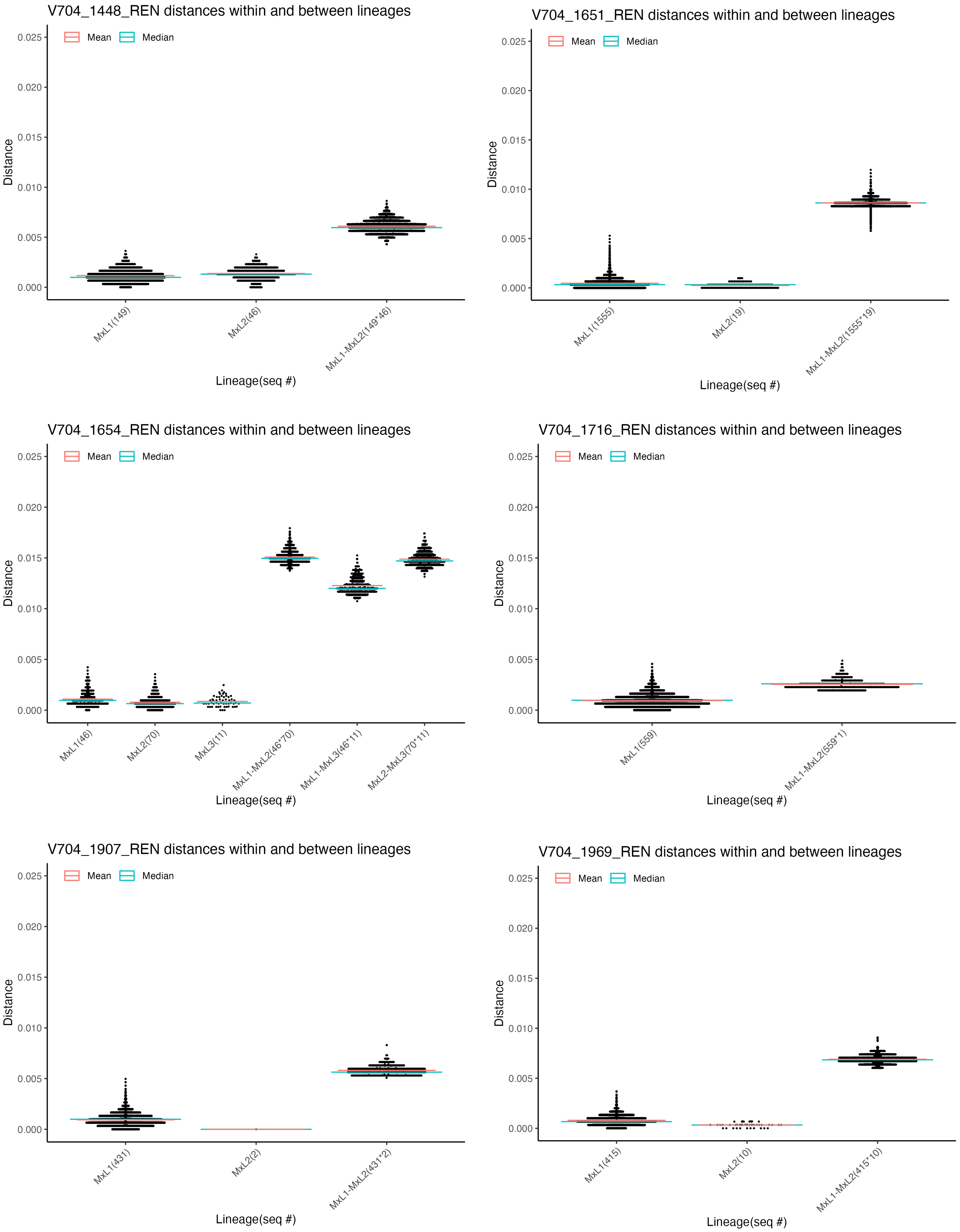
V704_1448_REN distances within and between lineages
Mean
Median
Distance
0.000
0.005
0.010
0.015
0.020
0.025
MxL1(149)
MxL2(46)
MxL1-MxL2(149*46)
Lineage(seq #)
V704_1651_REN distances within and between lineages
Mean
Median
Distance
MxL1(1555)
MxL2(19)
MxL1-MxL2(1555*19)
Lineage(seq #)
V704_1654_REN distances within and between lineages
Mean
Median
Distance
MxL1(46)
MxL2(70)
MxL3(11)
MxL1-MxL2(46*70)
MxL1-MxL3(46*11)
MxL2-MxL3(70*11)
Lineage(seq #)
V704_1716_REN distances within and between lineages
Mean
Median
Distance
MxL1(559)
MxL1-MxL2(559*1)
Lineage(seq #)
V704_1907_REN distances within and between lineages
Mean
Median
Distance
MxL1(431)
MxL2(2)
MxL1-MxL2(431*2)
Lineage(seq #)
V704_1969_REN distances within and between lineages
Mean
Median
Distance
MxL1(415)
MxL2(10)
MxL1-MxL2(415*10)
Lineage(seq #)

S3.32 Fig.

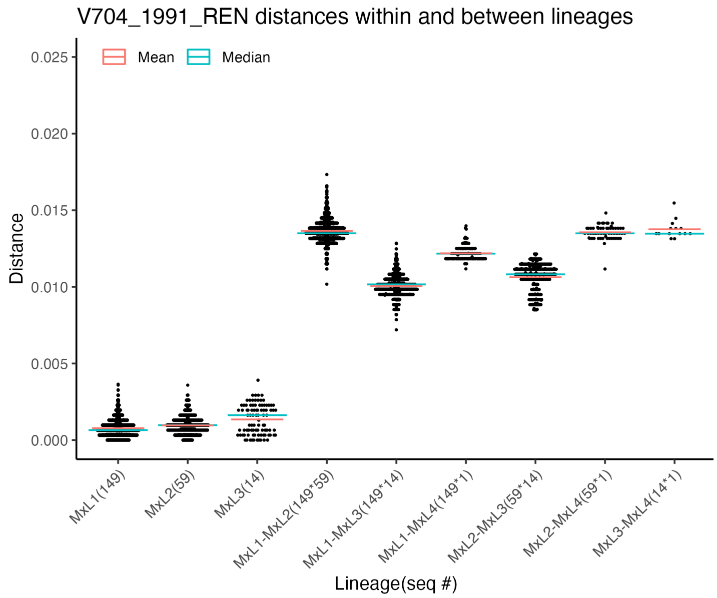
V704_1991_REN distances within and between lineages
Mean
Median
Distance
0.025
0.020
0.015
0.010
0.005
0.000
MxL1(149)
MxL2(59)
MxL3(14)
MxL1-MxL2(149*59)
MxL1-MxL3(149*14)
MxL1-MxL4(149*1)
MxL2-MxL3(59*14)
MxL2-MxL4(59*1)
MxL3-MxL4(14*1)
Lineage(seq #)

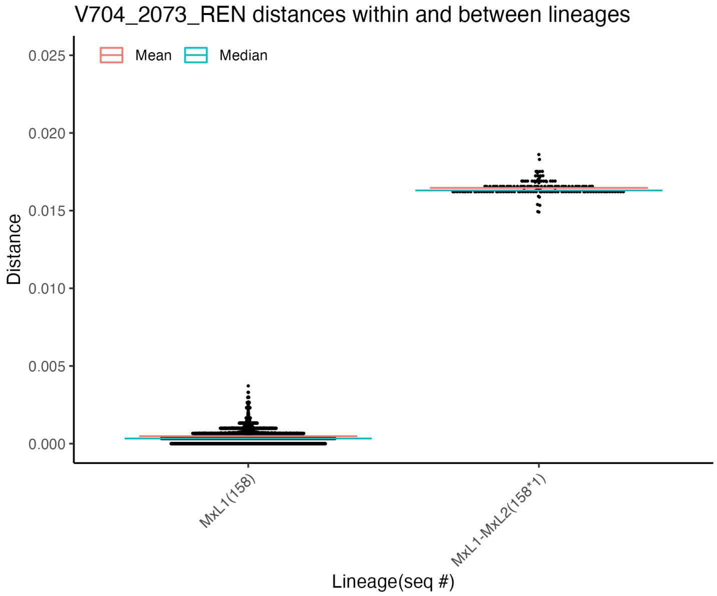
V704_2073_REN distances within and between lineages
Mean
Median
Distance
0.025
0.020
0.015
0.010
0.005
0.000
MxL1(158)
MxL1-MxL2(158*1)
Lineage(seq #)

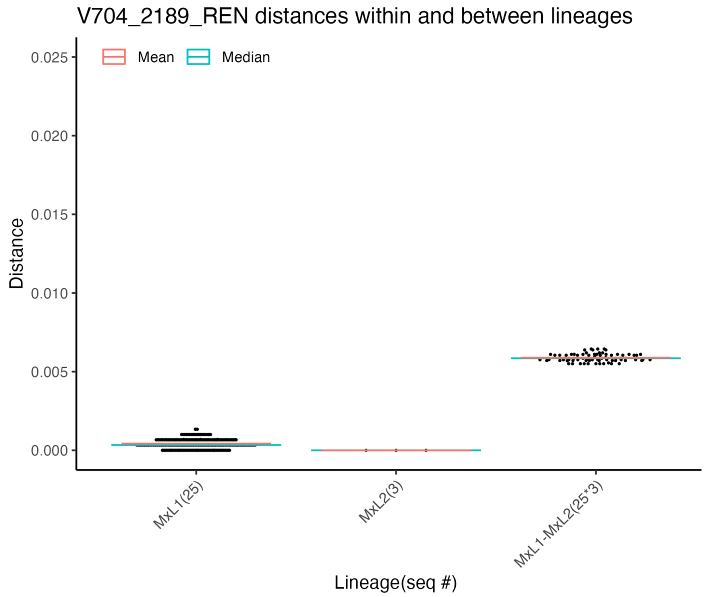
V704_2189_REN distances within and between lineages
Mean
Median
Distance
0.025
0.020
0.015
0.010
0.005
0.000
MxL1(25)
MxL2(3)
MxL1-MxL2(25*3)
Lineage(seq #)

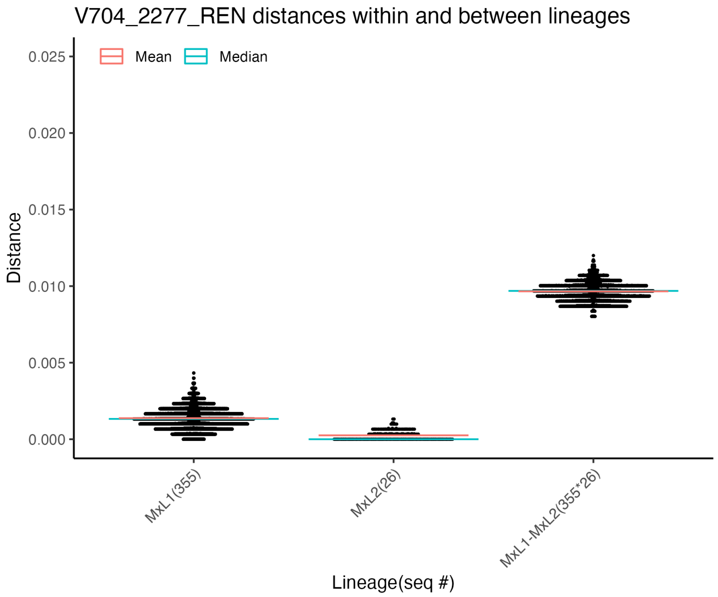
V704_2277_REN distances within and between lineages
Mean
Median
Distance
0.025
0.020
0.015
0.010
0.005
0.000
MxL1(355)
MxL2(26)
MxL1-MxL2(355*26)
Lineage(seq #)

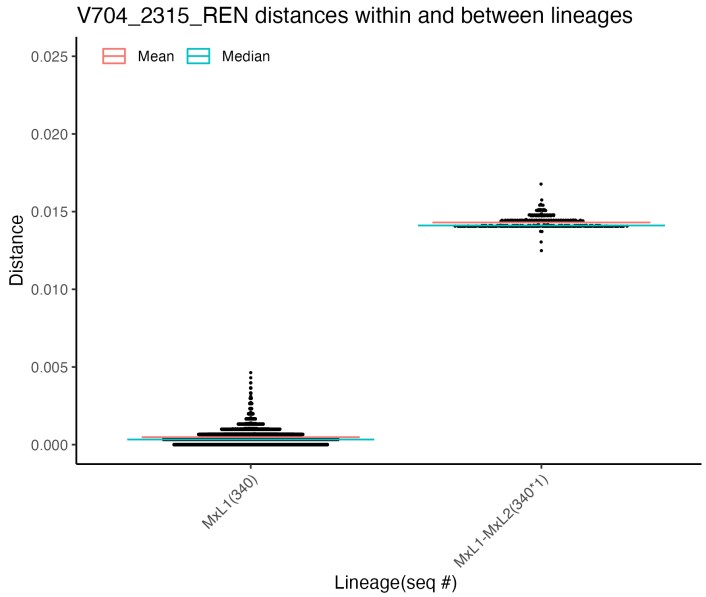
V704_2315_REN distances within and between lineages
Mean
Median
Distance
0.025
0.020
0.015
0.010
0.005
0.000
MxL1(340)
MxL1-MxL2(340*1)
Lineage(seq #)

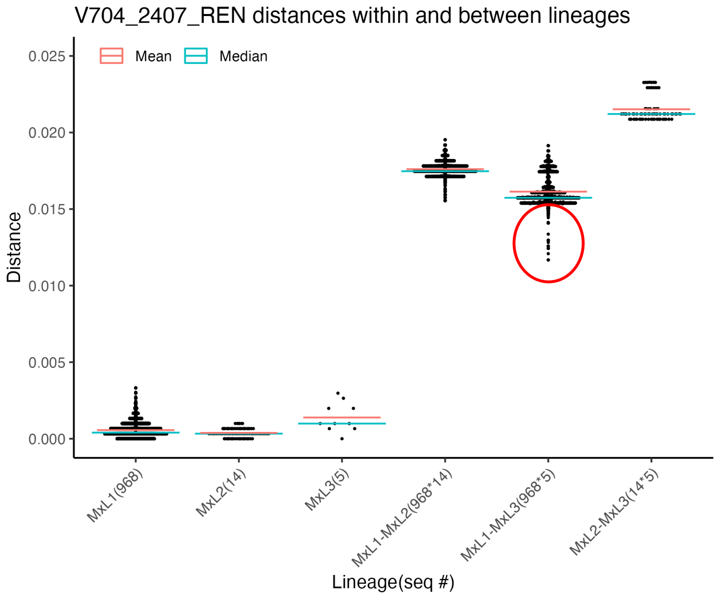
V704_2407_REN distances within and between lineages
Mean
Median
Distance
0.025
0.020
0.015
0.010
0.005
0.000
MxL1(968)
MxL2(14)
MxL3(5)
MxL1-MxL2(968*14)
MxL1-MxL3(968*5)
MxL2-MxL3(14*5)
Lineage(seq #)

S3.33 Fig.

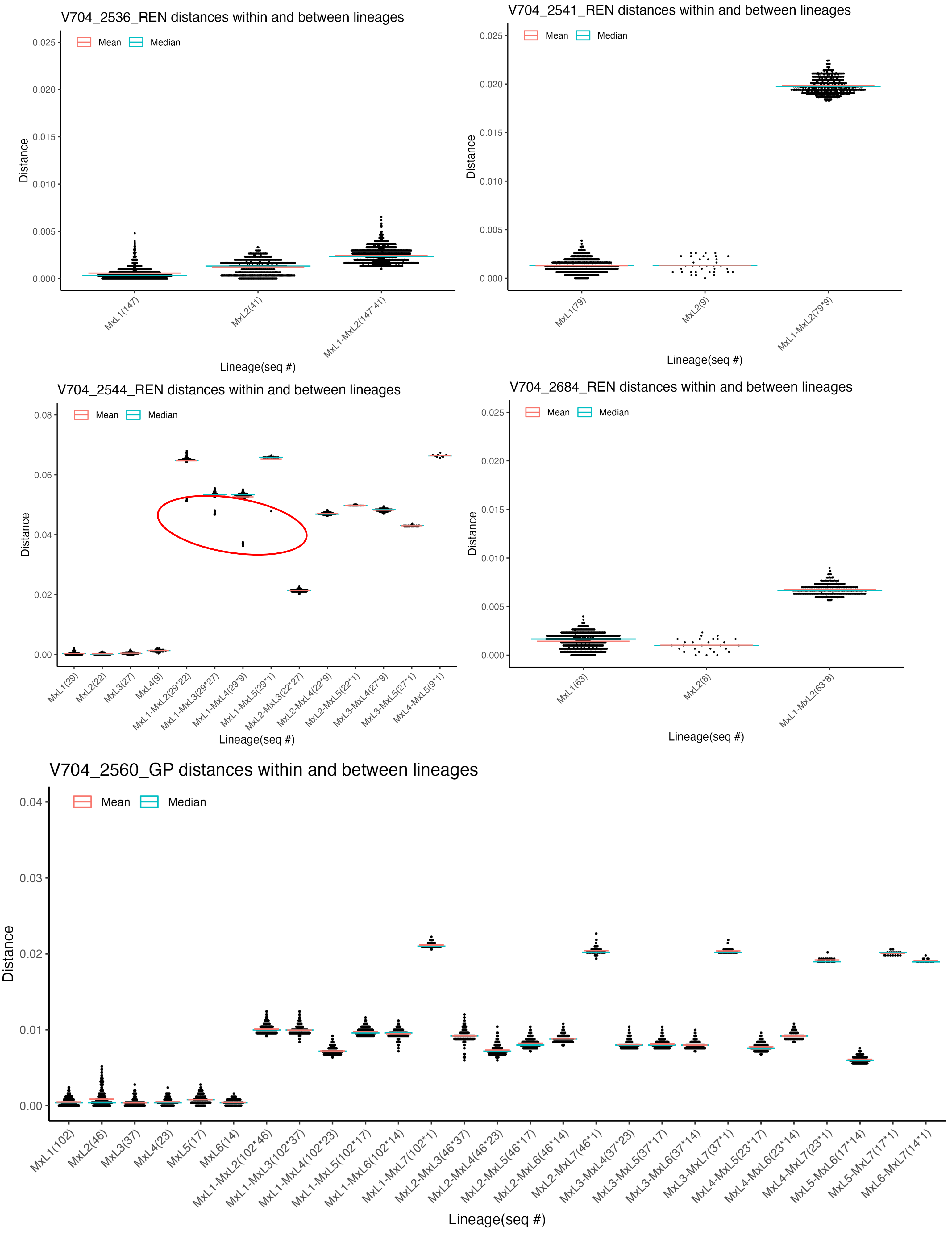
V704_2536_REN distances within and between lineages
Mean
Median
Distance
0.025
0.020
0.015
0.010
0.005
0.000
MxL1(147)
MxL2(41)
MxL1-MxL2(147*41)
Lineage(seq #)
V704_2541_REN distances within and between lineages
Mean
Median
Distance
0.025
0.020
0.015
0.010
0.005
0.000
MxL1(79)
MxL2(9)
MxL1-MxL2(79*9)
Lineage(seq #)
V704_2544_REN distances within and between lineages
Mean
Median
Distance
0.08
0.06
0.04
0.02
0.00
MxL1(29)
MxL2(22)
MxL3(27)
MxL4(9)
MxL1-MxL2(29*22)
MxL1-MxL3(29*27)
MxL1-MxL4(29*9)
MxL1-MxL5(29*1)
MxL2-MxL3(22*27)
MxL2-MxL4(22*9)
MxL2-MxL5(22*1)
MxL3-MxL4(27*9)
MxL3-MxL5(27*1)
MxL4-MxL5(9*1)
Lineage(seq #)
V704_2684_REN distances within and between lineages
Mean
Median
Distance
0.025
0.020
0.015
0.010
0.005
0.000
MxL1(63)
MxL2(8)
MxL1-MxL2(63*8)
Lineage(seq #)
V704_2560_GP distances within and between lineages
Mean
Median
Distance
0.04
0.03
0.02
0.01
0.00
MxL1(102)
MxL2(46)
MxL3(37)
MxL4(23)
MxL5(17)
MxL6(14)
MxL1-MxL2(102*46)
MxL1-MxL3(102*37)
MxL1-MxL4(102*23)
MxL1-MxL5(102*17)
MxL1-MxL6(102*14)
MxL1-MxL7(102*1)
MxL2-MxL3(46*37)
MxL2-MxL4(46*23)
MxL2-MxL5(46*17)
MxL2-MxL6(46*14)
MxL2-MxL7(46*1)
MxL3-MxL4(37*23)
MxL3-MxL5(37*17)
MxL3-MxL6(37*14)
MxL3-MxL7(37*1)
MxL4-MxL5(23*17)
MxL4-MxL6(23*14)
MxL4-MxL7(23*1)
MxL5-MxL6(17*14)
MxL5-MxL7(17*1)
MxL6-MxL7(14*1)
Lineage(seq #)

S3.34 Fig.

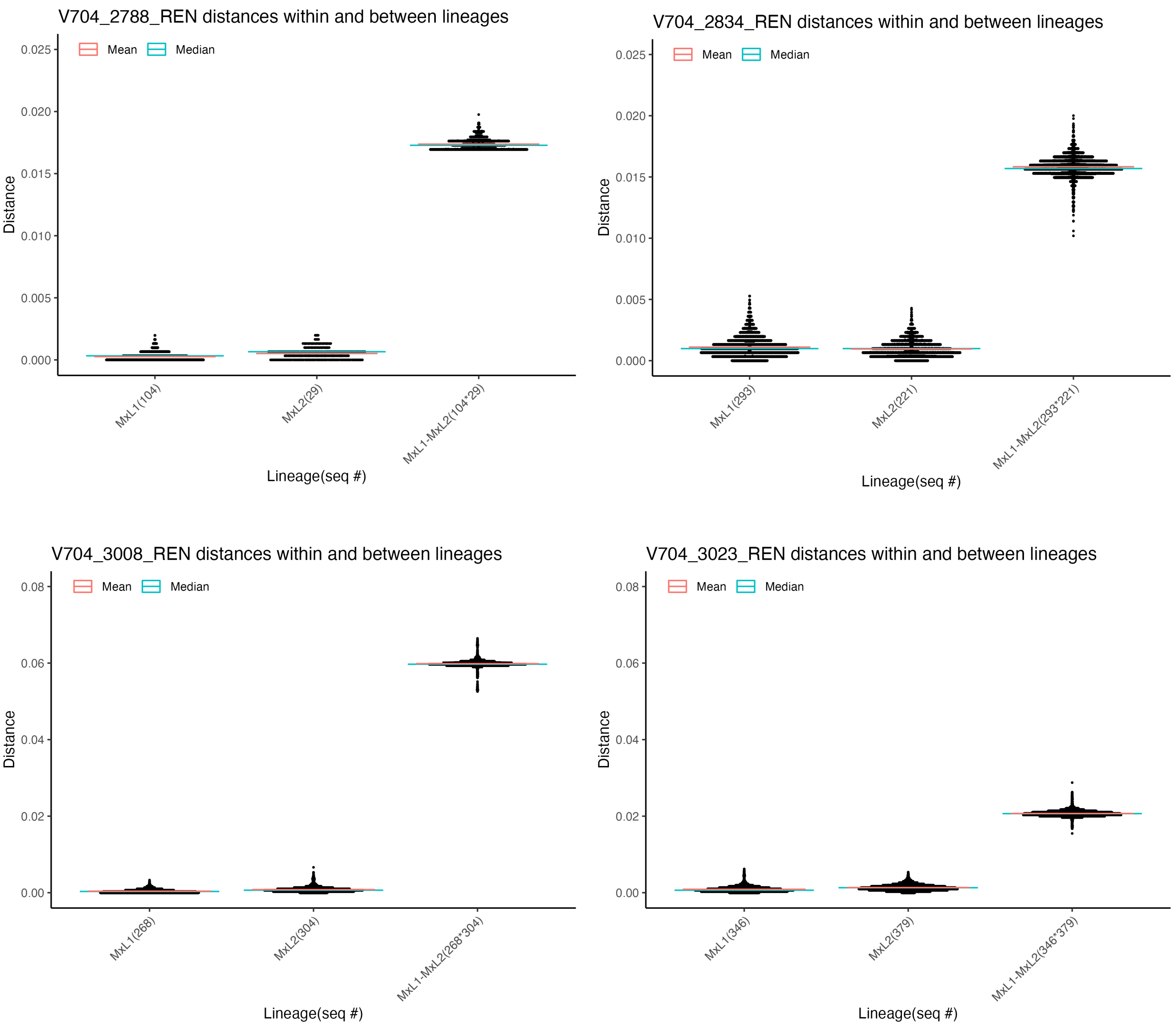
V704_2788_REN distances within and between lineages
Mean
Median
Distance
0.025
0.020
0.015
0.010
0.005
0.000
MxL1(104)
MxL2(29)
MxL1-MxL2(104*29)
Lineage(seq #)
V704_2834_REN distances within and between lineages
Mean
Median
Distance
0.025
0.020
0.015
0.010
0.005
0.000
MxL1(293)
MxL2(221)
MxL1-MxL2(293*221)
Lineage(seq #)
V704_3008_REN distances within and between lineages
Mean
Median
Distance
0.08
0.06
0.04
0.02
0.00
MxL1(268)
MxL2(304)
MxL1-MxL2(268*304)
Lineage(seq #)
V704_3023_REN distances within and between lineages
Mean
Median
Distance
0.08
0.06
0.04
0.02
0.00
MxL1(346)
MxL2(379)
MxL1-MxL2(346*379)
Lineage(seq #)

S3.35 Fig.

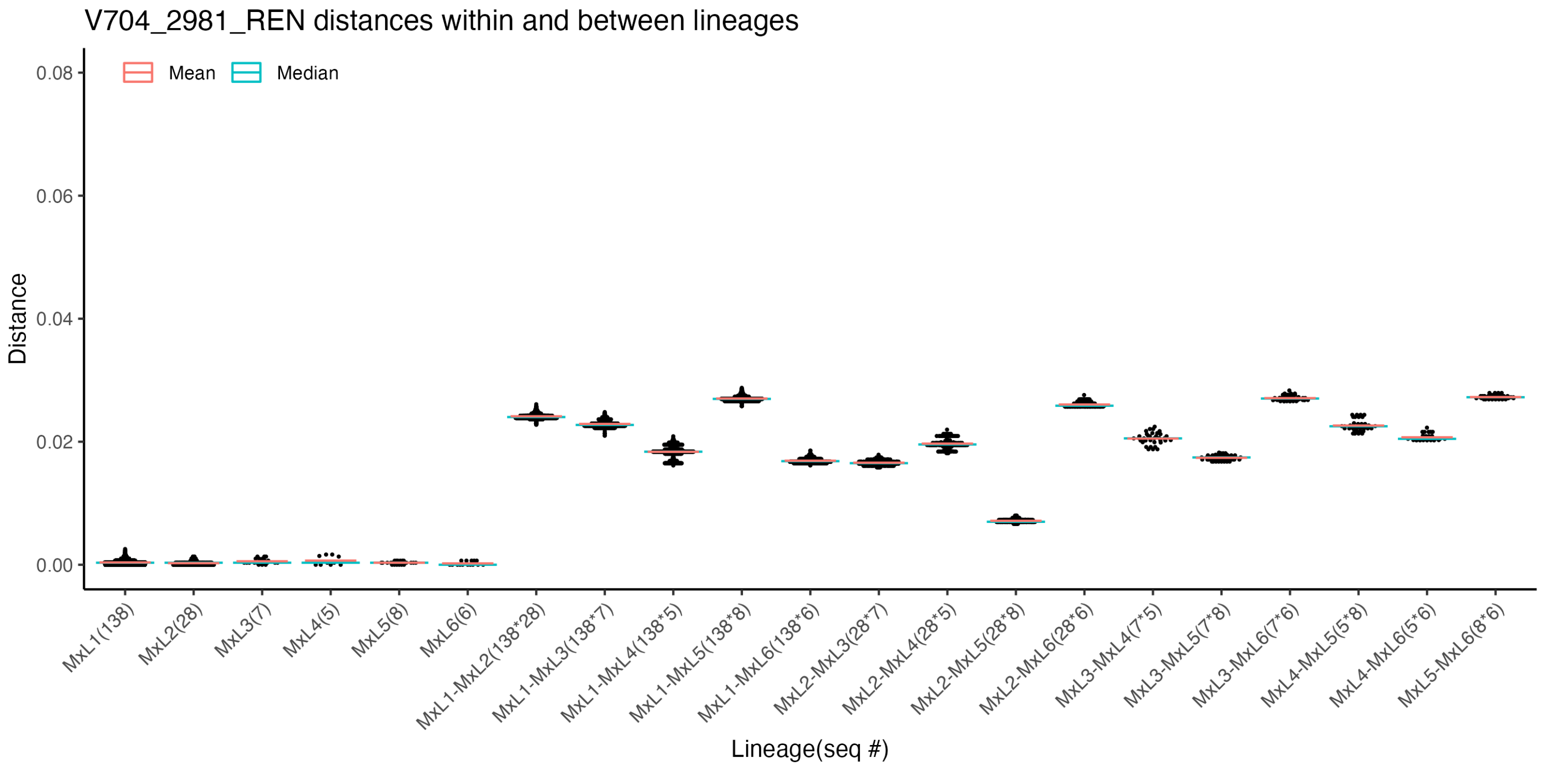

V704_2981_REN distances within and between lineages
Mean
Median
Distance
0.08
0.06
0.04
0.02
0.00
MxL1(138)
MxL2(28)
MxL3(7)
MxL4(5)
MxL5(8)
MxL6(6)
MxL1-MxL2(138*28)
MxL1-MxL3(138*7)
MxL1-MxL4(138*5)
MxL1-MxL5(138*8)
MxL1-MxL6(138*6)
MxL2-MxL3(28*7)
MxL2-MxL4(28*5)
MxL2-MxL5(28*8)
MxL2-MxL6(28*6)
MxL3-MxL4(7*5)
MxL3-MxL5(7*8)
MxL3-MxL6(7*6)
MxL4-MxL5(5*8)
MxL4-MxL6(5*6)
MxL5-MxL6(8*6)
Lineage(seq #)

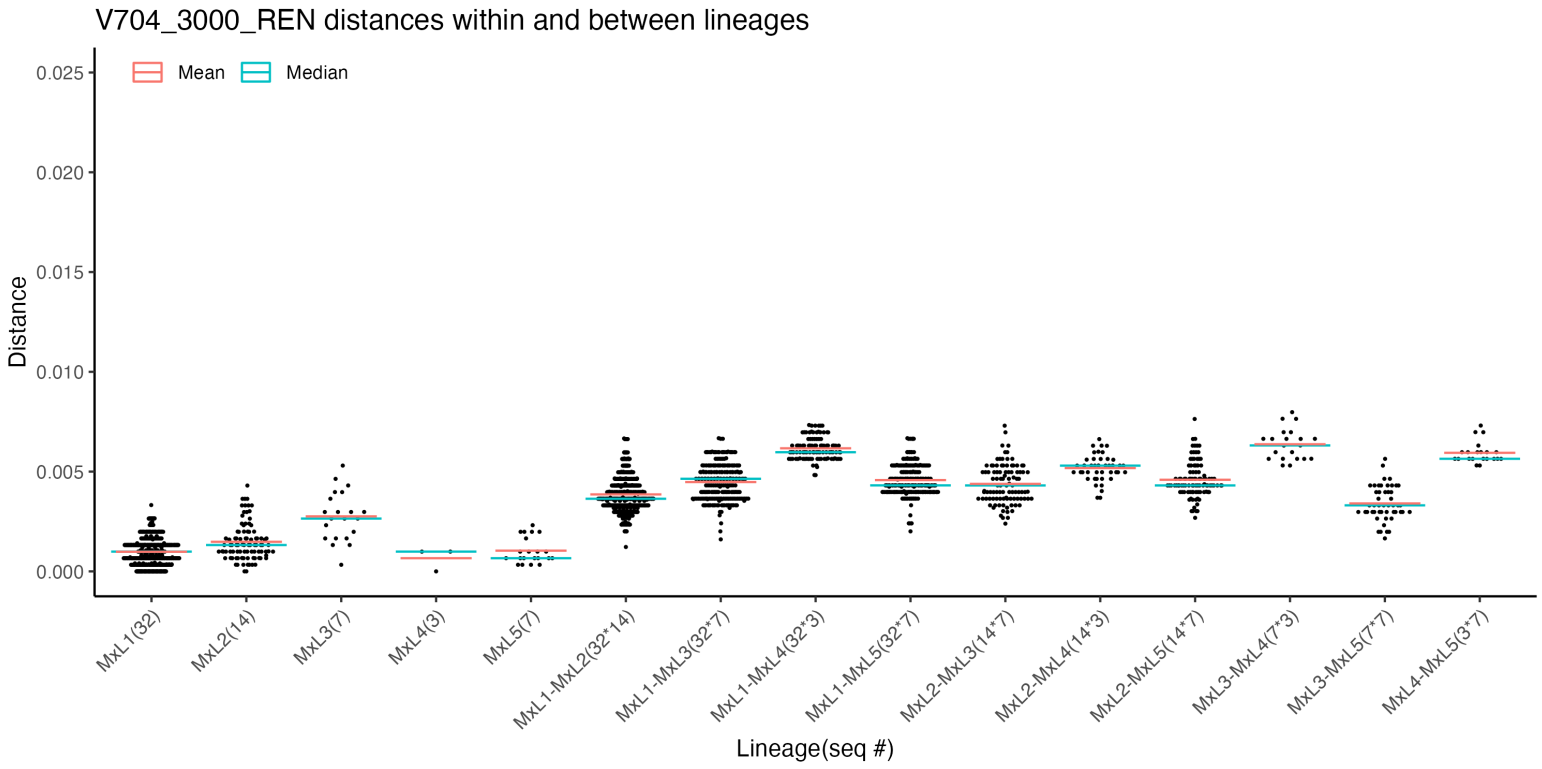

V704_3000_REN distances within and between lineages
Mean
Median
Distance
0.025
0.020
0.015
0.010
0.005
0.000
MxL1(32)
MxL2(14)
MxL3(7)
MxL4(3)
MxL5(7)
MxL1-MxL2(32*14)
MxL1-MxL3(32*7)
MxL1-MxL4(32*3)
MxL1-MxL5(32*7)
MxL2-MxL3(14*7)
MxL2-MxL4(14*3)
MxL2-MxL5(14*7)
MxL3-MxL4(7*3)
MxL3-MxL5(7*7)
MxL4-MxL5(3*7)
Lineage(seq #)

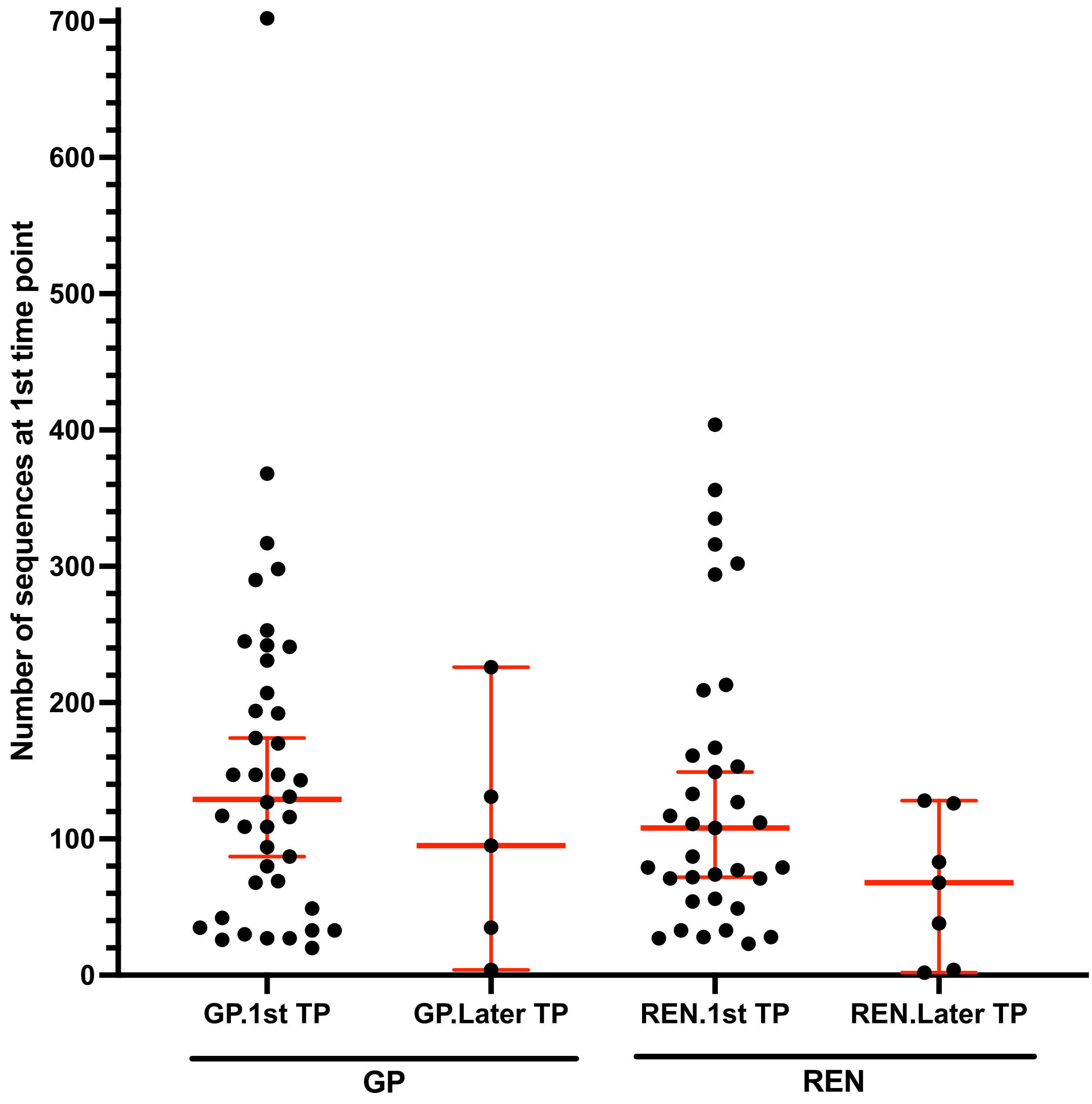

Supplementary Fig. 7. Number of sequence obtained at the first time point as a function of when multiple lineages were detected. "XX.1st TP" refers to detection of multiple lineages at the 1st time point examined. "XX.Later TP" refers to detection of multiple lineages only at later time point(s) examined.

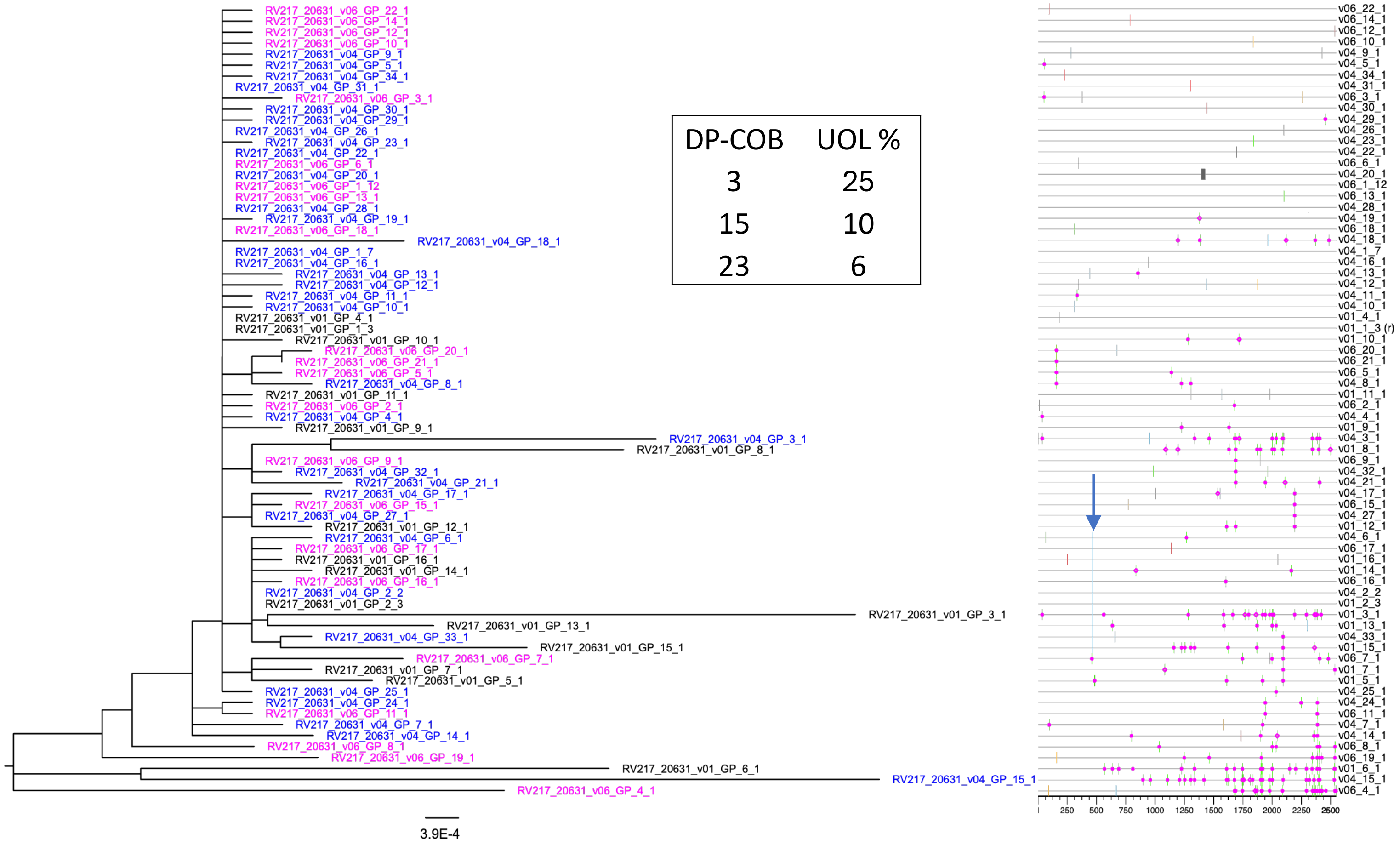

Supplementaty Fig. 8. Uncertain-Origin-Lineage (UOL). Phylobook output showing a phylogenetic tree and highlighter plot from a RV217 cohort participant. UOL sequences are shown as a blue stripe in the highlighter plot (blue arrow). The days post COB and the relative representation of the UOL at each time point are shown in the inset.

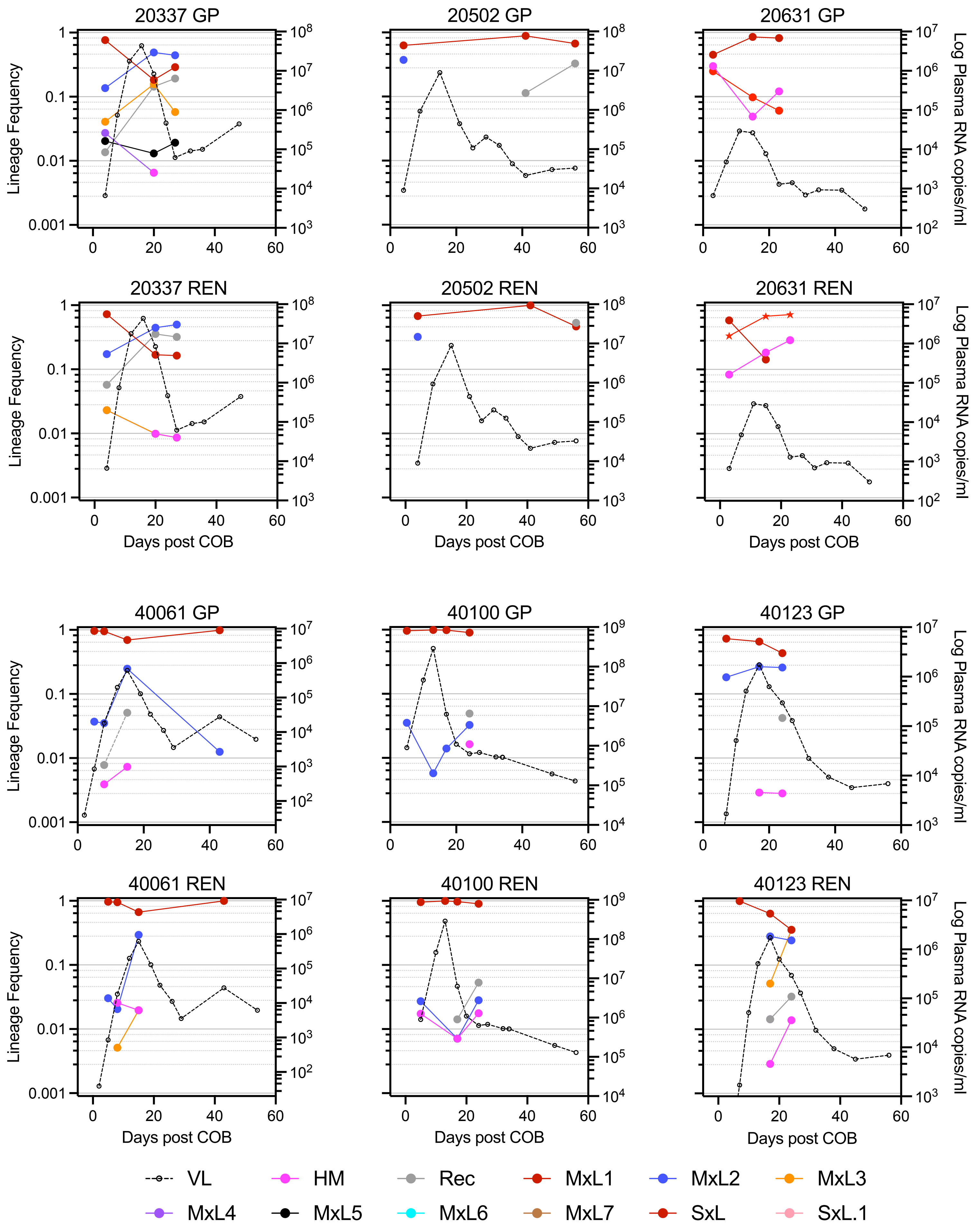

Supplementary Fig. 9.1. Plasma HIV RNA viral load and lineage frequencies over time. Data from participants from the RV217 cohort and with multiple transmitted lineages are shown. See legend to Fig 2 for key.

Supplementary Fig. 9.2.

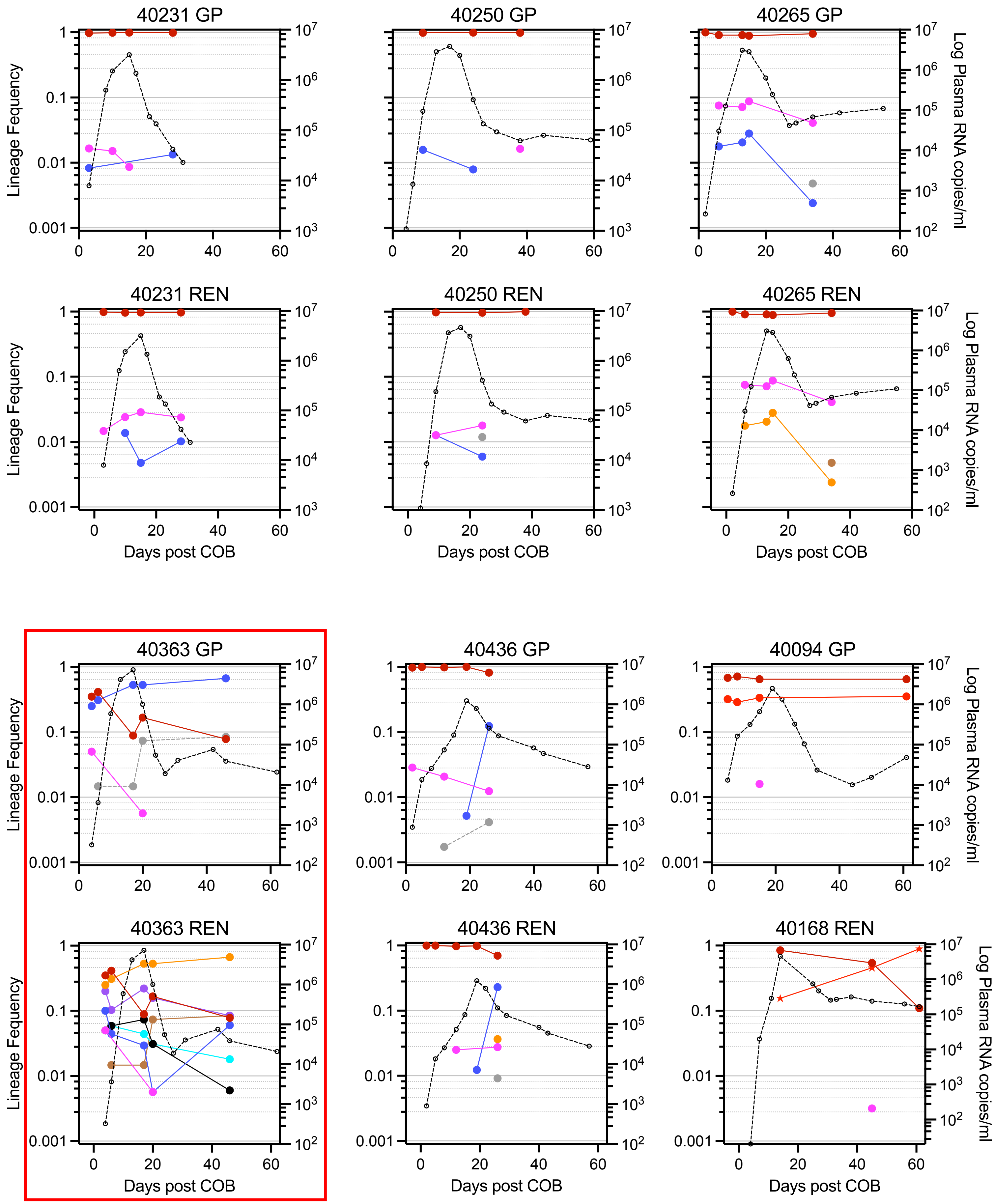

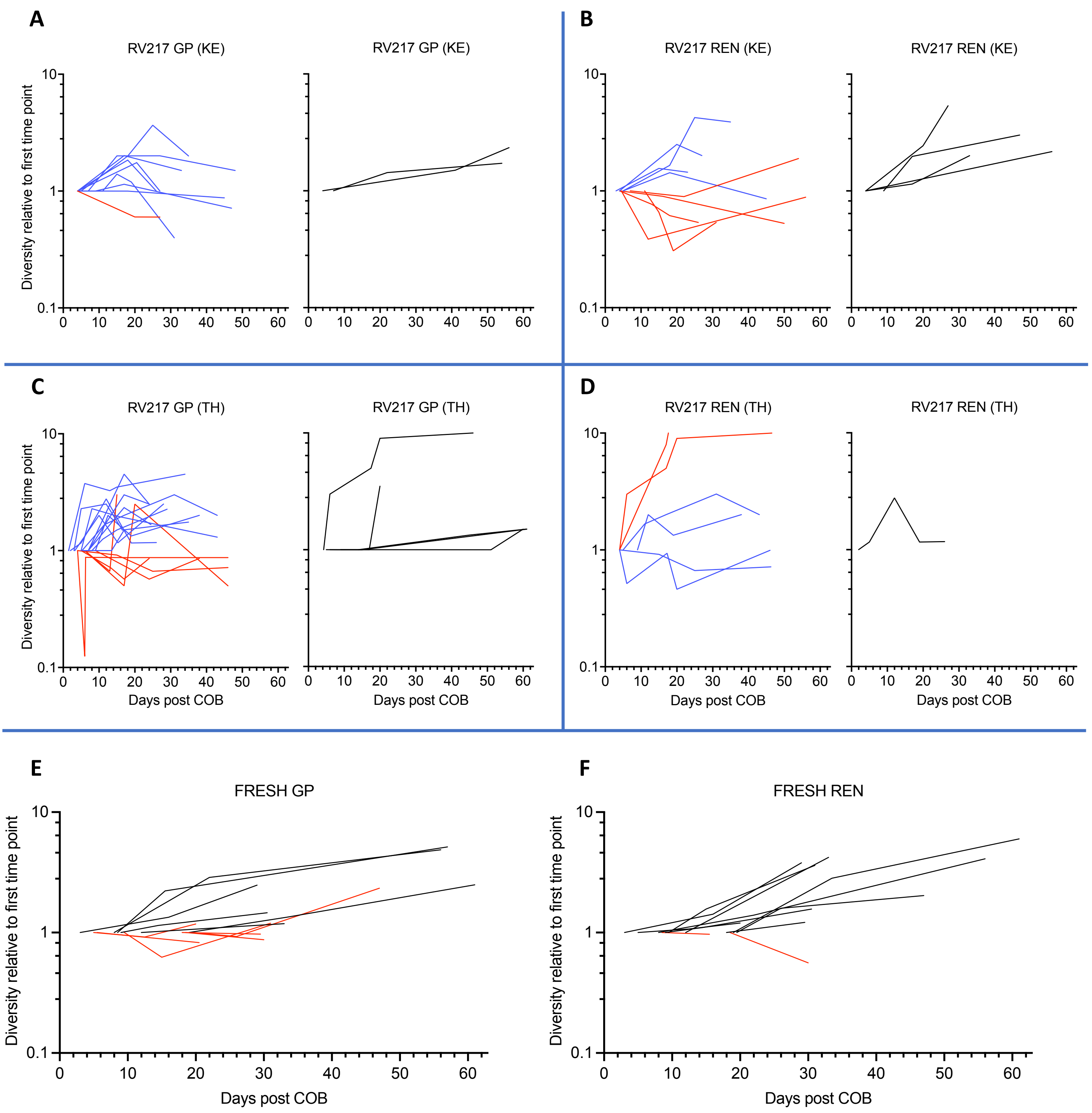

Supplementary Fig. 10 Viral population diversification. Maximum likelihood pairwise distances within all lineages from RV217 (**A-D**) and FRESH (**E-F**) cohorts are plotted as a function of days post COB for the GP (**A, C, E**) and REN (**B, D, F**) regions. All diversity measurements are plotted in relation to the diversity at the first time point (i.e., “1” on the y-axis). Red lines indicate instances in which the diversity decreased from the 1st to the 2nd time point, blue lines indicate instance in which the diversity decreased at any later time point, and black lines (plotted separately in A-D) indicate instances in which no drop in diversity was detected.

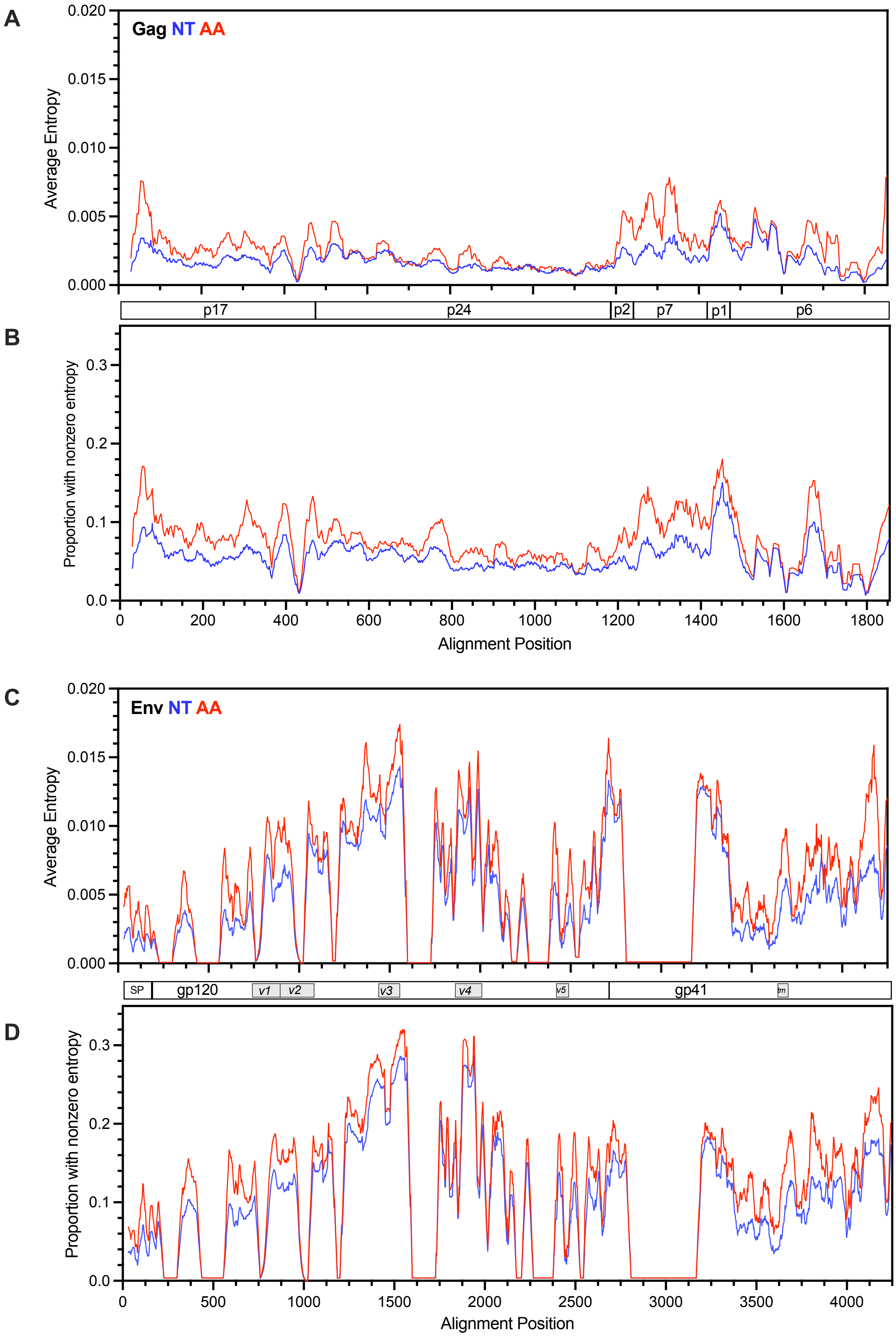

Supplementary Fig. 11. Entropy in the Gag and Env genes. Nucleotide (NT, blue lines) and amino acid (AA, red lines) alignments of sequences from all lineage were compared, in 30 NT or 10 AA sliding windows. Panels A and C show Shannon entropies, whereas panels B and D show the proportion of lineages that had nonzero entropy. Data was plotted using a full alignment of all lineages having at least 10 members derived from all participants.

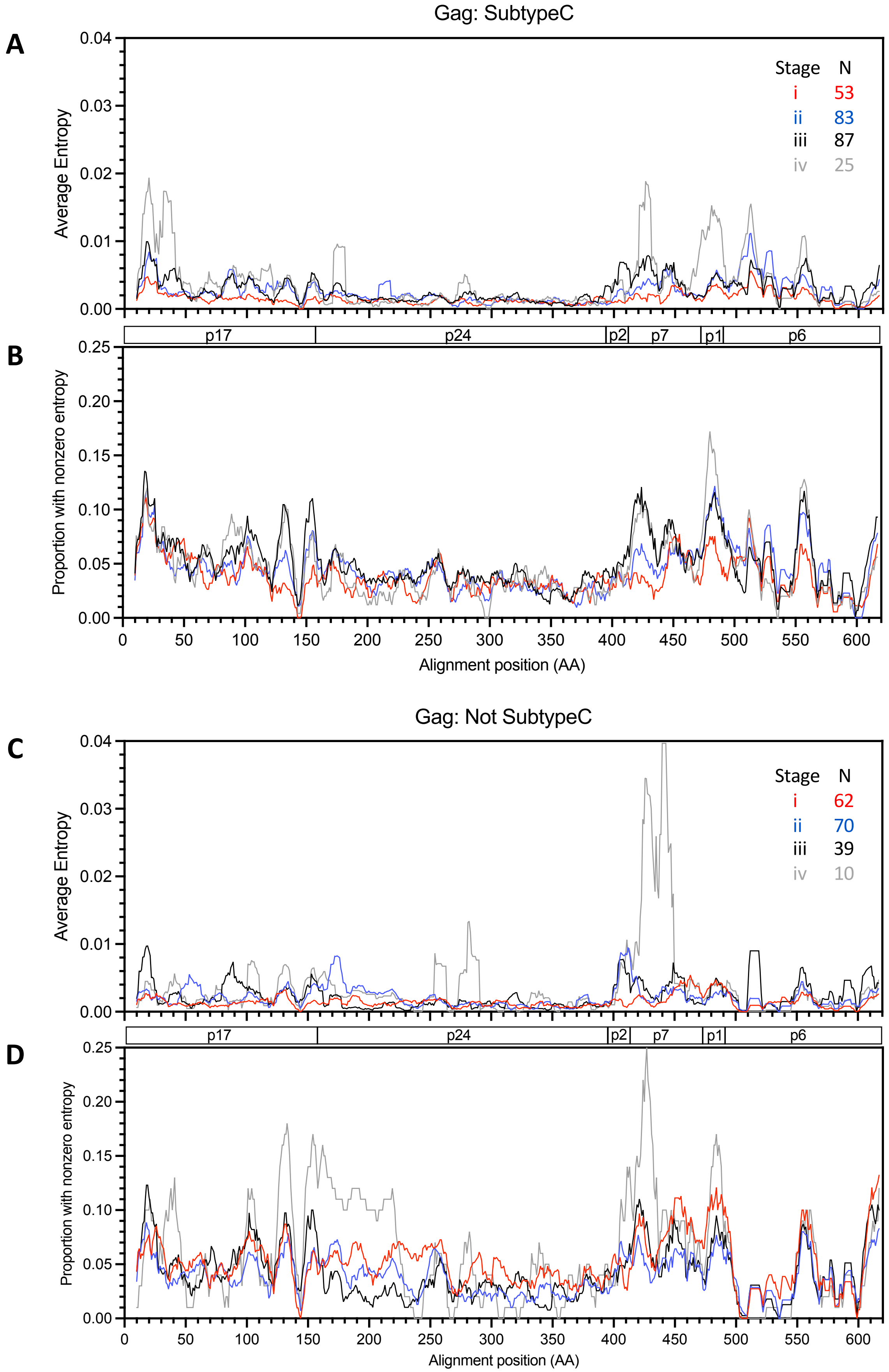

Supplementary Fig. 12. Entropy of Gag proteins during the i-iv stages of early infection. To limit variation due to small sample sizes, lineage-based datasets were split into Subtype C (**A, B**) and not Subtype C (**C, D**). Panels A and C show Shannon entropy values and B and D show the proportion of lineages with any entropy at a given amino acid position. Data is plotted in sliding windows of 10 amino acids using a full alignment of all lineages having at least 10 members derived from all participants. The keys in panels A and C show the color coding and the number of lineage datasets used to plot each stage pattern.

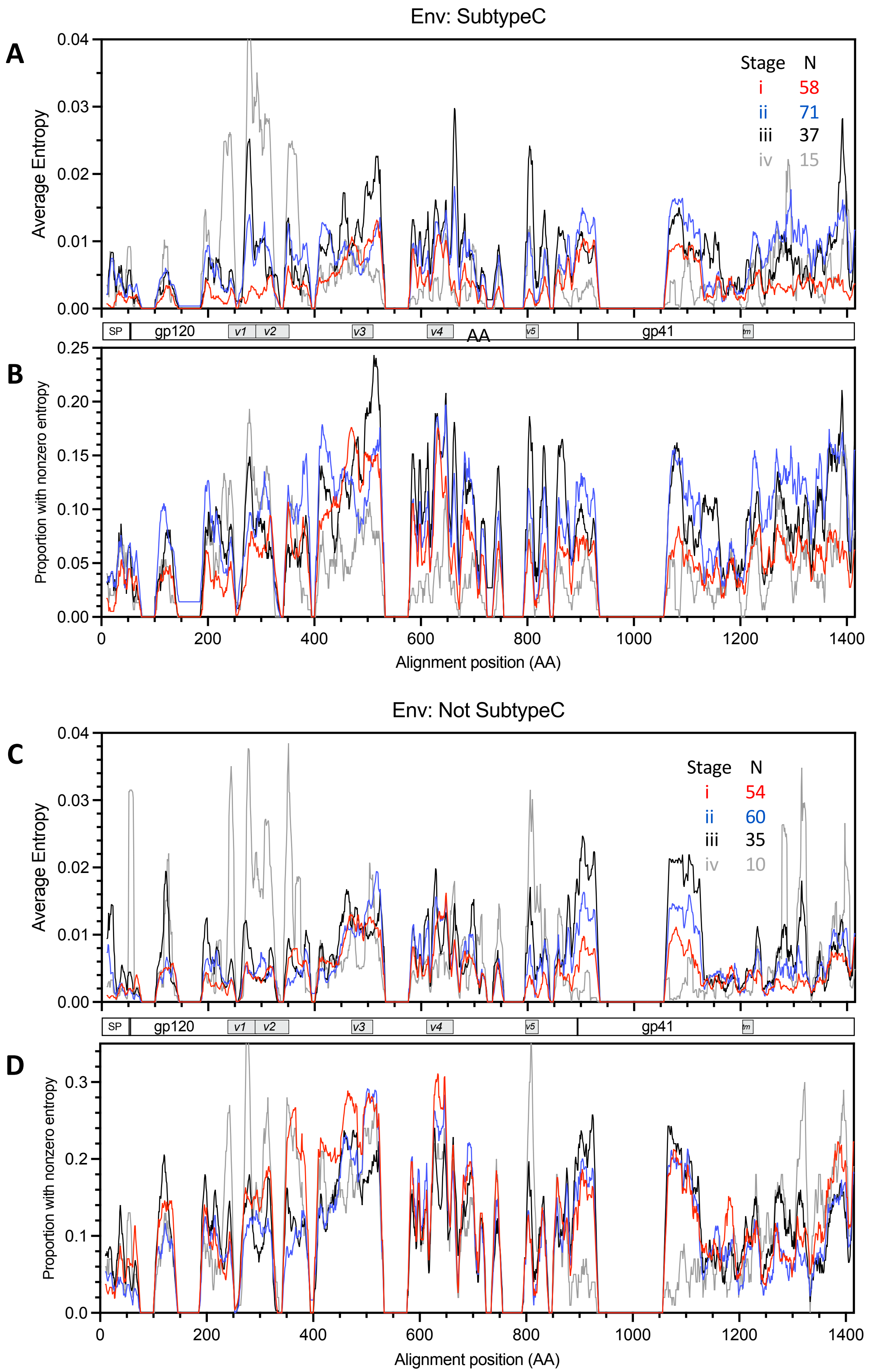

Supplementary Figure 13. Entropy of Env proteins during the i-iv stages of early infection. To limit variation due to small sample sizes, lineage-based datasets were split into Subtype C (**A, B**) and not Subtype C (**C, D**). Panels A and C show Shannon entropy values and B and D show the proportion of lineages with any entropy at a given amino acid position. Data is plotted in sliding windows of 10 amino acids using a full alignment of all lineages having at least 10 members derived from all participants. The keys in panels A and C show the color coding and the number of lineage datasets used to plot each stage pattern.

Supplementary Fig. 14. Number of potential N-linked glycosylation site by stage and subtype groupings. The red lines in panel **A** indicate medians and 95% CI.

A

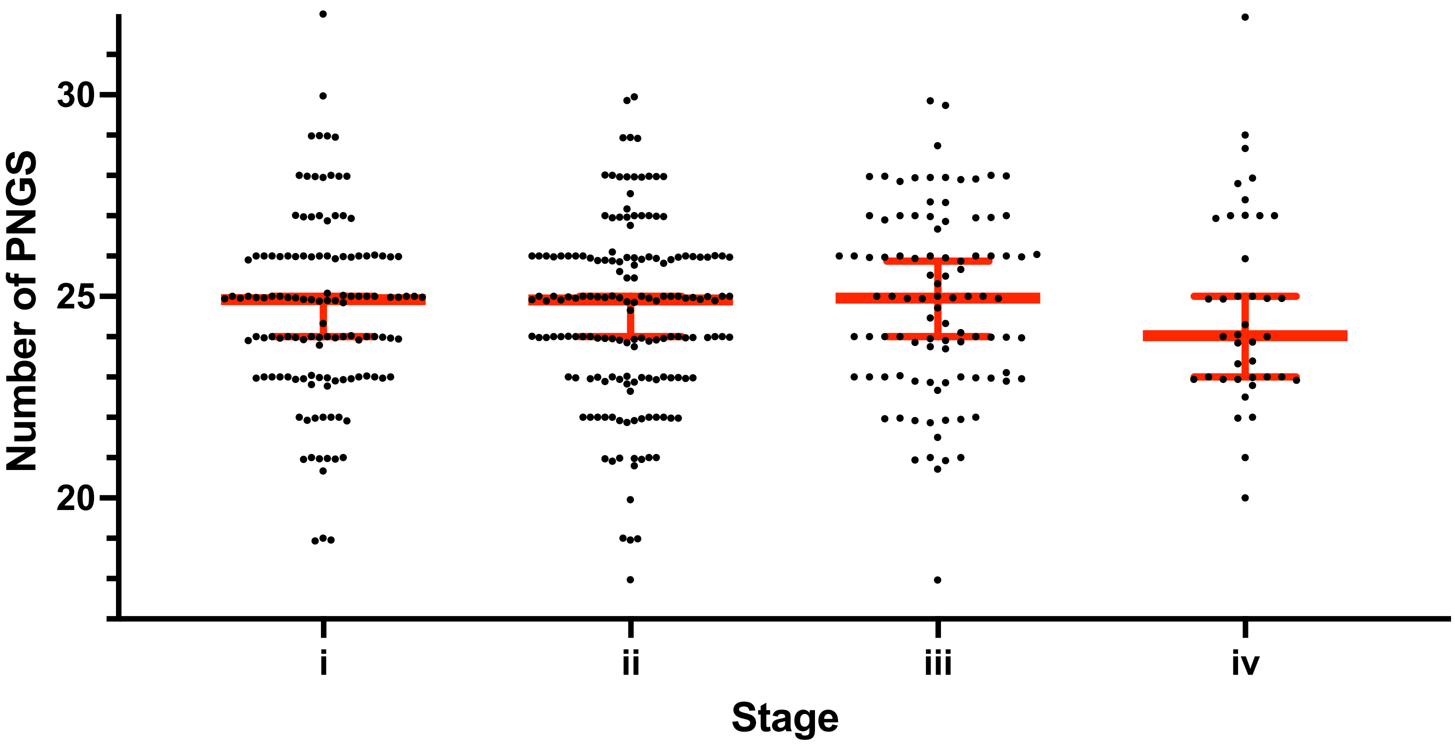

B

Numbers of PNGS by stage of acute infection and Subtype				
**Stage**	**i**	**ii**	**iii**	**iv**
**All cohorts**				
Mean	24.6	24.6	24.8	24.7
Median	24.9	24.9	25.0	24.0
**Subtype C**				
Mean	24.2	24.2	24.3	24.6
Median	24.0	24.0	24.0	24.2
**NonC**				
Mean	25.1	25.0	25.5	24.9
Median	25.0	25.0	25.3	24.0

Supplementary Fig. 15. Selected sites within and immediately surrounding PNGS. The number of AA sites within and nearby NLGS sequons that experienced positive and negative selection above a 0.9 probability threshold are shown. Positively (**A**) and negatively (**B**) selected sites that impact sequons and adjacent sequences are indicated. Selected sites N-terminal (negative numbers on the x-axis) and C-terminal (positive numbers) to the sequons are shown with blue bars. Loss (panel A) or preservation (panel B) of PNGS are shown in shades of red with the positions within the NX(T/S) sequon. The cyan bar in the N column of panel A indicates a single gain of a PNGS, and the black bars in the X positions indicate the selection of a Proline at this position, which greatly diminishes the likelihood of glycosylation.

A

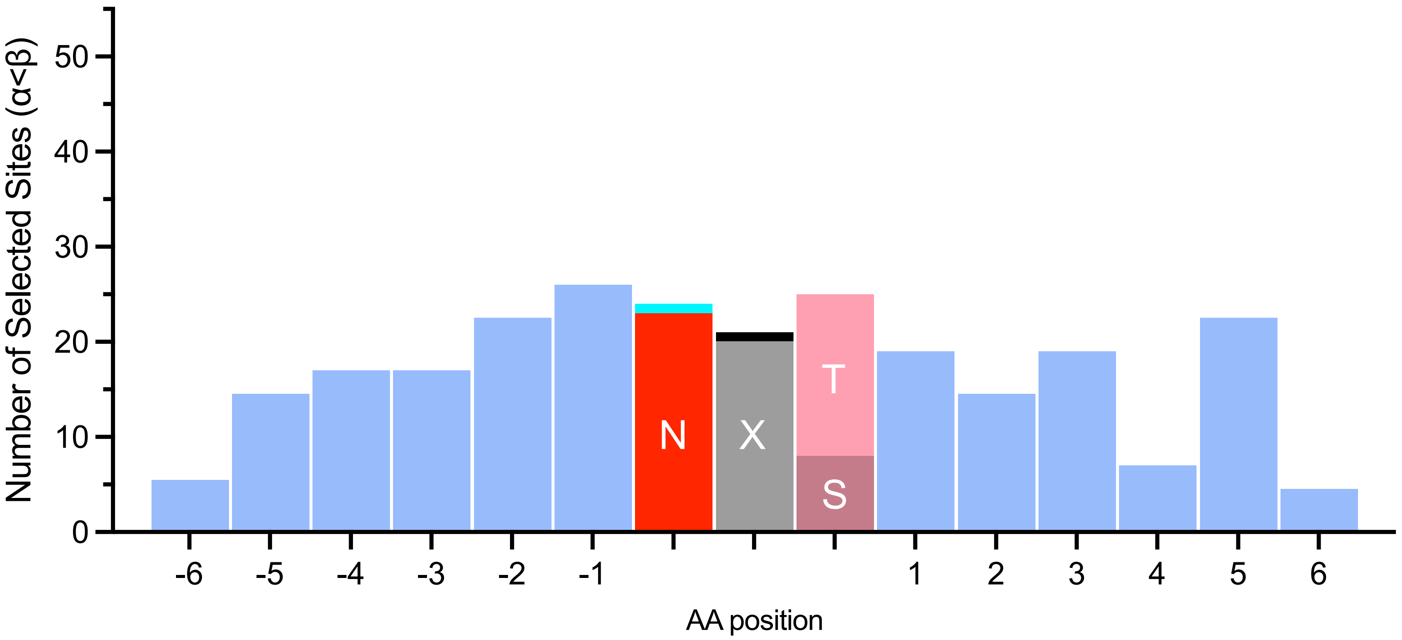

B

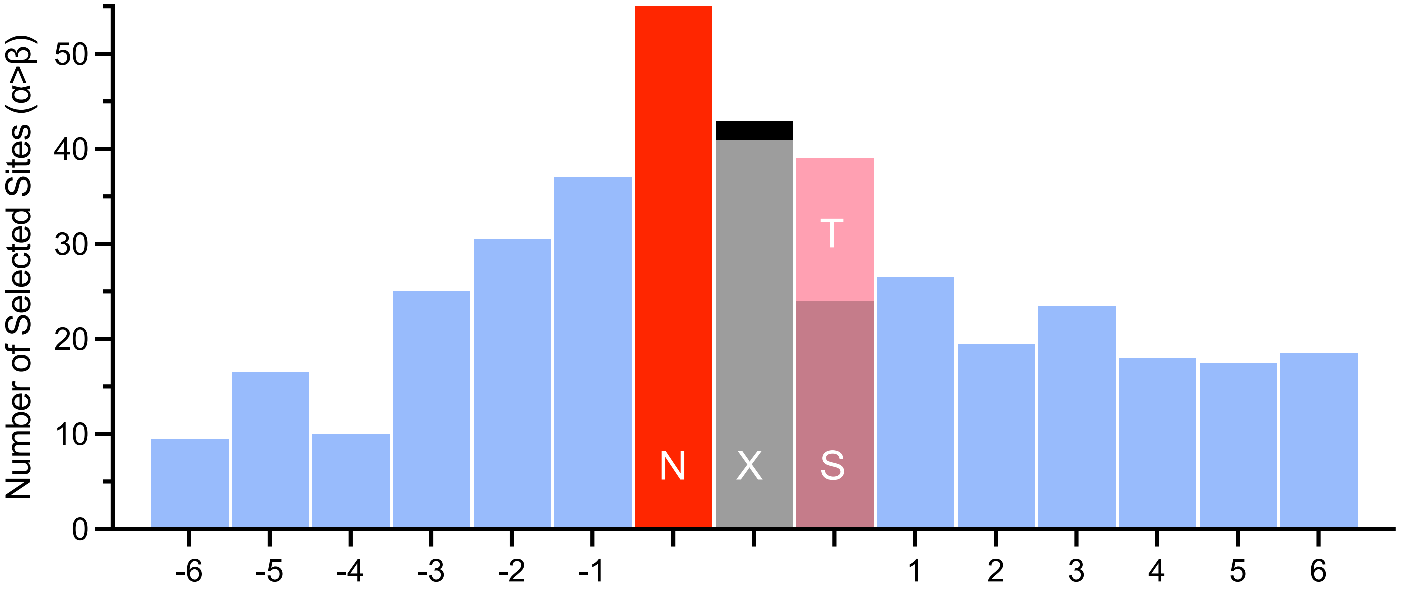

A

B

C

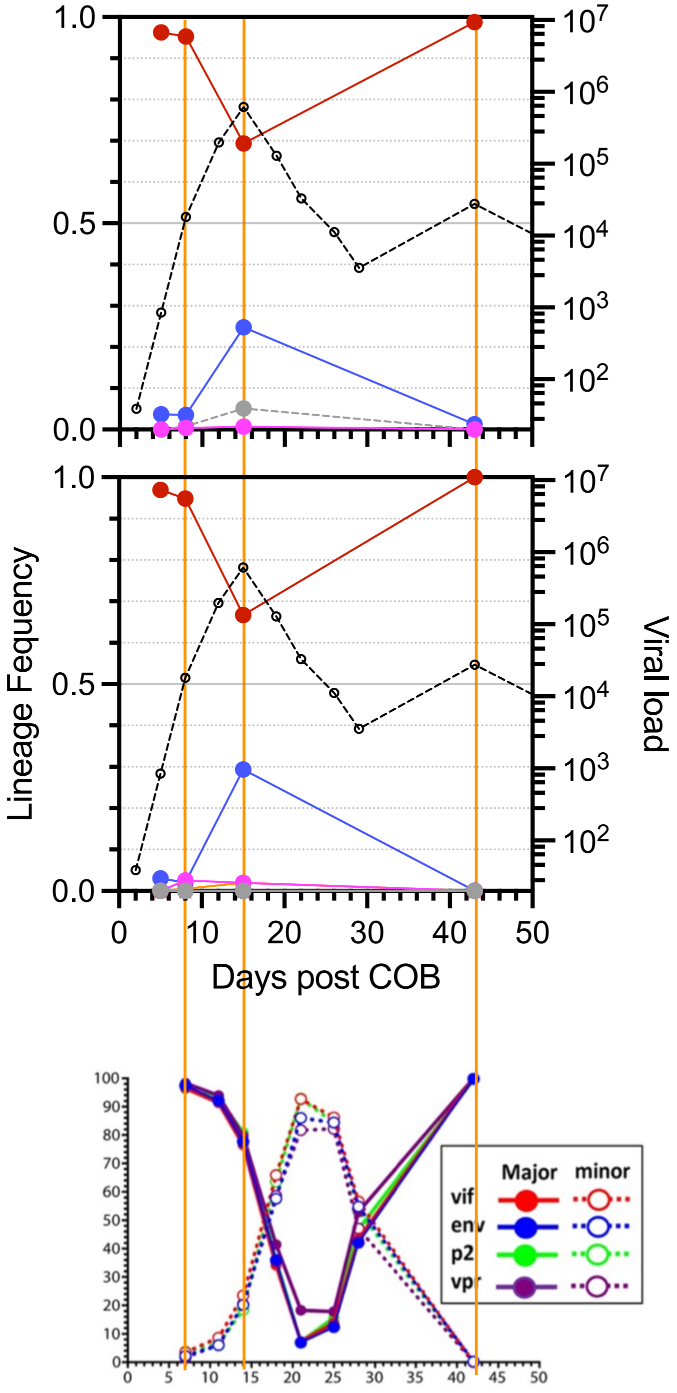

Supplementary Fig. 16. Comparison of lineage frequencies over time from the current and a previous study. Data from participant 40061 from the RV217 cohort and with multiple transmitted lineages is shown. Lineage frequencies in GP (**A**) and REN (**B**) from this study as well as major and minor lineages detected in 4 viral genome regions from a previous study by Kijak et al (PLoS pathogens. 2017;13(7):e1006510) (**C**). The major and minor lineages described by Kijak et al correspond to the 1st (red symbols) and 2nd (blue) most prevalent lineages from the current study. As shown by the intervening time points sequenced by Kijak et al, the minor lineage was transiently the most abundant in the interval between 14-42 days COB.
