# Supplementary material for: Long-read deep sequencing reveals high rates of multilineage transmission and rapid viral population changes in acute HIV infection
Source: Nat Commun. 2026 May 25;17:6799. doi: 10.1038/s41467-026-73496-0 (PMC13385744; doi:10.1038/s41467-026-73496-0)
Supplement: Supplementary file 3 — Reporting Summary [file 41467_2026_73496_MOESM3_ESM.pdf]

Corresponding author(s): James I Mullins

Last updated by author(s): 8 Mar 2026

Reporting Summary

Nature Portfolio wishes to improve the reproducibility of the work that we publish. This form provides structure for consistency and transparency in reporting. For further information on Nature Portfolio policies, see our Editorial Policies and the Editorial Policy Checklist.

Statistics

For all statistical analyses, confirm that the following items are present in the figure legend, table legend, main text, or Methods section.

n/a	Confirmed	
☐	☒	The exact sample size (*n*) for each experimental group/condition, given as a discrete number and unit of measurement
☒	☐	A statement on whether measurements were taken from distinct samples or whether the same sample was measured repeatedly
☐	☒	The statistical test(s) used AND whether they are one- or two-sided<br>*Only common tests should be described solely by name; describe more complex techniques in the Methods section.*
☐	☒	A description of all covariates tested
☒	☐	A description of any assumptions or corrections, such as tests of normality and adjustment for multiple comparisons
☐	☒	A full description of the statistical parameters including central tendency (e.g. means) or other basic estimates (e.g. regression coefficient) AND variation (e.g. standard deviation) or associated estimates of uncertainty (e.g. confidence intervals)
☐	☒	For null hypothesis testing, the test statistic (e.g. *F*, *t*, *r*) with confidence intervals, effect sizes, degrees of freedom and *P* value noted<br>*Give P values as exact values whenever suitable.*
☒	☐	For Bayesian analysis, information on the choice of priors and Markov chain Monte Carlo settings
☒	☐	For hierarchical and complex designs, identification of the appropriate level for tests and full reporting of outcomes
☒	☐	Estimates of effect sizes (e.g. Cohen's *d*, Pearson's *r*), indicating how they were calculated

Our web collection on statistics for biologists contains articles on many of the points above.

Software and code

Policy information about availability of computer code

Data collection	Software used for DNA sequence processing was described in Westfall et al (2024) and freely available at github.com/MurrellGroup and github.com/MullinsLab/mullins-site.
Data analysis	Location of some software is described in Methods section, additional software is provided at https://github.com/MullinsLab/NatComm_scripts.

For manuscripts utilizing custom algorithms or software that are central to the research but not yet described in published literature, software must be made available to editors and reviewers. We strongly encourage code deposition in a community repository (e.g. GitHub). See the Nature Portfolio guidelines for submitting code & software for further information.

Data

Policy information about availability of data

All manuscripts must include a data availability statement. This statement should provide the following information, where applicable:

- Accession codes, unique identifiers, or web links for publicly available datasets
- A description of any restrictions on data availability
- For clinical datasets or third party data, please ensure that the statement adheres to our policy

Genbank accession numbers for all sequences are provided in the manuscript

Research involving human participants, their data, or biological material

Policy information about studies with human participants or human data. See also policy information about sex, gender (identity/presentation), and sexual orientation and race, ethnicity and racism.

Reporting on sex and gender	The cohorts evaluated were existing and involved either adult women, men or transgender women. This information for each cohort was defined in the text
Reporting on race, ethnicity, or other socially relevant groupings	N/A
Population characteristics	All participants were had acquired HIV infection.
Recruitment	All participants in our study were drawn from the preexisting cohorts described in the manuscript.
Ethics oversight	The Institutional Review Boards/Ethic Committees of participating clinical research sites (CRS) approved the studies, which were conducted under the oversight of the NIAID Data Safety Monitoring Board. Viral genome sequencing at the University of Cape Town was approved by the UCT Human Research Ethics Committee (HREC reference no. 176/2017) and was considered exempt at the University of Washington.

Note that full information on the approval of the study protocol must also be provided in the manuscript.

Field-specific reporting

Please select the one below that is the best fit for your research. If you are not sure, read the appropriate sections before making your selection.

- [x] Life sciences
- [ ] Behavioural & social sciences
- [ ] Ecological, evolutionary & environmental sciences

For a reference copy of the document with all sections, see nature.com/documents/nr-reporting-summary-flat.pdf

Life sciences study design

All studies must disclose on these points even when the disclosure is negative.

Sample size	Sample size for the AMP trial cohorts was determined based on the expectations of infection incidences sufficient to show a difference between placebo and product recipients. (See Corey et al). All placebo recipients were included in this study, whereas no product recipients were included. FRESH and RV217 trial recipients were chosen for a study to generate a method for estimating the date of detectable infection for individuals sampled less frequently (See Rossenkhan et al).
Data exclusions	Whereas all sequence data was included for most studies, for lineage-specific analyses, data from lineages with fewer than 10 members were excluded in order to dampen outlier effects.
Replication	Replication was not specifically addressed
Randomization	Not applicable
Blinding	Investigators were blinded to experimental group in the AMP trial prior to completion of sequencing and analyses related to AMP trial efficacy.

Reporting for specific materials, systems and methods

We require information from authors about some types of materials, experimental systems and methods used in many studies. Here, indicate whether each material, system or method listed is relevant to your study. If you are not sure if a list item applies to your research, read the appropriate section before selecting a response.

Materials & experimental systems

n/a	Involved in the study	
x		Antibodies
x		Eukaryotic cell lines
x		Palaeontology and archaeology
x		Animals and other organisms
	x	Clinical data
x		Dual use research of concern
x		Plants

Methods

n/a	Involved in the study	
x		ChIP-seq
x		Flow cytometry
x		MRI-based neuroimaging

Clinical data

Policy information about clinical studies

All manuscripts should comply with the ICMJE guidelines for publication of clinical research and a completed CONSORT checklist must be included with all submissions.

Clinical trial registration	Not applicable
Study protocol	Not applicable
Data collection	Not applicable
Outcomes	Not applicable

Plants

Seed stocks	Not applicable
Novel plant genotypes	Not applicable
Authentication	Not applicable
