# Supplementary material for: Long-read deep sequencing reveals high rates of multilineage transmission and rapid viral population changes in acute HIV infection
Source: Nat Commun. 2026 May 25;17:6799. doi: 10.1038/s41467-026-73496-0 (PMC13385744; doi:10.1038/s41467-026-73496-0)
Supplement: Supplementary file 2 — Peer Review file [file 41467_2026_73496_MOESM2_ESM.pdf]

nature portfolio

Peer Review File

Long-Read Deep Sequencing Reveals High Rates of Multilineage Transmission and Rapid Viral Population Changes in Acute HIV Infection

Corresponding Author: Professor James Mullins

This file contains all reviewer reports in order by version, followed by all author rebuttals in order by version.

Version 0:

Reviewer comments:

Reviewer #1

(Remarks to the Author)
Comments
The authors employ long-read PacBio single-molecule real-time (SMRT) sequencing with UMI correction to characterize early HIV populations across four intensively sampled cohorts. The use of SMRT-UMI substantially reduces technical artifacts associated with PCR amplification and sequencing errors, enables reconstruction of long-range haplotypes, and provides compelling evidence that early HIV populations are more complex and dynamic than previously appreciated. The study further suggests that multiple lineage acquisitions (MLA) frequently arise from the transmission of multiple viral variants from donors rather than from de novo diversification in recipients, leading to the conclusion that the number of transmitted viruses may be higher than previously estimated.

By combining advanced sequencing technologies with dense longitudinal sampling across multiple cohorts, this work significantly extends our understanding of early intra-host HIV diversity and lineage dynamics. However, despite these strengths, I have two major concerns regarding the interpretation of the results.

Major Concern 1 — Distinguishing transmitted lineages from early de novo diversification in the absence of donor–recipient pairs
The combined application of Poisson Fitter and phylogenetic/distance-based analyses is a major strength of this study and represents one of the most rigorous efforts to characterize early HIV population structure to date. However, despite these methodological advances, the available data do not permit a definitive distinction between viral lineages that were transmitted at exposure and those that emerged through extremely early de novo diversification following transmission. This limitation is particularly relevant for the uncertain-origin lineages (UOLs), which differ from dominant lineages by only 1–3 substitutions.

At a more fundamental level, the lack of donor–recipient transmission pairs precludes direct assessment of whether the detected lineages were present in the donor prior to transmission. As a result, inferences regarding the frequency of multiple transmitted lineages rely exclusively on indirect evidence derived from recipient-only data. The authors are therefore encouraged to more clearly distinguish conclusions about early within-host population complexity from those about the intrinsic stringency of the HIV transmission bottleneck.

The authors' interpretation appears to rely, at least implicitly, on the relatively low mutation rate of HIV (~2–5 × 10^{-5} mutations per site per replication cycle, corresponding to ~0.2–0.5 substitutions per genome per cycle), which would argue against extensive early de novo diversification. While this argument is generally reasonable, it warrants more careful consideration for several reasons. First, the estimated center of bounds (COB) corresponds to approximately seven days post-acquisition, implying that multiple viral replication cycles may have occurred before viral RNA became detectable in plasma. During this interval, each viral genome could accumulate multiple de novo mutations. Second, a substantial proportion of secondary lineages in MLA cases are present at relatively low frequencies (often in the ~0.1%–10% range). Given known intra-host substitution rates and the potential for strong early selective pressures, it is biologically plausible that variants differing by only a small number of substitutions could arise rapidly through early de novo diversification following transmission. This raises the possibility that some low-frequency lineages—particularly those detected at only a single time

point or in a single genomic region—may not necessarily represent independently transmitted lineages.

Overall, donor–recipient transmission pairs remain the most direct and compelling evidence for distinguishing transmitted lineages from early intra-host diversification. In the absence of such data, the authors should apply particularly careful and conservative criteria when interpreting low-frequency or minimally divergent lineages as evidence of multiple transmitted viruses.

Major Concern 2 — Evolutionary forces underlying intra-host diversity and lineage turnover
The manuscript uses the FUBAR framework to infer selection acting on early HIV populations and concludes that negative selection predominates. While this observation is informative, it appears insufficient to explain the pronounced lineage fluctuations, frequent lineage turnover, and replacement of dominant lineages observed in many individuals. In multiple cases, lineages rise and fall over time rather than showing consistent directional changes, suggesting that simple unidirectional purifying selection does not fully capture the underlying evolutionary dynamics.

A key conceptual framework that is not explicitly considered is pleiotropy and fitness trade-offs across biological scales, particularly between viral fitness within a host and fitness for transmission between hosts. Extensive prior work has demonstrated that HIV variants evolving late in infection often acquire phenotypes that enhance survival and replication within the host—such as altered Env glycosylation, expanded coreceptor usage, and the ability to infect a broader range of target cells including macrophages and naïve T cells—but that these same features are disfavored during transmission. In contrast, transmitted/founder viruses are typically characterized by CCR5 usage, high CD4 dependence, limited glycosylation, and a preference for mature $CD4^+$ T cells, features that appear repeatedly selected at transmission despite extensive diversification in the donor (PMC3700278; PMC3911544). Similar patterns have been reported in SARS-CoV-2, where mutations may confer little or no fitness advantage within hosts but enhance transmissibility between hosts (e.g., PMC10521905; PMC11021471).

This body of evidence supports a model in which mutations that are advantageous—or at least tolerated—within hosts may be neutral or deleterious for transmission, and vice versa. Such antagonistic pleiotropy provides a plausible explanation for several observations in the present study: (i) the persistence of substantial intra-host diversity early after infection, (ii) large temporal fluctuations in lineage frequencies, and (iii) the frequent replacement of initially dominant lineages by variants that were previously minor. Under this model, lineage turnover does not necessarily reflect simple positive or negative selection, but rather shifting fitness landscapes shaped by cell-type availability, immune pressure, and changing ecological niches within the host. Incorporating such perspectives would strengthen the evolutionary interpretation of the observed lineage dynamics.

Overall, this is a technically impressive and conceptually important study that leverages advanced long-read sequencing, dense sampling, and multiple cohorts to provide new insight into early HIV population structure. The data demonstrate that early HIV populations are more diverse and dynamic than previously appreciated. Addressing the concerns raised above—particularly those related to early de novo diversification, evolutionary interpretation—would substantially strengthen the robustness, clarity, and interpretability of the conclusions.

Minor Comment — Definition of multilineage acquisition (MLA)
A more explicit operational definition of multilineage acquisition (MLA) is needed. In the main text, the authors state that the frequency of MLA was determined by considering both the GP and REN regions across all sampled time points; however, the precise criteria used to define a distinct lineage are not clearly specified. While uncertain-origin lineages (UOLs) are defined as differing from a dominant lineage by 1–3 substitutions, it remains unclear whether MLA designation required (i) a minimum number of consistent nucleotide differences across sequences, and/or (ii) a minimum relative frequency threshold at any given time point.

Clarification of these criteria would improve reproducibility, reduce ambiguity in lineage classification, and facilitate comparison with prior studies investigating HIV transmission bottlenecks and transmitted/founder virus multiplicity.

Other Minor comments/suggestions, noted by Page/Line.
1. P5/L161: “Poisson Fitter21 (Supplementary Discussion 4)” is cited however, only Supplementary Discussions 1-3 are present in the Supplementary Information. Please verify and correct this reference.

2. P6/L198: “(see FRESH participants 079, 271 and 318 in Fig 2, and RV217 participants 20337, 20502 and 40363 in and 318 in Fig. 2, and RV217 participants 20337, 20502, 40061, 40265 and 40436 in Supplementary Fig. 9)”. The citation of participant identifiers and figure references contains apparent duplication and inconsistencies. Please carefully revise this sentence to ensure that each participant is correctly linked to the appropriate figure.

3. P15/L529: “FUBAR algorithm modified to perform calculations in batch (https://github.com/MullinsLab/FUBAR_in_batch) .” The URL provided for the modified FUBAR algorithm is not accessible. Please verify the link or provide an alternative reference.

Reviewer #2

(Remarks to the Author)
Mullins et al. used single molecule long read sequencing to analyze gag-pol (GP) and rev-vpu-env-nef (REN) regions of the HIV genome from geographically disparate cohorts of PWH at 2-5 timepoints within 61 days of infection, thus aiming to study diversity and selective forces in acute HIV infection. The major reported finding of the study is a multilineage infection rate minimally estimated at 38%, which is higher than previous studies. Other findings include fluctuations of 10-fold or more in some individuals with multiple transmitted lineages, differences in rates of hypermutated and defective genomes between HIV clades, and the identification of conserved gene regions and regions evolving under negative or positive selection in gag and env.

The study addresses an important issue using cutting-edge technology and unique sets of samples from human participants. The findings are novel and interesting, and the arguments in the paper are clear. This reviewer particularly appreciated the manuscript's treatment of "transmitted/founder" sequence terminology and its concise treatment of vaccine development implications at the end of the Discussion section.

This reviewer's largest concerns about the manuscript relate to strength of findings and transparency of methods. Addressing the concerns described below will help readers critically evaluate the arguments in the manuscript.

1. The manuscript should clarify the strength of findings about multilineage infection.
a. While some scatter plots in Supp. Fig. 6 strongly support multilineage infection, there appear to be 19 cases (listed below) in which multilineage infection is supported by only one sequence for the given region in the individual. Also, it seems that only one of the two regions sequenced is shown for these cases. Does this mean that multilineage infection was not seen for the other region in these cases? The manuscript should explicitly address how it is possible to know that these lone sequences from putative secondary lineages were not due to UMI "carryover" (line 442) combined with error, to contamination, or to other artifacts. FSH_318_GP, V703_1313_GP, V703_1586_GP, V703_1689_GP, V703_1764_GP, V703_1848_GP, V703_1889_GP, V703_0790_REN, V703_0865_REN, V703_1313_REN, V703_1407_REN, V703_1789_REN, V703_3000_REN, V704_2073_GP, V704_0423_REN, V704_0652_REN, V704_1716_REN, V704_2073_REN, V704_2315_REN.
b. Line 430, "Westfall et al estimated PCR and sequencing error rates using this method paired with the PORPIDpipeline software to be less than 8.6 x 10-8 per base, or less than one error in every ~3700 432 REN sequences." This text suggests that almost all sequences generated in this study are 100% correct. What about errors in reverse-transcription of sample RNA to cDNA using commercially provided enzyme? Should these not occur at rates of 10-5 – 10-4 per base, for an average of one error every 3 – 30 REN sequences? The manuscript's treatment of error rates should be complete.
c. While the use of a manual inspection process to determine numbers of clades in the datasets from individual participants is certainly defensible, the manuscript could do more to illustrate how these decisions were made. Supplementary Fig. 5 is probably not accessible enough to educate readers who do not do this type of work themselves.

2. The manuscript presents data from RV217 and FRESH (total N=42 participants), with twice weekly sampling, combined with data from N=81 participants in placebo arms from the two AMP trials, with once monthly sampling. Does combining data from studies with very different sampling schedules cause any complications in analyses that relate to presumptive time since transmission? The manuscript refers to previous studies by Gilbert and Rossenkhan to describe how estimated dates of detectable infection (EDDI) were derived from HIV diagnostics and viral sequence data, but the manuscript should probably describe at least the logic of these previous analyses.

3. Line 156, "when sample datasets with no hypermutated sequences were excluded, there was a strong trend for hypermutated sequences to decline with increasing viral loads…." In Supp. Fig. 4, it appears that the number of samples with no detectable hypermutated sequences is large, so that the reported strong trend excludes quite a bit of data. The manuscript should address this. Can anything more be said about which samples do and do not include hypermutated sequences? Do samples without hypermutants preferentially come from specific participants? Is it possible that the sensitivity of the hypermut analysis is not uniform across participants and/or clades?

4. Lines 343-348 attribute the higher rate of defective gag and env genes in subtype C to lower RT fidelity, because the higher rate of defective genes was not associated with higher detected rates of APBOBEC-mediated hypermutation. However, is it not also possible that subtype C sequences use more codons that are 1 single-nucleotide mutation away from stop codons? Of particular interest would be codons where transitions could result in a premature stop (i.e., CAG, CGA, CAA, and TGG). The possibility of different codon usage resulting in higher rates of defective genes in subtype C might be explored with additional analysis of the existing data.

5. Line 234, "The ratio of negative to positively selected sites was substantially higher in the p24 than other coding regions (Fig. 3A-B). Interestingly, this was not due to a higher level of negative selection, but rather an atypically low level of positive selection (Fig 3C)." Readers (including this one) with imperfect command of evolutionary biology methods may benefit from explanation for how elevated negative selection was distinguished from decreased positive selection. Would it be correct to say that elevated negative selection is characterized by relatively high levels of synonymous mutations at a given codon or string of codons, while decreased positive selection is characterized by relatively low levels of non-synonymous mutations?

6. Line 152, "Surprisingly, despite a lower rate of intact genes, subtype C genes were not more likely to be hypermutated…." Consider clarifying why this finding is surprising. Are total numbers of defective genomes expected to scale with numbers of hypermutated genomes?

7. Line 307, "We estimate that the average number of sequences we obtained using the SMRT-UMI method is likely to

detect 99-100% of all variants with a frequency of at least 5% whereas when using Sanger SGA to obtain 20 sequences, variants present at 5% are missed 36% of the time." Consider revising the text to clarify that sequence number determines minor variant sensitivity, but sequencing method does not.

8. In Fig. 5E there is a peak of positive selection upstream the C terminus of gp120 that appears to be in stage i and is either missing in stages ii and iii or maybe covered by the stage i peak. If the peak is unique to stage i it would be of interest to describe it, otherwise it should be made clear that the peak is shared by other stages.

9. Would it be possible to display total sequence counts in addition to proportions in Supplementary Table 2?

10. Line 161 refers to Supplementary Discussion 4, but there are only 3 points in the Supp. Discussion.

11. Supplementary Fig. 6 has many sub-figures which are incorrectly marked including S3.2 and S3.6-S3.35 that should be changed to S6.2 ad S6.6-S6.35.

12. Supplementary Fig. 16 is mentioned in the Discussion but not in the
Results.

Version 1:

Reviewer comments:

Reviewer #1

(Remarks to the Author)
The authors have satisfactorily addressed the concerns raised in the previous round of review. In particular, the additional analysis comparing observed and expected numbers of mutations, together with the introduction of Supplementary Table 6, strengthens the argument that many of the identified lineages are unlikely to be explained solely by very early de novo diversification. The application of the Exact Poisson test provides a clearer statistical framework for evaluating the multilineage acquisition (MLA) assignments, and the removal of the borderline case reflects an appropriately conservative approach. Overall, these revisions improve the clarity and robustness of the study's conclusions.

I have one minor suggestion for clarification. The expected mutation counts used in the Poisson test appear to be derived from a constant-rate Poisson mutation process based on the assumed mutation rate and replication cycle time. It would be helpful if the authors briefly clarify the assumptions underlying this model, particularly whether the expectation is intended as an approximation of mutation accumulation under a constant-rate process without explicitly accounting for selective effects. Such clarification would help readers better understand the scope and interpretation of the test.

Apart from this minor point, I have no further substantive concerns. The manuscript has been substantially improved and provides a valuable contribution to our understanding of early HIV population dynamics.

Reviewer #2

(Remarks to the Author)
This reviewer thanks the authors for their responses and believes that most of the comments about the initial submission have been addressed in the rebuttal and revision.

However, this reviewer remains concerned about the validity of some multi-lineage infection determinations. Line 226 of the revised manuscript states that 47 participants were initially assigned as multi-lineage infections. If understood correctly from supplemental figure 6, these 47 included, for example, 9 from HVTN703 and 6 from HVTN704 that were supported by only one single-genome sequence in the individual. It is common in single-genome sequencing studies to treat outlying "singleton" sequences with skepticism, as these may arise by sample contamination or other technical artifacts, particularly when the total number of sequences obtained is very large. The concern here is that, if the data were presented as single-person phylogenetic trees, readers would be unconvinced about multi-lineage infection in these cases. If one were to subtract the 15 HVTN cases from the total of 47 multi-lineage infections reported in the paper, the resulting ~26% multi-lineage infection frequency would not be very different from previous reports.

Overall, this reviewer certainly does not doubt that multi-lineage infections can be missed due to undersampling. Also certainly possible that something is incorrect in this reviewer's reading. However, the impression is that the data presentation supporting this central point in the manuscript narrative still needs to be stronger.

HVTN cases in which multi-lineage infection is supported by only one single-genome sequence:

V703

1313_GP and REN
1586_GP

1689_GP
1848_GP
1889_GP
0790_REN
0865_REN
1407_REN
3000_REN

V704

0925_GP
1180_GP
2073_GP
0423_REN
0652_REN
1716_REN
2315_REN

Version 2:

Reviewer comments:

Reviewer #2

(Remarks to the Author)
This reviewer's last remain concern has been addressed by the most recent submission.

Open Access This Peer Review File is licensed under a Creative Commons Attribution 4.0 International License, which permits use, sharing, adaptation, distribution and reproduction in any medium or format, as long as you give appropriate credit to the original author(s) and the source, provide a link to the Creative Commons license, and indicate if changes were made.
In cases where reviewers are anonymous, credit should be given to 'Anonymous Referee' and the source.
The images or other third party material in this Peer Review File are included in the article's Creative Commons license, unless indicated otherwise in a credit line to the material. If material is not included in the article's Creative Commons license and your intended use is not permitted by statutory regulation or exceeds the permitted use, you will need to obtain permission directly from the copyright holder.
To view a copy of this license, visit https://creativecommons.org/licenses/by/4.0/

Reviewer #1 (Remarks to the Author):

Comments

The authors employ long-read PacBio single-molecule real-time (SMRT) sequencing with UMI correction to characterize early HIV populations across four intensively sampled cohorts. The use of SMRT-UMI substantially reduces technical artifacts associated with PCR amplification and sequencing errors, enables reconstruction of long-range haplotypes, and provides compelling evidence that early HIV populations are more complex and dynamic than previously appreciated. The study further suggests that multiple lineage acquisitions (MLA) frequently arise from the transmission of multiple viral variants from donors rather than from de novo diversification in recipients, leading to the conclusion that the number of transmitted viruses may be higher than previously estimated.

By combining advanced sequencing technologies with dense longitudinal sampling across multiple cohorts, this work significantly extends our understanding of early intra-host HIV diversity and lineage dynamics.

Response: We thank the reviewer for their positive comments on the importance of this work.

However, despite these strengths, I have two major concerns regarding the interpretation of the results.

Major Concern 1 — Distinguishing transmitted lineages from early de novo diversification in the absence of donor–recipient pairs

The combined application of Poisson Fitter and phylogenetic/distance-based analyses is a major strength of this study and represents one of the most rigorous efforts to characterize early HIV population structure to date.

Response: We thank the reviewer for this positive comment on the rigor of our work.

However, despite these methodological advances, the available data do not permit a definitive distinction between viral lineages that were transmitted at exposure and those that emerged through extremely early de novo diversification following transmission. This limitation is particularly relevant for the uncertain-origin lineages (UOLs), which differ from dominant lineages by only 1–3 substitutions.

At a more fundamental level, the lack of donor–recipient transmission pairs precludes direct assessment of whether the detected lineages were present in the donor prior to transmission. As a result, inferences regarding the frequency of multiple transmitted lineages rely exclusively on indirect evidence derived from recipient-only data. The authors are therefore encouraged to more clearly distinguish conclusions about early within-host population complexity from those about the intrinsic stringency of the HIV transmission bottleneck.

Response: We agree that donor-recipient pairs would provide the most definitive determination of the origins of variants found in the newly infected host. However, as with most studies of this nature (e.g., the meta-analysis of Baxter et al, cited in our paper, did not discriminate on the basis of the availability of donor-recipient pairs), no donor-recipient pairs were available from any of the cohorts we studied as no contact tracing was performed. We have added text to the Results (lines 91-95) to acknowledge and respond to this issue. Regarding the uncertain-origin lineages (UOLs) were just that - we could not confidently assert whether they were transmitted or evolved in the new host. This is why we reported, conservatively, what the rates of what we could confidently assert as multiple transmitted lineages in the abstract and only indicated in the body of the manuscript what those rates would have been IF the UOLs were in fact all transmitted.

The authors' interpretation appears to rely, at least implicitly, on the relatively low mutation rate of HIV (~2–5 × 10^{-5} mutations per site per replication cycle, corresponding to ~0.2–0.5 substitutions per genome per cycle), which would argue against extensive early de novo diversification. While this argument is generally reasonable, it warrants more careful consideration for several reasons. First, the estimated center of bounds (COB) corresponds to approximately seven days post-acquisition, implying that multiple viral replication cycles may have occurred before viral RNA became detectable in plasma. During this interval, each viral genome could accumulate multiple de novo mutations.

Response: We thank the reviewer for this comment and have conducted additional analyses and added text to the Results (lines 100-103 and 108-130) and a table (Supplementary Table 6) to address this concern. Assignment of each of the 15 multiple lineage infections reported from the FRESH and RV217 cohorts was strongly supported by comparing the observed versus expected number mutations as well as by the consistency of detection in both the GP and REN regions and the presence of lineage-associated indels. Using an Exact Poisson 99% CI test, we confirmed MLAs in 46 of 47 cases. We removed the 47th case as an MLA in the revised manuscript.

Second, a substantial proportion of secondary lineages in MLA cases are present at relatively low frequencies (often in the ~0.1%–10% range). Given known intra-host substitution rates and the potential for strong early selective pressures, it is biologically plausible that variants differing by only a small number of substitutions could arise rapidly through early de novo diversification following transmission. This raises the possibility that some low-frequency lineages—particularly those detected at only a single time point or in a single genomic region—may not necessarily represent independently transmitted lineages.

Response: We again thank the reviewer for this comment and have added text to the Results (lines 131-150), and to the aforementioned Supplementary table 6 (parts B-D) to addresses this concern. The table shows all 6 cases in which minor lineage sequences were represented by 1 sequence in GP and/or REN and the single additional case in which <15 mutations were found in GP and/or REN. Exact Poisson 99% CI test results were consistent with MLA in 6/7 cases, with the outlier removed as an MLA in the revise manuscript.

Overall, donor–recipient transmission pairs remain the most direct and compelling evidence for distinguishing transmitted lineages from early intra-host diversification. In the absence of such data, the authors should apply particularly careful and conservative criteria when interpreting low-frequency or minimally divergent lineages as evidence of multiple transmitted viruses.

Response: We hope that the above responses addressed these concerns adequately.

Major Concern 2 — Evolutionary forces underlying intra-host diversity and lineage turnover
The manuscript uses the FUBAR framework to infer selection acting on early HIV populations and concludes that negative selection predominates. While this observation is informative, it appears insufficient to explain the pronounced lineage fluctuations, frequent lineage turnover, and replacement of dominant lineages observed in many individuals. In multiple cases, lineages rise and fall over time rather than showing consistent directional changes, suggesting that simple unidirectional purifying selection does not fully capture the underlying evolutionary dynamics.

A key conceptual framework that is not explicitly considered is pleiotropy and fitness trade-offs across biological scales, particularly between viral fitness within a host and fitness for transmission between hosts. Extensive prior work has demonstrated that HIV variants evolving late in infection often acquire phenotypes that enhance survival and replication within the host—such as altered Env glycosylation, expanded coreceptor usage, and the ability to infect a broader range of target cells including macrophages and naïve T cells—but that these same features are disfavored during transmission. In contrast, transmitted/founder

viruses are typically characterized by CCR5 usage, high CD4 dependence, limited glycosylation, and a preference for mature CD4⁺ T cells, features that appear repeatedly selected at transmission despite extensive diversification in the donor (PMC3700278; PMC3911544). Similar patterns have been reported in SARS-CoV-2, where mutations may confer little or no fitness advantage within hosts but enhance transmissibility between hosts (e.g., PMC10521905; PMC11021471).

This body of evidence supports a model in which mutations that are advantageous—or at least tolerated—within hosts may be neutral or deleterious for transmission, and vice versa. Such antagonistic pleiotropy provides a plausible explanation for several observations in the present study: (i) the persistence of substantial intra-host diversity early after infection, (ii) large temporal fluctuations in lineage frequencies, and (iii) the frequent replacement of initially dominant lineages by variants that were previously minor. Under this model, lineage turnover does not necessarily reflect simple positive or negative selection, but rather shifting fitness landscapes shaped by cell-type availability, immune pressure, and changing ecological niches within the host. Incorporating such perspectives would strengthen the evolutionary interpretation of the observed lineage dynamics.

Response: We wish to clarify that we made no claim in the paper that the apparent predominance of negative selection we observed at the earliest times of sampling was meant "to explain the pronounced lineage fluctuations, frequent lineage turnover, and replacement of dominant lineages observed in many individuals." We agree, however, that antagonistic pleiotropy may plausibly help to explain our findings and have noted that in the Discussion (lines 304-311).

Overall, this is a technically impressive and conceptually important study that leverages advanced long-read sequencing, dense sampling, and multiple cohorts to provide new insight into early HIV population structure. The data demonstrate that early HIV populations are more diverse and dynamic than previously appreciated. Addressing the concerns raised above—particularly those related to early de novo diversification, evolutionary interpretation—would substantially strengthen the robustness, clarity, and interpretability of the conclusions.

Minor Comment — Definition of multilineage acquisition (MLA)
A more explicit operational definition of multilineage acquisition (MLA) is needed. In the main text, the authors state that the frequency of MLA was determined by considering both the GP and REN regions across all sampled time points; however, the precise criteria used to define a distinct lineage are not clearly specified. While uncertain-origin lineages (UOLs) are defined as differing from a dominant lineage by 1–3 substitutions, it remains unclear whether MLA designation required (i) a minimum number of consistent nucleotide differences across sequences, and/or (ii) a minimum relative frequency threshold at any given time point.

Response: The 4 mutation threshold we employed was mentioned in the manuscript but we have reiterated this point in the revised manuscript (lines 100-103). We hope that the responses to Major concern 1 satisfies the concern over minimum differences and frequencies.

Clarification of these criteria would improve reproducibility, reduce ambiguity in lineage classification, and facilitate comparison with prior studies investigating HIV transmission bottlenecks and transmitted/founder virus multiplicity.

Other Minor comments/suggestions, noted by Page/Line.

Response: We thank the reviewer for pointing out the following errors.

1. P5/L161: "Poisson Fitter21 (Supplementary Discussion 4)" is cited however, only Supplementary Discussions 1-3 are present in the Supplementary Information. Please verify and correct this reference.

Response: We have corrected this in the text as referring to Supplementary Discussion 3.

2. P6/L198: "(see FRESH participants 079, 271 and 318 in Fig 2, and RV217 participants 20337, 20502 and 40363 in and 318 in Fig. 2, and RV217 participants 20337, 20502, 40061, 40265 and 40436 in Supplementary Fig. 9)". The citation of participant identifiers and figure references contains apparent duplication and inconsistencies. Please carefully revise this sentence to ensure that each participant is correctly linked to the appropriate figure.

Response: We have corrected these errors in the text (lines 171-175).

3. P15/L529: "FUBAR algorithm modified to perform calculations in batch (https://github.com/MullinsLab/FUBAR_in_batch) ." The URL provided for the modified FUBAR algorithm is not accessible. Please verify the link or provide an alternative reference.

Response: This url should be functioning now.

Reviewer #2 (Remarks to the Author):

Mullins et al. used single molecule long read sequencing to analyze gag-pol (GP) and rev-vpu-env-nef (REN) regions of the HIV genome from geographically disparate cohorts of PWH at 2-5 timepoints within 61 days of infection, thus aiming to study diversity and selective forces in acute HIV infection. The major reported finding of the study is a multilineage infection rate minimally estimated at 38%, which is higher than previous studies. Other findings include fluctuations of 10-fold or more in some individuals with multiple transmitted lineages, differences in rates of hypermutated and defective genomes between HIV clades, and the identification of conserved gene regions and regions evolving under negative or positive selection in gag and env.

The study addresses an important issue using cutting-edge technology and unique sets of samples from human participants. The findings are novel and interesting, and the arguments in the paper are clear. This reviewer particularly appreciated the manuscript's treatment of "transmitted/founder" sequence terminology and its concise treatment of vaccine development implications at the end of the Discussion section.

Response: We thank the reviewer for these positive comments on our work.

This reviewer's largest concerns about the manuscript relate to strength of findings and transparency of methods. Addressing the concerns described below will help readers critically evaluate the arguments in the manuscript.

1. The manuscript should clarify the strength of findings about multilineage infection.
a. While some scatter plots in Supp. Fig. 6 strongly support multilineage infection, there appear to be 19 cases (listed below) in which multilineage infection is supported by only one sequence for the given region in the individual. Also, it seems that only one of the two regions sequenced is shown for these cases. Does this mean that multilineage infection was not seen for the other region in these cases? The manuscript should

explicitly address how it is possible to know that these lone sequences from putative secondary lineages were not due to UMI “carryover” (line 442) combined with error, to contamination, or to other artifacts. FSH_318_GP, V703_1313_GP, V703_1586_GP, V703_1689_GP, V703_1764_GP, V703_1848_GP, V703_1889_GP, V703_0790_REN, V703_0865_REN, V703_1313_REN, V703_1407_REN, V703_1789_REN, V703_3000_REN, V704_2073_GP, V704_0423_REN, V704_0652_REN, V704_1716_REN, V704_2073_REN, V704_2315_REN.

Response: We thank the reviewer for this comment. As described in our response to Reviewer 1, we have now included a new analysis using an Exact Poisson 99% CI test to evaluate our lineage assignments, and have included a new table (Supplementary Table 6) and text (lines 108-150) to describe these results including a breakdown of participants in which MLA were detected in only one of the GP or REN regions.

b. Line 430, “Westfall et al estimated PCR and sequencing error rates using this method paired with the PORPIDpipeline software to be less than 8.6 x 10-8 per base, or less than one error in every ~3700 432 REN sequences.” This text suggests that almost all sequences generated in this study are 100% correct. What about errors in reverse-transcription of sample RNA to cDNA using commercially provided enzyme? Should these not occur at rates of 10-5 – 10-4 per base, for an average of one error every 3 – 30 REN sequences? The manuscript’s treatment of error rates should be complete.

Response: We thank the reviewer for this comment. We have now described this and other potential sources of errors in lineage designations in the Methods and referred back to Westfall et al for citations (lines 425-427 and 448-451).

c. While the use of a manual inspection process to determine numbers of clades in the datasets from individual participants is certainly defensible, the manuscript could do more to illustrate how these decisions were made. Supplementary Fig. 5 is probably not accessible enough to educate readers who do not do this type of work themselves.

Response: We have added additional text (lines 100-103) to help with understanding as well as adding discussion of the newly added Poisson confirmatory analyses described above.

2. The manuscript presents data from RV217 and FRESH (total N=42 participants), with twice weekly sampling, combined with data from N=81 participants in placebo arms from the two AMP trials, with once monthly sampling. Does combining data from studies with very different sampling schedules cause any complications in analyses that relate to presumptive time since transmission? The manuscript refers to previous studies by Gilbert and Rossenkhan to describe how estimated dates of detectable infection (EDDI) were derived from HIV diagnostics and viral sequence data, but the manuscript should probably describe at least the logic of these previous analyses.

Response: We have added additional detail to the Results section (lines 54-57) and a reference to the schema used in the Rossenkhan paper.

3. Line 156, “when sample datasets with no hypermutated sequences were excluded, there was a strong trend for hypermutated sequences to decline with increasing viral loads….” In Supp. Fig. 4, it appears that the number of samples with no detectable hypermutated sequences is large, so that the reported strong trend excludes quite a bit of data. The manuscript should address this. Can anything more be said about which samples do and do not include hypermutated sequences? Do samples without hypermutants preferentially come from specific participants? Is it possible that the sensitivity of the hypermut analysis is not uniform across participants and/or clades?

Response: The analysis in Supplementary Figure 4 utilizes single samples from each participant in each time window to exclude bias due to the number of samples evaluated for each participant. We noted in the Discussion that we found no significant differences in hypermutation rates by viral Subtype and Supplementary Discussion 1 further comments on this issue.

4. Lines 343-348 attribute the higher rate of defective gag and env genes in subtype C to lower RT fidelity, because the higher rate of defective genes was not associated with higher detected rates of APBOBEC-mediated hypermutation. However, is it not also possible that subtype C sequences use more codons that are 1 single-nucleotide mutation away from stop codons? Of particular interest would be codons where transitions could result in a premature stop (i.e., CAG, CGA, CAA, and TGG). The possibility of different codon usage resulting in higher rates of defective genes in subtype C might be explored with additional analysis of the existing data.

Response: We thank the reviewer for this comment and have now added additional analyses to address this concern. As described in the Results (lines 80-85) and Discussion (lines 334-338), as well as in the new Supplementary Table 5, codon bias also does not explain this observation.

5. Line 234, "The ratio of negative to positively selected sites was substantially higher in the p24 than other coding regions (Fig. 3A-B). Interestingly, this was not due to a higher level of negative selection, but rather an atypically low level of positive selection (Fig 3C)." Readers (including this one) with imperfect command of evolutionary biology methods may benefit from explanation for how elevated negative selection was distinguished from decreased positive selection. Would it be correct to say that elevated negative selection is characterized by relatively high levels of synonymous mutations at a given codon or string of codons, while decreased positive selection is characterized by relatively low levels of non-synonymous mutations?

Response: We thank the reviewer for this comment. Yes, it is correct to say that "elevated negative selection is characterized by relatively high levels of synonymous mutations at a given codon or string of codons, while decreased positive selection is characterized by relatively low levels of non-synonymous mutations". We have added text to explain these terms (lines 196-197).

6. Line 152, "Surprisingly, despite a lower rate of intact genes, subtype C genes were not more likely to be hypermutated…." Consider clarifying why this finding is surprising. Are total numbers of defective genomes expected to scale with numbers of hypermutated genomes?

Response: Yes, we did expect the number of defectives to scale with hypermutation, although we have now removed the term "surprising" as we have performed additional analyses as mentioned in our response to point 4 above.

7. Line 307, "We estimate that the average number of sequences we obtained using the SMRT-UMI method is likely to detect 99-100% of all variants with a frequency of at least 5% whereas when using Sanger SGA to obtain 20 sequences, variants present at 5% are missed 36% of the time." Consider revising the text to clarify that sequence number determines minor variant sensitivity, but sequencing method does not.

Response: We have clarified this point in the text (lines 285-286).

8. In Fig. 5E there is a peak of positive selection upstream the C terminus of gp120 that appears to be in stage i and is either missing in stages ii and iii or maybe covered by the stage i peak. If the peak is unique to stage i it would be of interest to describe it, otherwise it should be made clear that the peak is shared by other stages.

Response: That peak, which is referred to in the text (line 229), is actually in stage iii, which is a period of time that immune driven selection should be more detectable.

9. Would it be possible to display total sequence counts in addition to proportions in Supplementary Table 2?

Response: Total sequence counts are provided by cohort in Table 1, whereas Supplementary Table 2 provides a breakdown of subtypes.

10. Line 161 refers to Supplementary Discussion 4, but there are only 3 points in the Supp. Discussion.

Response: We have corrected this in the text as referring to Supplementary Discussion 3.

11. Supplementary Fig. 6 has many sub-figures which are incorrectly marked including S3.2 and S3.6-S3.35 that should be changed to S6.2 ad S6.6-S6.35.

Response: We have corrected this in the file.

12. Supplementary Fig. 16 is mentioned in the Discussion but not in the Results.

Response: This figure shows a comparison of our results to a previous publication and thus thought it to be most appropriate to include in the Discussion.

REVIEWER COMMENTS

Reviewer #1 (Remarks to the Author):

The authors have satisfactorily addressed the concerns raised in the previous round of review. In particular, the additional analysis comparing observed and expected numbers of mutations, together with the introduction of Supplementary Table 6, strengthens the argument that many of the identified lineages are unlikely to be explained solely by very early de novo diversification. The application of the Exact Poisson test provides a clearer statistical framework for evaluating the multilineage acquisition (MLA) assignments, and the removal of the borderline case reflects an appropriately conservative approach. Overall, these revisions improve the clarity and robustness of the study's conclusions.

I have one minor suggestion for clarification. The expected mutation counts used in the Poisson test appear to be derived from a constant-rate Poisson mutation process based on the assumed mutation rate and replication cycle time. It would be helpful if the authors briefly clarify the assumptions underlying this model, particularly whether the expectation is intended as an approximation of mutation accumulation under a constant-rate process without explicitly accounting for selective effects. Such clarification would help readers better understand the scope and interpretation of the test.

Apart from this minor point, I have no further substantive concerns. The manuscript has been substantially improved and provides a valuable contribution to our understanding of early HIV population dynamics.

Response: We thank the reviewer for their positive response to the revised manuscript. Regarding the Poisson mutation process, the answer is yes, that we assumed a constant rate. While we are aware that assuming a constant mutation rate is an approximation, previous work has shown that within the initial/early window of infection, this "first order approximation" works well (citation in revised text, lines 485-488).

Reviewer #2 (Remarks to the Author):

This reviewer thanks the authors for their responses and believes that most of the comments about the initial submission have been addressed in the rebuttal and revision.

However, this reviewer remains concerned about the validity of some multi-lineage infection determinations. Line 226 of the revised manuscript states that 47 participants were initially assigned as multi-lineage infections. If understood correctly from supplemental figure 6, these 47 included, for example, 9 from HVTN703 and 6 from HVTN704 that were supported by only one single-genome sequence in the individual. It is common in single-genome sequencing studies to treat outlying "singleton" sequences with skepticism, as these may arise by sample contamination or other technical artifacts, particularly when the total number of sequences obtained is very large. The concern here is that, if the data were presented as single-person phylogenetic trees, readers would be unconvinced about multi-lineage infection in these cases. If one were to subtract the 15 HVTN cases from the total of 47 multi-lineage infections reported in the paper, the resulting ~26% multi-lineage infection frequency would not be very different from previous reports.

Overall, this reviewer certainly does not doubt that multi-lineage infections can be missed due to

undersampling. Also certainly possible that something is incorrect in this reviewer's reading. However, the impression is that the data presentation supporting this central point in the manuscript narrative still needs to be stronger.

HVTN cases in which multi-lineage infection is supported by only one single-genome sequence:

V703

1313_GP and REN
1586_GP
1689_GP
1848_GP
1889_GP
0790_REN
0865_REN
1407_REN
3000_REN

V704

0925_GP
1180_GP
2073_GP
0423_REN
0652_REN
1716_REN
2315_REN

Response: We thank the reviewer for their positive response to the revised manuscript and further attention to detail in the manuscript, explicitly for careful review of the very large Supplementary Figure 6. However, it appears that the point that we removed recombinants prior to generating that figure (line 102) was missed by the reviewer. We hope to allay the concerns the reviewer raises from this and other considerations, as follows, as we re-evaluated each of the cases indicated by the reviewer and find strong support for our designations, as described below.

First, as reiterated above, we removed all recombinants prior to generating the plots shown in Supplementary Figure 6. The presence of recombinants provides clear evidence of the lineages the reviewer calls into question. Recombinants between the major and minor lineages were found in 15 of the 17 cases noted by the reviewer and shown in parentheses below.

Second, as noted in the Methods section of the paper, we removed 15% (20% for V703 REN) of the sequences, from the smaller end of the family size distributions (above our minimal family size cutoff), to very conservatively remove most if not all sequences found due to cDNA primer carryover in the PCR steps. This had the effect of discarding a large number of real sequences as well as having the effect of reducing the number of members within lineages. This removal step had the effect of making it appear that only a single sequence was found to represent a lineage in some cases. However, the total number of sequences found to represent the minor lineage was often greater than one, with the actual number (N) shown in parentheses below. This was also detected in one of the 2 remaining cases noted by the reviewer.

Finally, in the last case noted by the reviewer, V704_0652_REN, neither recombinants nor additional sequences were found to represent the minor lineage prior to applying conservative filtering. However, this lineage was distinguished by the presence of 7 clustered, unique

mutations. In our experience, there is no precedent for such an outlier being derived from a technical artifact.

Regarding possible contamination, all of the sequences in this project were compared to all other sequences from this and all other projects derived from our laboratories as well as in the literature using the PORPIDpipeline tool for each sequencing run and at the end of project sequencing, an in-house Contamination Detection Toolkit using BLAST (Williamson lab), and the Viroblast tool the Mullins lab developed specifically for this purpose (now referenced in the text). We recorded the most closely related sequence found in our project databases. The Mullins laboratory has a long history of the study of epidemiologic linkage and tracking down HIV sample contamination and mixups (e.g., see references below) and after rigorous review, no instances of sample contamination were observed.

As noted by the reviewer, multiple lineages can be missed by under sampling. However, as sampling increases, minor variants can be picked up, one-by-one, i.e., we would not expect them to be present in large numbers.

In summary, we have retained each of the lineage designations put forward in the initially revised manuscript. We have added Supplementary Discussion 4 (line 104) to emphasize these points, as well as elaborated our contamination check protocols in Methods (lines 445-450).

V703

1313_GP (plus 3 recombinants) and REN (plus 2 recombinants)
1586_GP (plus 8 recombinants)
1689_GP (plus 2 recombinants)
1848_GP (plus 4 recombinants)
1889_GP (N=2 plus 1 recombinant)
0790_REN (N=8 plus 2 recombinants)
0865_REN (N=2 plus 6 recombinants)
1407_REN (N=7)
3000_REN (N=2 plus 20 recombinants)

V704

0925_GP (plus 2 recombinants)
1180_GP (plus 4 recombinants)
2073_GP (plus 1 recombinant)
0423_REN (N=3 plus many recombinants)
0652_REN
1716_REN (N=2 plus 4 recombinants)
2315_REN (plus 5 recombinants)

References:

Ou, C. Y., Ciesielski, C. A., Myers, G., Bandea, C. I., Luo, C. C., Korber, B. T., Mullins, J. I., Schochetman, G., Berkelman, R. L., Economou, A. N., and et al. (1992), 'Molecular epidemiology of HIV transmission in a dental practice', *Science,* 256 (5060), 1165–71.

Korber, B. T., Learn, G., Mullins, J. I., Hahn, B. H., and Wolinsky, S. (1995), 'Protecting HIV databases', *Nature,* 378 (6554), 242–4.

Learn, G. H., Jr., Korber, B. T., Foley, B., Hahn, B. H., Wolinsky, S. M., and Mullins, J. I. (1996), 'Maintaining the integrity of human immunodeficiency virus sequence databases', *J Virol,* 70 (8), 5720–30.

Frenkel, L. M., Mullins, J. I., Learn, G. H., Manns-Arcuino, L., Herring, B. L., Kalish, M. L., Steketee, R. W., Thea, D. M., Nichols, J. E., Liu, S. L., Harmache, A., He, X., Muthui, D., Madan, A., Hood, L., Haase, A. T., Zupancic, M., Staskus, K., Wolinsky, S., Krogstad, P., Zhao, J., Chen, I., Koup, R., Ho, D., Korber, B., Apple, R. J., Coombs, R. W., Pahwa, S., and Roberts, N. J., Jr. (1998), 'Genetic evaluation of suspected cases of transient HIV-1 infection of infants', *Science,* 280 (5366), 1073–7.

Learn, G. H. and Mullins, J. I. (2004), 'The microbial forensic use of HIV sequences', in T. Leitner, et al. (eds.), *HIV Sequence Compendium 2003* (Los Alamos, N. M.: Los Alamos National Laboratory, Theoretical Biology and Biophysics), 22–37.
